# Ligand Radicals Tune LPMO Activity in Model Complex

**DOI:** 10.1021/acs.inorgchem.5c05285

**Published:** 2026-02-26

**Authors:** Caterina G. C. Marques Netto, Ritika Pandey, Caio Bezerra de Castro, Larissa Moreno, Millena Pereira Ferreira, Lullie Gomes Rodrigues, Walber Gonçalves Guimaraes, João Honorato de Araujo-Neto, Gabrielle Conciani, R. Brian Dyer, Sergio A. V. Jannuzzi, André F. de Moura, Dulce H. Ferreira de Souza, Otaciro R. Nascimento

**Affiliations:** † Department of Chemistry, 1371 Emory University, 1515 Dickey Drive, Atlanta, Georgia 30322, United States of America; ‡ Departamento de Química, 67828 Universidade Federal de São Carlos (UFSCar), Rod. Washinton Luiz, s/n km 235, São Carlos, SP CEP 13565905, Brazil; § Instituto de Química, Departamento de Química Fundamental, Universidade de São Paulo (USP), Av. Prof. Dr. Lineu Prestes, 748, São Paulo, SP CEP 05513-970, Brazil; ∥ Max Planck Institute for Chemical Energy Conversion, Stiftstr. 34−36, Mülheim an der Ruhr 45470, Germany; ⊥ Instituto de Física de São Carlos, Universidade de São Paulo (USP), Av. Joao Dagnone, 1100, Sao Carlos, SP CEP 13563-120, Brazil

## Abstract

Radicals are essential to the catalytic chemistry of metalloenzymes, enabling reactivity and self-protection through controlled redox processes. In copper-dependent LPMOs, amino acid radicals mediate oxidative transformations via hole hopping. However, the generation and role of ligand-centered radicals in synthetic copper models remain poorly understood. Here we show that an l-proline–based N,N,O,O-coordinated copper complex (**4**) generates stable carbon-centered ligand-radicals. A markedly higher population of these ligand radicals was observed in this complex than in the N,N,N-coordinated analogues (complexes **1–3**). Catalytically, complex **4** outperforms complexes **1–3**, effectively degrading 4-nitrophenyl-β-d-glucopyranoside, cellobiose, and cellulose using either H_2_O_2_ or O_2_. The superior performance of complex **4** is linked to its lower steric hindrance, a key factor in LPMO mimicry. These results establish ligand-centered radicals as key functional analogues of enzymatic redox pathways and offer a blueprint for designing self-protecting copper oxidation catalysts.

## Graphical abstract

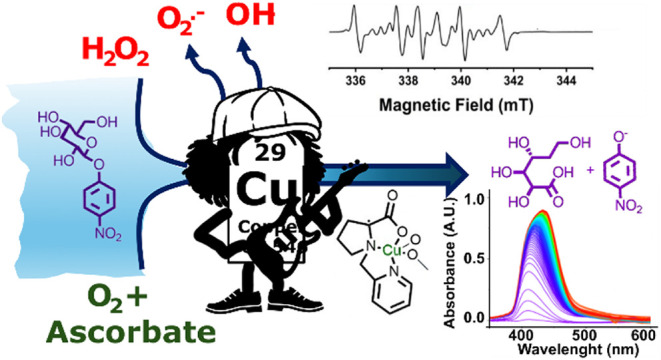

## 1 Introduction

Radicals play a crucial role in the catalytic mechanisms of several metalloenzymes, where metal centers facilitate the formation and stabilization of these highly reactive species.^1^ Among the various metal ions found in metalloenzymes, copper is particularly effective at supporting radical chemistry.^2,3^ Notable examples include tyrosyl radicals in galactose oxidase,^4^ cytochrome c oxidase,^5^ and heme-copper oxidases.^6^ In addition to stabilizing tyrosyl radicals, copper can participate directly in radical-mediated substrate activation, as observed in the catalytic mechanisms of nitrite reductase, amine oxidases, and superoxide dismutase.^2^ In these systems, radical species play central catalytic roles, often enabling otherwise challenging redox transformations.^7^

A relevant copper-dependent enzyme is lytic polysaccharide monooxygenase (LPMO). This enzyme family drives lignin-carbohydrate complex degradation^8–10^ by cleaving the β-(1→4)-linked glucan chain through the oxidation of C1 or C4 atoms using hydrogen peroxide or oxygen as cosubstrates (Figure 1).^11–13^ These oxidative reactions enhance saccharification efficiency, underscoring the significance of LPMOs in second-generation biorefineries.^14–18^ Interestingly, radicals such as histidyl, tryptophanyl, and tyrosyl have been detected in LPMO using stopped-flow techniques and electron paramagnetic resonance (EPR) spectroscopy.^19^ However, in contrast to Cu­(II)-tyrosyl radical species in other metalloenzymes, the formation of this species in LPMO is associated with an inactive enzyme in terms of saccharide oxidation.^20^ The role of the Cu­(II)-tyrosyl in LPMO has been proposed to assist in electron–hole separation,^21,22^ a mechanism that may protect the enzyme from highly reactive intermediates by deactivating them.^23,24^ This protective mechanism is especially important in LPMO catalysis, where activity depends on the formation of a reactive copper center upon activation of O_2_ or H_2_O_2_.^25–27^ For example, the formation of hydroxyl radicals as a result of H_2_O_2_ homolysis is used to form a Cu­(II)-oxyl species,^15^ key in hydrogen atom abstraction (HAA) from the strong C–H bond (>100 kcal/mol) of the saccharide.^28,29^ Thus, the proposed hole-hopping mechanism in LPMO involves the formation of tyrosine and tryptophan radicals, stabilizing the enzyme and preventing oxidative degradation when the substrate is not present.^21^

**Figure 1:**
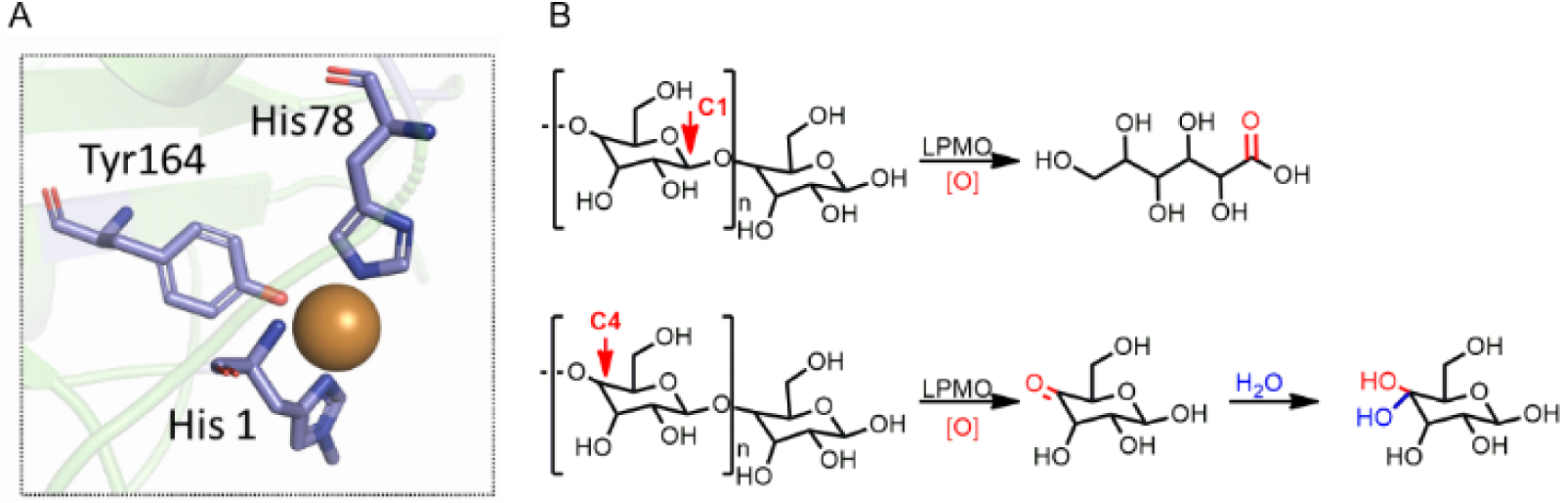
Active site structure of LPMO from *Panus similis* (PDB: 5ACH) A) and reactions catalyzed by LPMO in glycosidic substrates. B) Positions of the polysaccharide chain that are oxidized by LPMO and their relative oxidation products.

Metal complexes that mimic the active sites of metalloenzymes are well documented and serve as powerful tools for both mechanistic studies and the development of alternative catalysts.^30–32^ These biomimetic compounds aim to combine the attractive features of enzymes, such as high efficiency and selectivity, while overcoming their inherent limitations, including poor stability under nonphysiological conditions (e.g., elevated temperatures and organic solvents).^33^ The active site of LPMOs consists of a copper ion coordinated by a conserved histidine brace, forming an N,N,N-donor coordination environment (Figure 1).^34^ In light of this, various copper complexes have been designed to replicate the histidine brace motif of LPMOs, exhibiting the ability to activate dioxygen or hydrogen peroxide.^35–39^ These mimics have demonstrated activity in aerobic oxidations^40–42^ and in the cleavage of glycosidic bonds, even in recalcitrant substrates such as cellulose, chitin, or bagasse.^43–47^ Despite these advances, the phenomenon of hole hopping remains underexplored in synthetic mimics.

Recently, we reported a series of l-proline-derived copper complexes capable of generating ligand-centered radicals via a tautomerization followed by oxidation mechanism.^48^ Building on this observation, we aimed to investigate the presence and role of such radicals in copper complexes designed to mimic LPMO activity, and to evaluate their impact on biomimetic catalysis. Specifically, we focused on evaluating how these radicals influence biomimetic oxidation reactions. To this end, we examined a series of previously reported copper complexes from our group that feature an N,N,N coordination environment (complexes **1–3**, Figure 2).^49^

**Figure 2:**
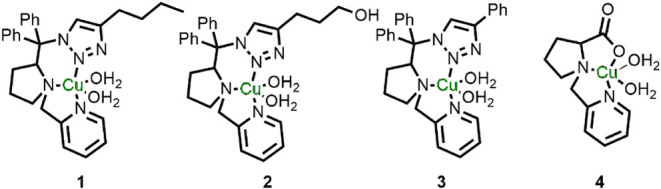
Copper complexes that were employed in this work. Complexes **1–3** were reported elsewhere^49^ and complex **4** was synthesized and characterized in this work.

Additionally, given that LPMOs can activate dioxygen, we expanded this series by designing and synthesizing complex **4**, inspired by an iron complex known to react with O_2_.^50^ Complex **4** exhibited a significantly higher population of ligand-centered radicals compared to complexes **1–3**. Two distinct carbon-based radicals were detected in complex **4**, suggesting a sequential relationship in which the formation of one radical was dependent on the presence of the other, effectively mimicking the hole hopping mechanism proposed for LPMOs. Strikingly, complex **4** outperformed complexes **1–3** in catalytic activity, and the underlying reasons for this enhancement are explored in detail in this work.

## 2 Results and Discussion

### 2.1 Copper Complexes Form Ligand-Centered Radical Species via Distinct Mechanisms

Four different complexes derived from l-proline were synthesized featuring a T-shaped ligand coordinated to the copper center similar to the histidine brace. The synthesis and characterization of complexes **1–3** (Figure 2) have been described elsewhere.^49^ These complexes provide an N,N,N coordinate environment which is completed with two water molecules as described previously.^49^ In contrast, complex **4** is less bulky and presents an N,N,O,O coordination environment (Figure 2). Interestingly, the structure of solid complex **4** determined by single crystal X-ray crystallography is a 1D polymer as shown in Figure 3. The solid state FTIR spectrum of **4** (Figure S1) presents bands at 1651 cm^–1^ (ν_as_ C–O) and 1446 cm^–1^ (ν_s_ C–O). The splitting Δν­(ν_as_-ν_s_) suggests a carboxylate group in bridging coordination mode,^51^ as observed in the X-ray structure. Complex **4** in water has a ligand centered UV–vis absorbance band at 254 nm (ε = 2304 L mol^–1^ cm^–1^), most probably a π-π * transition (Figure S2A). The d–d transition band is located at 668 nm (ε = 202 L mol^–1^ cm^–1^), consistent with a square pyramidal geometry in solution. In contrast, a new band emerges at 289 nm (ε = 1819 L mol^–1^ cm^–1^) when **4** is dissolved in carbonate buffer (pH 10.5) (Figure S2A). This new band is tentatively assigned to a ligand to metal charge transfer due to the deprotonation of the aquo complex to form a hydroxo-copper species. In addition to the new band, the peak maximum of the d–d transition band redshifts to 694 nm (ε = 88 L mol^–1^ cm^–1^). Monitoring of the d–d transition maximum versus the pH (Figure S2C and D) revealed a p*K*_a_ of 7.9 associated with the coordinated water molecule. Thus, upon deprotonation of the coordinated water, it is postulated that a hydroxo species is formed at pH > 7.9.

**Figure 3:**
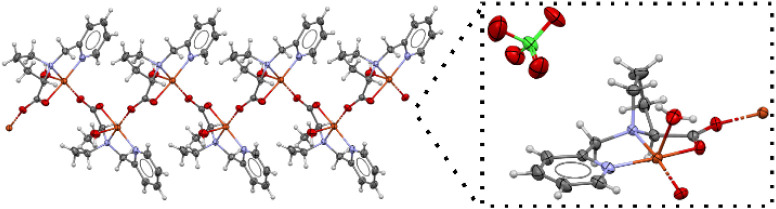
ORTEP drawing of the X-ray crystal structure of complex **4**, evidencing the 1-D polymerization. The enlarged view shows that the coordination sphere of the copper center includes the N,N,O,O donors from the ligand, a water molecule, and an oxygen atom from the bridging carboxylate moiety.

The X-band EPR spectrum of **4** acquired at 30 K in 100 mM carbonate buffer pH 10.5 (Figure S3) shows a resolved axial Cu­(II) signal with g_z_ > g_y_ ≈ g_x_ > g_e_ consistent with the tetragonal symmetry of the metal site observed in the crystal structure and with the unpaired electron in the dx^2^–y^2^ orbital. The g-values of 2.06, 2.09, and 2.27 and the^63,65^ Cu (I = 3/2) hyperfine coupling constants |A| = [16.7, 31.4, 176.8]×10^–4^ cm^–1^ are similar to values previously reported for similar proline-based Cu­(II) complexes^52^ as well as for a Cu­(II) *bis*-imidazole-amine complex in aqueous solution.^53^ It is also noteworthy that these spin Hamiltonian parameters are similar to those of LPMO NcAA9C (gz = 2.27 and |Az| = 152 × 10–^4^ cm^–1^).^54^ The Peisach-Blumberg correlation^55^ is consistent with N/O coordination, indicating that in solution the labile equatorial and the axial site are occupied by solvent, in agreement with the observations from X-ray crystallography. The small rhombicity in the g and A tensors is attributed to the inequivalent ligation along the x and y axes on the equatorial plane imposed by the T-shaped tridentate ligand. Although complex **4** is polymeric in the solid state, we postulate that in solution complex **4** behaves as a single molecule and not as a dynamic polymer. The lack of deviation from the Beer–Lambert linearity at high concentrations in the UV–vis spectroscopy^56,57^ (Figure S4) and the room-temperature EPR τ_corr_ are both inconsistent with formation of large aggregates^52^ (Figure S2D).

The spin integration of 1.0 mM and 0.1 mM solutions of complex **4** at 300 K revealed 70% and 68% of the nominal concentrations, respectively. An independent measurement of a 1 mM solution at 30 K also integrated to a lower concentration, further corroborating the formation of EPR-silent species. Weakly antiferromagnetic coupled species have been reported in solution and in the solid state as a consequence of the dimerization of the Cu­(II) complexes with flat T-shaped bis-imidazole-amine ligands.^53^ However, that could not be the case for complex **4**, as it was shown to be monomeric in solution.

Since complex **4** is unlikely to exist as a polymer in solution, an alternative explanation for the reduced spin integration could be the presence of radical species that couple antiferromagnetically with the copper center.^58^ Thus, we added DMPO (5,5-dimethyl-1-pyrroline N-oxide) as a radical trapping agent to a solution of complex **4** and measured the EPR spectra. Two carbon-centered radical species were observed by spin trapping, as shown in Figure 4A. Given that complex **4** was synthesized and handled under aerobic conditions, the formation or observation of Cu­(I) species was not expected, and the maintenance of the Cu­(II) pattern is in agreement with that (Figure S5).

**Figure 4:**
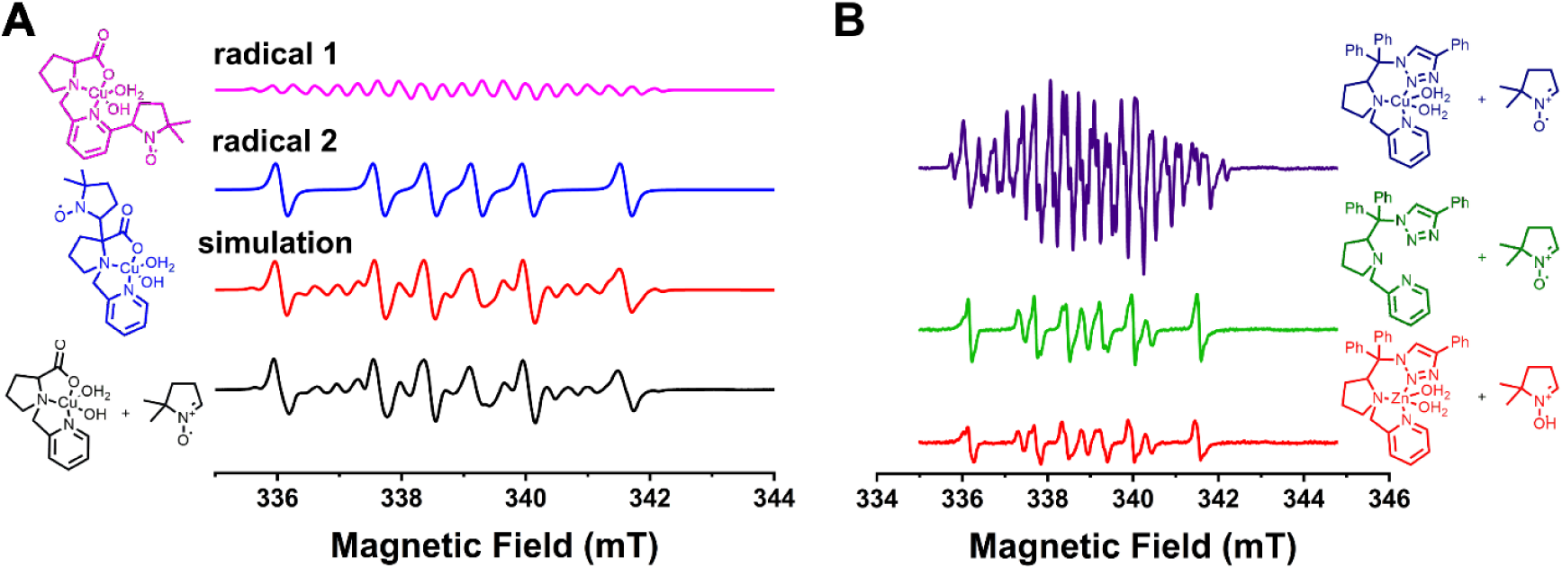
EPR spectra of DMPO-trapped radicals. A) Complex **4** in the presence of DMPO. The experimental DMPO-trapped radicals (black line) agree with the sum of simulated spectra (red line) of both radical 1 (pink line) and radical 2 (blue line). B) Complex **3** (dark blue), complex **3-Zn** (red) and **3-ligand** (green) in the presence of DMPO. The spectra were recorded using an X-band Varian spectrometer, model E-109 at room temperature and quartz flat cell with a scan range of 10 mT, field modulation of 0.1 mT, microwave power of 5 mW. The EasySpin program was utilized to perform the spectral simulation. The EPR measurements in complex **3** were performed at 0.02 mT to evidence the hyperfine structure of the radical 1. Integration of the radical signals was performed using a Cr­(III) standard, obtaining an integration of 6% of radical in **3**, and 1% of radicals in **3-ligand** and **3-Zn**.

The first radical (radical 1) corresponds to 32.8% of the radical composition and is consistent with a carbon-centered radical near three inequivalent hydrogens and one nitrogen (g = 2.0061, a_H_ = 13.79 G, a_N_ = 16.95 G and a_H1_ = 3.35 G, a_H2_ = 4.29 G, a_H3_ = 3.50 G, a_N1_ = 3.17 G).^58^ The second one (radical 2) (67.2% of the radical composition) is also carbon-centered, with parameters of g = 2.0064, a_H_ = 24.37 G, and a_N_ = 15.81 G. Based on the observed hyperfine coupling pattern, we assign radical 1 as being centered on the pyridine ring. Particularly, the presence of a second ^14^N hyperfine coupling of ∼2–4 G is diagnostic of partial localization of unpaired spin density on the heteroaromatic nitrogen, together with delocalization over the aromatic ring hydrogens. This assignment is further supported by DFT calculations, which show spin density delocalization over the pyridine framework (Figure S6), in good agreement with the experimentally observed complex hyperfine splitting. In contrast, radical 2 appears to be centered on the pyrrolidine moiety; the absence of resonance delocalization in this fragment results in a simpler spectrum, as shown in Figure 4. Integration of the total radical spins reveals that ≅15% of complex **4** is composed of radicals. This finding reveals that the ligand can efficiently stabilize radicals in more than one fashion and underscores the role of the pyrrolidine ring as a radical generator and the pyridine ring as a “hole-storage” site. The presence of radicals may account for the discrepancy between the expected and observed Cu­(II) concentrations in the EPR spectra, as they can undergo antiferromagnetic coupling with the d^9^ Cu­(II) center, leading to the formation of EPR-silent species.

To test whether radicals are a general feature of the speciation of the complexes in solution, we also examined the EPR spectra of one of the triazole-bearing complexes (complex **3**) with DMPO as a trapping agent. Interestingly, this complex exhibit ≅6% of radical species in the DMPO-trapped EPR experiments (Figure 4B). These radicals are a mixture of a carbon-centered DMPO-trapped radical, and free radical from the triazole ring. As radicals were observed in both complex **4** and **3**, this indicates that copper might be inducing the formation of radicals in these complexes. To verify that, a zinc-version of complex **3** (**3-Zn**) and the ligand of **3** (**3-ligand**) exhibited only ≅1% of radical species (Figure 4B), supporting that copper has a role in the generation of these radicals.

Analysis of the XPS of these species (**3**, **3-Zn**, **3-ligand** and **4**) shows a consistent decrease in the C 1s binding energy upon complexation with copper (Figure 5). For instance, C–N and C=N in **3-ligand** shifted from 286.8 and 289.6 eV to 286.4 and 288.2 eV in **4**. Complex **3** showed these bands at 286.5 and 288.6 eV, respectively, whereas in **3-Zn** the C–N band was almost not shifted (286.7; 288.4 eV). Additionally, the N 1s energy increases more upon copper complexation (Figure 5), shifting from 399 eV in the ligand to 400.1 eV in **3** and **4**. Thus, we can infer that coordination to copper increases the binding energy in N 1s, whereas C 1s has a decrease in binding energy, which could be linked to a higher percentage of radical formation. The valence-to-core X-ray emission spectra (VtC XES) of complexes **1–3** indicated that the ligand does not have a major effect on the valence emissions (Figure S6), indicating a similar N,N,N coordination environment. The intensities of the Kβ 2,5 lines shift to lower energy in **4** owing to the O­(2p) contribution in the valence orbitals (Figure S6). Interestingly, XPS of complex **3** exhibits 15% of Cu­(I) in Cu 2p, whereas complex **4** is only composed of Cu­(II) (Figure S7). The observation of Cu­(I) in **3** might be an artifact as this complex exhibits a low Cu­(II)/Cu­(I) redox potential.^49^ These experiments reveal chemical shifts that reflect variations in the local charge density around atoms, which can be correlated with the electronic distribution obtained from HOMO–LUMO calculations (Figures S8–S10, Tables S2–S3). For instance, the HOMO in complexes **3** and **4** is mainly localized on the copper center (approximately 78% in **3** and 84% in **4**), whereas the LUMO is predominantly distributed over the pyridine rings (64% in **3** and 79% in **4**). In complex **3**, the LUMO extends over both the pyridine and triazole rings. In contrast, in **3–Zn**, the HOMO is centered on the triazole ring. In addition, a Wiberg/Mayer bond order analysis indicates a highly activated bond between Cu and the pyridine nitrogen, with values of 0.2, whereas **3-Zn** has a less pronounced value (0.4) (Table S2). This is consistent with the lower C 1s and N 1s binding energies observed for **3–Zn** compared to **3** and **4**. Thus, copper coordination removes electron density from the ligand, potentially facilitating the formation of a ligand-centered radical.

**Figure 5:**
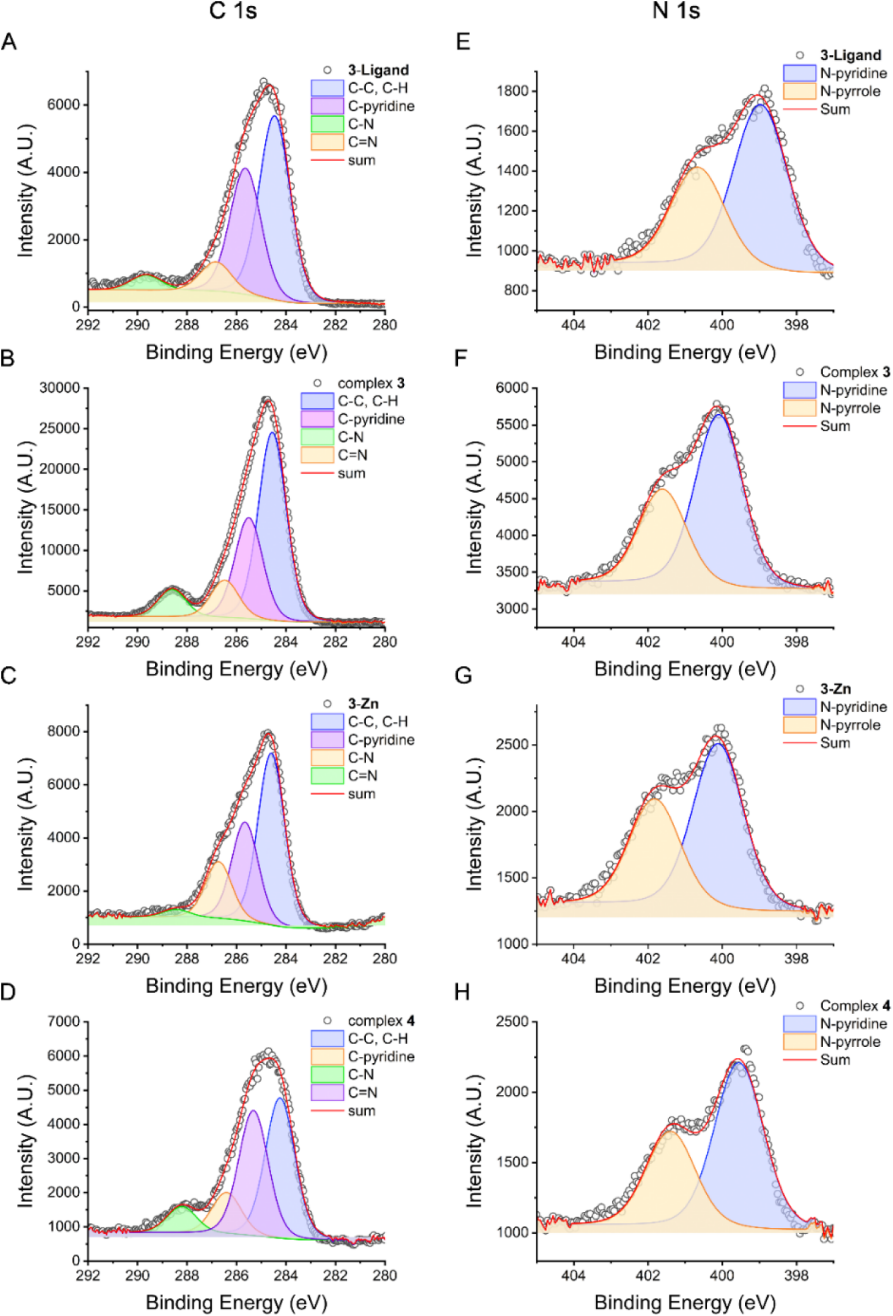
XPS of C 1s and N 1s binding energies and spectral deconvolution for **3-ligand**, **3-Zn**, **3** and **4**. A) C 1s XPS of **3-ligand**; B) C 1s of complex **3**; C) C 1s of complex **3-Zn**; D) C 1s of complex **4**; E) N 1s of **3-ligand**; F) N 1s of complex **3**; G) N 1s of complex **3-Zn**; H) N 1s of complex **4**.

One possible mechanism of radical formation involves tautomerization (Scheme 1), whereby a Cu­(I)–ligand radical is generated if the oxidized ligand form is thermodynamically accessible within the Cu­(II)/Cu­(I) redox window.^48,59^ To test this hypothesis, complex **4** was characterized by cyclic voltammetry under anaerobic conditions (Figure 6). A reduction process at E_pc_ = −500 mV corresponds to the reduction of Cu­(II) to Cu­(I), while a broad, poorly defined process is observed at E_pa_ = −195 mV which suggests an electrochemical step followed by a rapid chemical event. This behavior indicates that once Cu­(I) forms, it promptly transfers an electron to the ligand. Cu­(II)–amino acid complexes were reported to generate carboxyl radicals upon metal photoreduction to Cu­(I).^60^ Thus, the chemical process that follows the electrochemical reduction of the metal center is most probably the formation of the carboxyl radical, as shown in Figure 6B. We hypothesize that the presence of the ligand radical in **4** enables the recombination of radicals to generate a “carbene-like” species, which has a visible Cu­(I)/Cu­(II) oxidation wave at −413 mV, as observed in Figure 6A. Another anodic process is observed at −20 mV once this species is formed, this most probably corresponds to the oxidation of the “carbene-like species” to the radical ligand species. Thus, the nonreversible event at −20 mV is assigned to a ligand-centered process and the E1/2 of Cu­(II)/Cu­(I) for complex **4** is calculated as −456 mV.

**Scheme 1:**
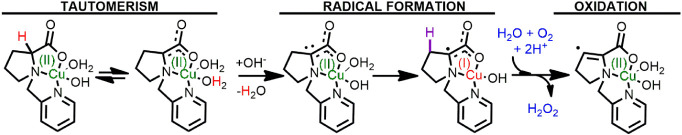
Representation of Radical Formation in Complex **4** ^a^

a A tautomerization of the L-proline moiety enables ligand deprotonation. The carbanion-like reduces the copper center to generate radical 2, concurrently oxidizing the metallic center. The green color in copper is used to represent Cu­(II) and the red color in copper is used to represent Cu­(I).

**Figure 6:**
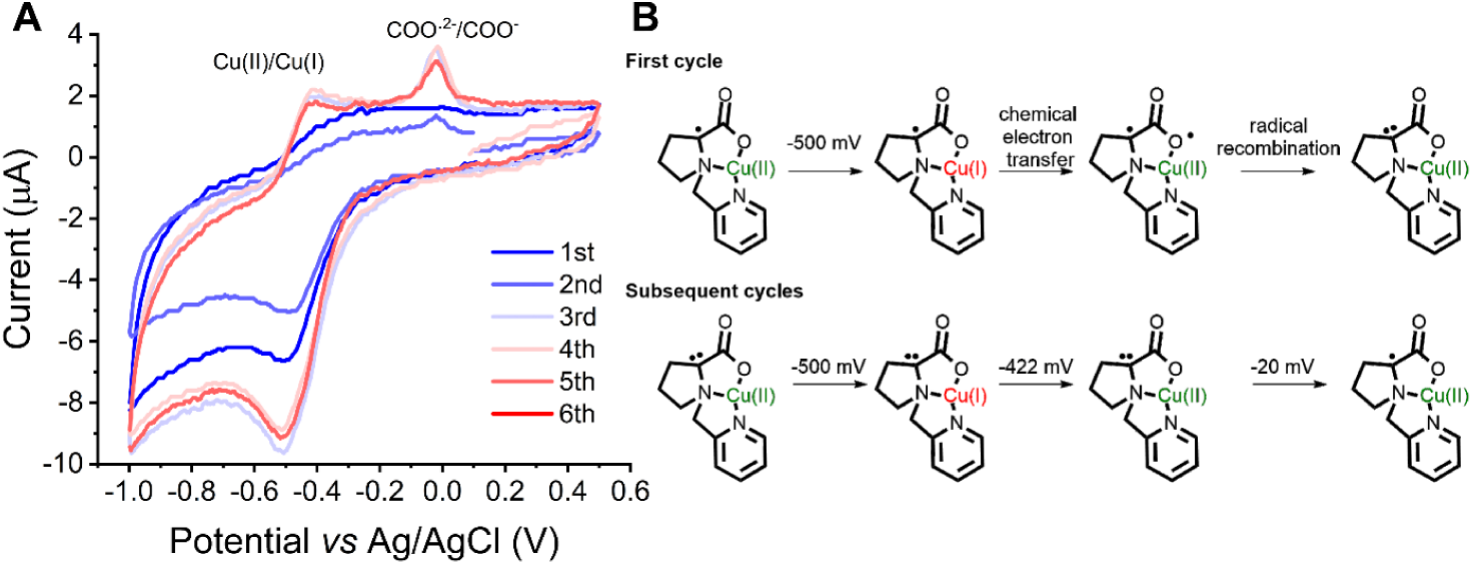
A) Cyclic voltammogram of complex **4** in carbonate buffer pH 10.5 0.1 M at 100 mVs^–1^ scan rate. A cell containing three electrodes was employed: vitreous carbon (WE), platinum (CE) and Ag/AgCl 3.5 M (reference). The black voltammogram was recorded initially. After some cycles, the red voltammogram was recorded, indicating changes in the structure of the complex associated with the reduction of Cu­(II) to Cu­(I). B) Suggestion of electrochemical and chemical processes that are associated with the cyclic voltammogram of complex **4**.

Tautomerization in l-proline ligands is rarely reported, and formation of the l-proline enolate has been calculated to require a barrier of 19 kcal/mol.^61^ Nevertheless, the ligand of complex **4** (**4-Ligand**) generates ∼1% carbon-centered radicals in carbonate buffer (pH 10.5), whereas no radicals are detected in water at pH 7 (Figure S11), indicating that radical formation is driven by pH-dependent deprotonation events. Deprotonation at the α-carbonyl position produces a delocalized carbanion, a reactive intermediate prone to interactions with Brønsted acids or metal electrophiles.^62^ Thus, while tautomerization is energetically hindered, copper coordination can stabilize this carbanion, favoring α-carbon deprotonation and promoting double-bond formation between the α and β carbons.^63^ An analysis of HRMS of the complex supports this mechanism as a peak with *m*/*z* 314.0320 (C_12_H_15_CuN_2_O_4_ calc. *m*/*z* 314.0327) is present (Figure S12), indicating that two protons from the carbon chain are absent. The presence of peaks corresponding to decarboxylated species, such as the strong peak at *m*/*z* 224.0369 (calc. *m*/*z* for C_10_H_13_CuN_2_ is 224.0374) is consistent with the Cu­(I) electron transfer to the carbonyl moiety, generating a ligand radical that undergoes decarboxylation, as stated by Lin et al.^60^ Dioxygen is expected to enhance radical formation, as shown in Scheme 1, and indeed, complex **4** synthesized anaerobically (**4­(Ar)**) lacks the absorption bands at 372 and 500 nm observed in **4** which we assign to radical species (Figure S13). Comparable UV–vis features have been reported for LPMO tyrosyl and tryptophanyl radicals at 412 and 505 nm.^23^ Further, DMPO-trapping EPR experiments confirm a significantly lower radical yield (≈1%) in **4­(Ar)** supporting tautomerization followed by oxidation as a plausible route for Cu­(II)-radical generation. Further support for the proposed tautomerization mechanism was obtained from DMPO spin-trapping experiments conducted on copper complexes structurally analogous to complex **4**. Removal of the carbonyl functionality afforded an alcohol-based analogue (**4-alcohol**), which, lacking an α-carbonyl group, did not yield any detectable carbon-centered radicals in solution. In contrast, the corresponding amide derivative (**4-amide**) produced DMPO adducts consistent with carbon-based radical intermediates (Figure S14). Moreover, the zinc analogue of complex **4** (**4­(Zn)**) shows no radicals, in agreement with Zn^2+^ being redox-inactive due to its filled d-shell (Figure S15). Therefore, because the ligand undergoes tautomerism and can generate ligand-centered radicals at high pH, we propose that the equilibrium Cu­(II)–ligand ⇌ Cu­(I)–ligand­(radical) represents a plausible pathway for generating the Cu­(II)–ligand­(radical) species in aerobic medium. In this scenario, any transiently formed Cu­(I) would be readily and rapidly reoxidized to Cu­(II), making its direct detection unlikely. Collectively, these findings support a copper-dependent tautomerization/deprotonation pathway followed by oxidation as the origin of ligand radical formation, as shown in Scheme 1.

### 2.2 Reactivity toward O_2_ and H_2_O_2_

LPMOs catalyze the oxidative cleavage of C–C bonds in lignocellulose using molecular oxygen or hydrogen peroxide as cosubstrates. Notably, when dioxygen is the oxidant, LPMOs first convert it into hydrogen peroxide, thereby functioning in practice as peroxygenases.^64^ Motivated by this, we investigated the reactivity of our complexes with H_2_O_2_ and O_2_.

Dioxygen is transformed into hydrogen peroxide in the enzyme via a Cu­(I) intermediate, conveying the monooxygenase activity.^65,66^ Complexes **1–3** have already been shown to form a Cu­(I) state in the presence of sodium ascorbate.^49^ Complex **4** Cu­(II)/Cu­(I) has E1/2 at −456 mV (vs Ag/AgCl 3.5 mol L^–1^) and we envisioned that the Cu­(I) state of **4** can also be partly reached using the same reducing agent (Asc^•^/Asc^–^ potential at −300 mV vs Ag/AgCl (3.5 mol L^–1)^).^67^ Confirmation of the reduction of copper in complex **4** upon ascorbate addition was obtained from the disappearance of the d–d transition band (Figure S16A) and by the decrease in Cu­(II) pattern detected by EPR, suggestive of the formation of an EPR silent molecule (Figure S16B). The increase of ascorbate concentration in the EPR analysis revealed an increase in Cu­(II) reduction, which is consistent with a kinetically driven redox reaction.^67^ In the presence of dioxygen and ascorbate the intermediate **4ox1** is formed, which can be detected by UV–vis at low temperatures (5 °C) in aerobic medium due to the emergence of bands at 405 and 480 nm (Figure 7B). This intermediate was shown to be unstable as these bands decayed with the increase of temperature (Figure S17). The positioning of these bands is consistent with the formation of transient end-on superoxocopper­(II) and hydroperoxo-copper­(II) complexes, which exhibit LMCT bands near 375 nm.^68–70^ For instance, an intense charge transfer absorption band at ≅410 nm is observed in end-on superoxo complexes in addition to two weaker features at 600 and 750 nm.^71^ On the other hand, side-on mononuclear superoxo complexes have bands at ≅500 and 600 nm.^71^ Definitive identification of the intermediate species via Raman spectroscopy was not possible despite several attempts, due to fluorescence.

**Figure 7:**
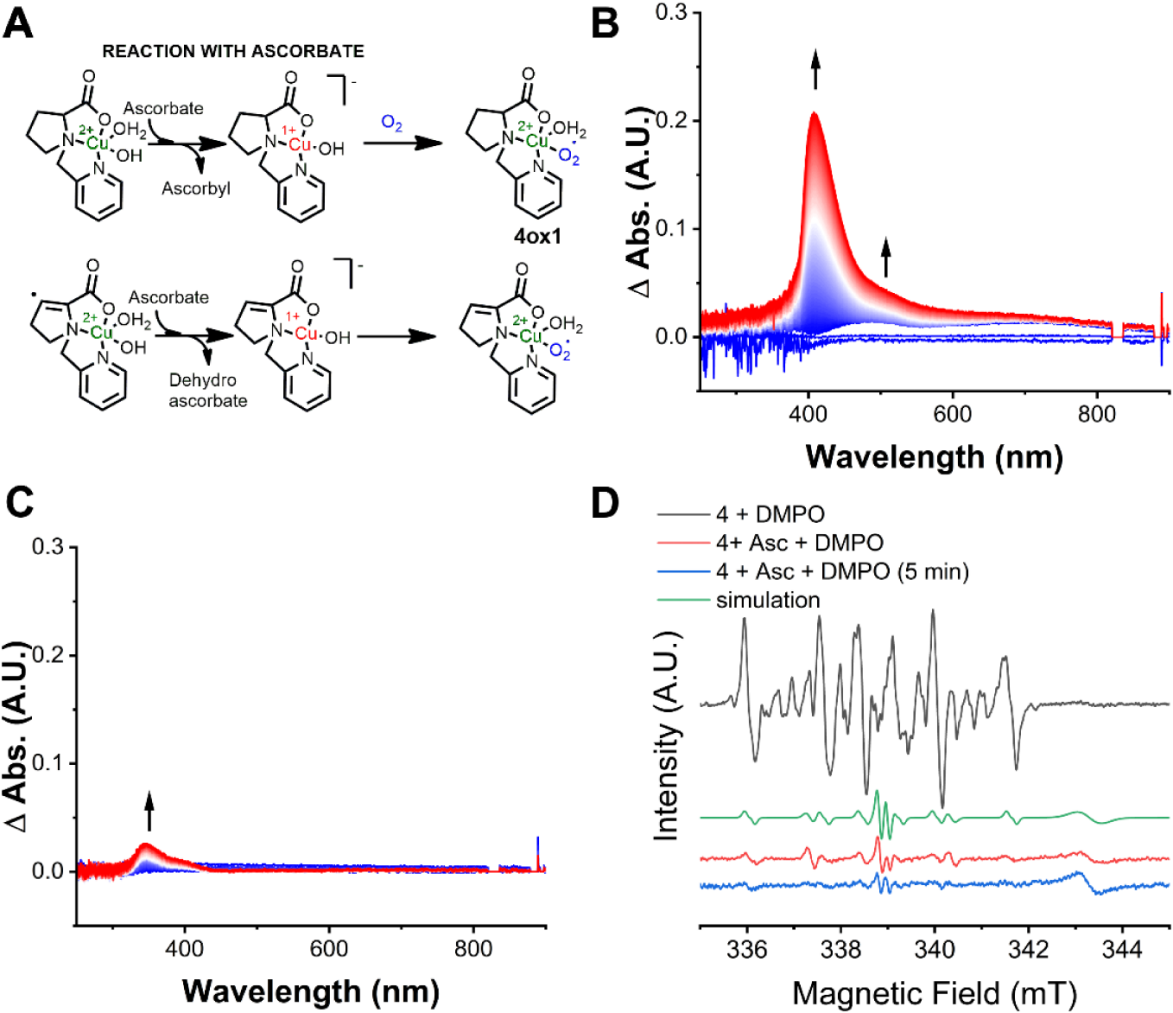
Reaction between complex **4** and dioxygen in the presence of sodium ascorbate as a reducing agent. A) Proposed scheme of the reaction, B) Electronic Spectra at the UV–vis region of **4** in aerobic medium in the presence of ascorbate, C) Electronic Spectra at the UV–vis region of **4** in anaerobic medium in the presence of ascorbate and D) EPR spectroscopy of DMPO-trapped radicals before and after the addition of ascorbate. It is shown that carbon-centered radicals are consumed by the addition of ascorbate. Experiments were performed in 100 mM carbonate buffer (pH 10.5) using 1 mM solution of complex **4** and 20 mM solution of sodium ascorbate in aerobic medium.

An EPR experiment of DMPO radical trapping of the reaction product between **4** and O_2_ was performed to see if any radical could be detected. In the presence of ascorbate, we observed a decrease in the concentration of carbon-centered radicals and the emergence of ascorbyl radicals (Figure 7D). This means that beyond copper reduction, ascorbate also serves as an electron donor to the ligand-centered radical, confirming the presence of radicals in solution. We were unable to detect superoxo species through this experiment, which could either indicate a transient, unstable intermediate as observed by the temperature dependent experiments, or that a peroxo complex is formed. To verify the latter, we decided to evaluate the formation of peroxide in the reaction between **4** and ascorbate in aerobic conditions. However, ascorbate is known to interfere with hydrogen peroxide detection assays, which could complicate the interpretation of the results.^72,73^ Tautomerization followed by oxidation in complex **4** is expected to generate reducing equivalents via the Cu­(II)/Cu­(I) cycle, potentially enabling H_2_O_2_ formation in the absence of an external reductant. Thus, to decouple the intrinsic reactivity of **4** from ascorbate-driven processes, we therefore examined whether complex **4** alone can reduce O_2_ to H_2_O_2_ under ascorbate-free conditions, similar to our previous report.^48^ Using a thiocyanide assay^74^ we observed that complex **4** exhibited the formation of H_2_O_2_, whereas complex **3** showed lower levels of hydrogen peroxide formation (Figure S18). As the generation of hydrogen peroxide requires two electrons, this experiment corroborates with the hypothesis of generation of a transient Cu­(I)-ligand radical (Scheme 1), where both the metal center and the ligand donate one electron each. Both redox centers in the Cu­(I)-ligand radical tautomer can each provide one electron to O_2_ to generate H_2_O_2_ (*O*_2_(*g*) + 2H^+^ + 2e^–^ → H_2_O_2_ at pH 7 is +281 mV), as the O_2_ redox potential decreases with the increase in pH.^75^ This experiment is in agreement with the observed O_2_ activation in the presence of reducing agents by PMOs such as LPMO,^19,75–77^ and points out to a possible monooxygenase-like activity of complex **4** in respect to complex **1–3**. It also raises the question whether the radicals formed in LPMO^20,23^ might have another role in O_2_ activation catalysis.

Once LPMOs encounter H_2_O_2_, the reaction proceeds rapidly.^78,79^ Given that complex **4** generates hydrogen peroxide, we next examined its reactivity toward H_2_O_2_. A computational study has shown that H_2_O_2_ does not readily dissociate from the Cu­(II) state of LPMOs.^77^ Therefore, to probe potential intermediate formation under these conditions, these experiments were conducted in the absence of reducing agents. Complexes **1–3** were also evaluated, as they might still mimic peroxygenase-like activity despite lacking the ability to produce H_2_O_2_ from O_2_. Spectroscopic monitoring revealed that their reaction with hydrogen peroxide did not induce significant UV–vis changes, apart from a slight decrease in the d–d transition band (Figure S19). In contrast, complex **4** displayed pronounced spectral changes (Figure 8). At 5 °C, the reaction led to the appearance of a new absorption band at 400 nm accompanied by a decrease in the d–d transition at 740 nm, consistent with the formation of a reaction intermediate (**4ox2**) (Figure 8A and B). These spectroscopical changes occurred with a rate of 0.45 s^–1^ (Figure S20), and although the d–d band has a significant decrease in intensity, it does not completely decay, exhibiting a weak peak centered at 683 nm. The emergence of bands at the 300–400 nm region has already been observed in the literature and is assigned to the formation of peroxo complexes.^37,80^ For instance, dinuclear μ-1-2 peroxo copper complexes exhibit absorption bands at this same region,^71^ which could indicate the nature of **4ox2** as a dimeric peroxo intermediate, which should be EPR silent,^81^ as observed by the decrease of EPR active species upon H_2_O_2_ addition to a solution of **4** (Figure 8C). This assignment aligns with known examples of strongly antiferromagnetically coupled Cu­(II) dimers.^82,83^ Moreover, although superoxo species have also been noticed to present bands in the same region,^84^ the redox potential of H_2_O_2_ does not indicate a spontaneous reduction of the complexes by peroxide (H_2_O_2_ → H^+^ + e^–^ + O_2_^•–^ at pH 10.5 is ≅+700 mV vs Ag/AgCl 3.5M). Therefore, we assign the spectroscopic features of **4ox2** to the formation of a copper-peroxo complex. Upon increasing the temperature from 5 °C to 37 °C, a decrease in the 400 nm band and a corresponding increase in the d–d transition band were observed (Figure 8D), indicating an instability of the intermediate. Noting that peroxo complexes are unstable at high temperatures,^85^ we performed an HRMS experiment of the frozen solution of **4ox2** (Figure S21). Two *m*/*z* peaks can be highlighted here: the first peak at *m*/*z* 630.97997, consistent with the formula C_22_H_26_Cu_3_N_4_O_6_ (calc: 630.97403), could relate to a dimeric peroxo complex of **4** (**4ox2**, Figure 8A), whereas the second one with *m*/*z* 593.04989, is consistent with the C_22_H_28_Cu_2_N_4_NaO_6_ composition (calc.: 593.04997), a dimeric hydroxo form. These observations point to the possibility of formation of dimeric species, in agreement with the EPR silent spectrum and UV–vis spectroscopy. Moreover, calculation of the relative free Gibbs energy of the reaction indicates that the dimeric peroxo species is spontaneously formed with a free Gibbs energy of −80 kJmol^–1^ relative to the starting complex. The dimeric hydroxo species is formed by passing a 30 kJmol^–1^ transition state, giving an overall exergonic reaction with ΔG of −120 kJmol^–1^.

**Figure 8:**
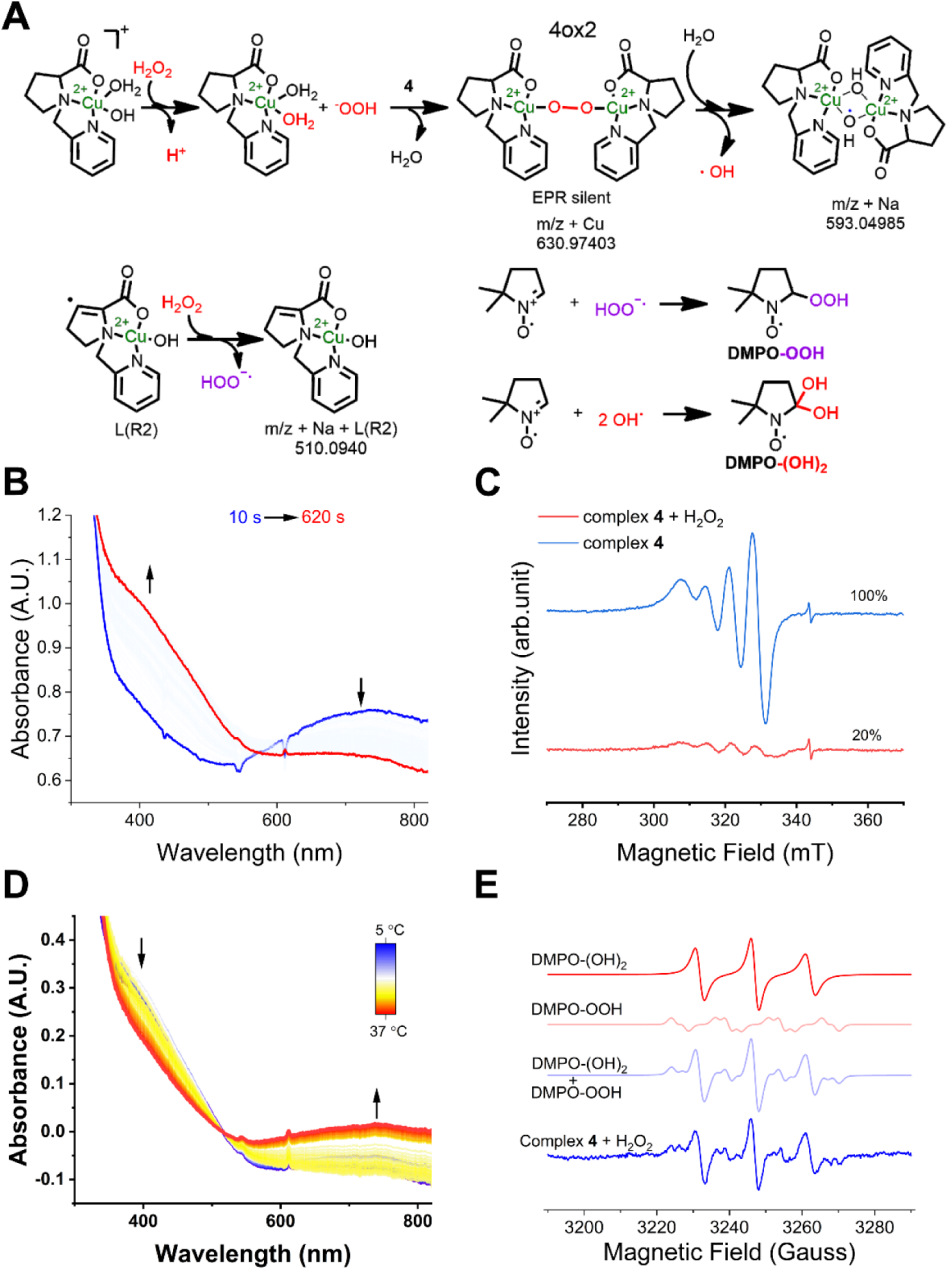
Reaction of complex **4** with hydrogen peroxide. A) Scheme of the reactions between **4** and H_2_O_2_ and the formed radicals with DMPO. B) Electronic Spectroscopy at the UV–vis region of the reaction at 5 °C. C) EPR spectroscopy before and after the addition of peroxide. D) UV–vis spectra of the reaction after 620s and with increasing temperature from 5 to 37 °C and E) EPR of DMPO-trapped intermediates at 5 °C show a formation of peroxyl radical and DMPO-(OH)_2_. Experiments performed in 100 mM carbonate buffer (pH 10.5) using 1 mM solution of complex **4** and 20 mM solution of H_2_O_2_.

Beyond dimer formation, DMPO spin-trapping experiments revealed the presence of hydroxyl^86^ and superoxyl radicals in solution (Figure 8E). Reactive •OH radicals can arise through several pathways, including the Haber–Weiss reaction, which involves the interaction between O_2_•^–^ and H_2_O_2_.^87,88^ In the case of LPMOs, hydroxyl radicals have been proposed to form via homolytic O–O bond cleavage, potentially through a Fenton-like mechanism, as suggested by Bissaro and Eijsink.^29^ A similar mechanism might be operative in our system. In this reaction none of the ligand centered radicals were observed, indicative of ligand oxidation. High-resolution mass spectrometry (HRMS) supports this, showcasing species with an additional oxygen atom consistent with oxidative modification of the ligand. In LPMOs and other metalloenzymes, 2-oxo-histidines have been identified as markers of oxidative damage.^89–91^ Methylation of the active site histidine and substrate presence increases the reaction barrier and decreases the oxidative damage. Moreover, the formation of a Cu­(II)-tyrosyl state^92^ also increases the energetic barriers in respect to the [Cu–O]^+^ intermediate. Thus, radicals in the active site help protect against oxidative damage caused by excess peroxide in the absence of substrate and, similarly, the radicals in our complexes might also confer such protection. To assess whether the radicals could exert a protective effect, we recorded the cyclic voltammogram of complex **4­(Ar)** and compared it with that of complex **4** (Figure 6). The CV of complex **4­(Ar)** also displays a reduction wave without a corresponding oxidation wave, supporting electron transfer from the electrochemically generated Cu­(I) species to the carboxylate moiety of the ligand, leading to the formation of Cu­(II) and a COO^2•^–^^ species (Figure S22). Interestingly, because complex **4­(Ar)** lacks a ligand-centered radical that could recombine with the COO_2_•^–^ species, the corresponding oxidation wave is clearly visible in the first cycle, together with additional oxidation events. The presence of multiple oxidation processes in the CV of complex **4­(Ar)** indicates that this complex is readily oxidized into different species, reflecting its lower stability. Indeed, after four cycles, the reduction wave shifts by approximately 100 mV toward less negative potentials, suggesting the formation of new species in solution.

Given the intriguing reactivity of complex **4** with both O_2_ and H_2_O_2_, we proceeded to investigate its potential to mimic LPMO-like activity using both cosubstrates.

### 2.3 Complex 4 Mimics the Use of Both Co-Substrates

The use of peroxide in an LPMO assay is common in the literature, supporting a peroxygenase activity (R-H + H_2_O_2_ = R–OH + H_2_O) aiding the study of the peroxygenase activity of mimics and the enzyme itself.^9,79^ Our initial tests were based on the oxidative cleavage of 4-nitrophenyl-β-d-glucopyranoside in the presence of complexes **1–4**, hydrogen peroxide and 100 mM carbonate buffer (pH 10.5). Controls without one of the components were performed and no significant substrate decomposition was observed (Figure S23). Control reactions using the same conditions from catalysis, but in the presence of copper sulfate or copper chloride (Figures 9B, S23 and S24) were also performed. The kinetic parameters *k*_obs_ were calculated in the best conditions of catalysis (0.1 mM concentration of complex, 20 mM substrate and 20 mM hydrogen peroxide). All complexes were fit to a double exponential fit, in which a fast process (Figure S25, *k*_obs_ of ≅0.06 s^–1^) is followed by a second, slower one. Interestingly, complexes **1–3** have a much slower process (*k*_obs_ of ≅10 × 10^–9^ s^–1^) than complex **4** (*k*_obs_ of ≅0.01 s^–1^). Initial assessments indicated that **1** and **2** did not exhibit high catalytic activities (Figures 9B, S24), with activities almost comparable to the control experiments. On the other hand, complexes **3** and **4** (Figures 9, S24) exhibited a higher activity for the oxidative cleavage of the substrate.

**Figure 9:**
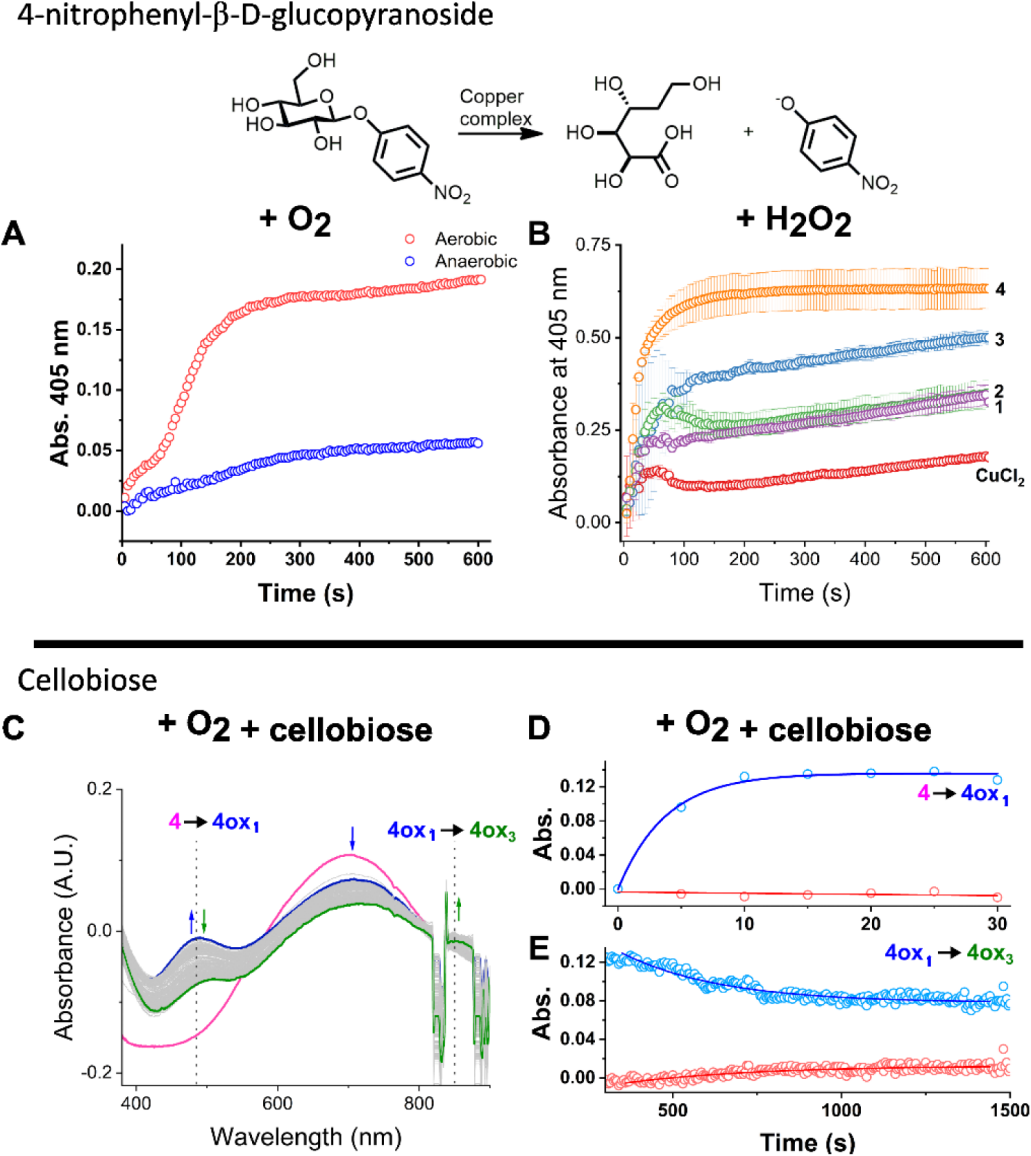
Catalysis of oxidative cleavage by copper complexes. A) Reaction of the cleavage of 20 mM 4-nitrophenyl-β-d-glucopyranoside using 1 mM of complex **4** and sodium ascorbate (20 mM) in the presence and absence of oxygen. B) UV–vis monitoring of the reaction of 4-nitrophenyl-β-d-glucopyranoside oxidation in the presence of hydrogen peroxide. All reactions were performed with 0.1 mM of the copper compound in a 100 mM carbonate solution (pH = 10.5), 20 mM H_2_O_2_ and 20 mM substrate. The cuvette containing all components except for the copper compound was used as a blank. The reaction kinetics were measured by the increase of *p*-nitrophenolate product absorbance at 245 nm over time after the addition of each copper compound: (red) control with CuCl_2_, (green) reaction with **1**, (purple) reaction with **2**, (blue), reaction with **3**, and (orange) reaction using **4**. C) Monitoring of the formation of **4ox1** and its consumption after the addition of cellobiose. D) The absorbance of bands at 450 nm (**4ox1** formation) and 850 nm (**4ox3** formation) were monitored, indicating that **4ox1** is formed in the presence of O_2_. E) The absorbance of bands at 450 nm (**4ox1**) and 850 nm (**4ox3** formation) were monitored, indicating that **4ox1** is consumed after the addition of cellobiose, whereas **4ox3** formed.

After leaving the reaction for 24 h at 37 °C, the same trend was observed, in which **1** and **2** presented the lowest conversion values (810 ± 46 μM (4% yield) and 729 ± 15 μM (3.6% yield), respectively), close to the control reaction with a simple copper salt (683 ± 49 μM, 3.4% yield) (Figure S26). Complex **3** did not exhibit a great improvement in comparison to the other compounds, degrading 832 ± 30 mM (4.2%) of the substrate over the 24 h period (Figure S26). Interestingly, over the same period, complex **4** almost doubled the conversion obtained for complex **3**, exhibiting values of 1.53 ± 0.08 mM (7.7%) of degradation (Figure S26). These values are comparable to the ones obtained by a complex with a histidine brace motif, in which ≅0.8 mM of conversion was obtained after 24 h under similar conditions.^37^

Considering that complex **4** contains a higher concentration of radical species than complex **3**, these radicals could play a role in LPMO mimicry, either by participating in the catalytic cycle or providing a protective effect. To investigate this hypothesis, we compared the catalytic performance of **4** and **4­(Ar)**, which differ primarily in their carbon-centered radical content (15% for **4** and 1% for **4­(Ar)**). Interestingly, complex **4­(Ar)** exhibited an activity about 15% faster than **4** (Figure S27). This implies that the higher the concentration of radicals, the slower the peroxygenase-like catalysis, in agreement with LPMO, as tyrosyl radicals were shown to render inactive enzymes.^93^ However, this trend does not hold when different complexes are compared. For example, complex **3** is slower than complex **4** despite exhibiting a lower radical concentration. Thus, the difference in activity between **3** and **4** likely arises from other factors, such as steric hindrance or reactivity toward hydrogen peroxide. Complexes **1–3** appear to be more sterically hindered, whereas complex **4**, with a more exposed active site, shows the highest peroxidase activity of all the complexes tested. This interpretation could also account for the subtle difference between complexes **1–2** and **3**. For example, while **1** and **2** possess a flexible moiety attached to the triazole ring that may impede substrate access to the copper center, complex **3** contains a rigid phenyl substituent. The rigidity of this group likely reduces its steric effect compared to the flexible substituents but still contributes to lower activity relative to **4**. Moreover, since dimeric species were detected during the reaction between complex **4** and hydrogen peroxide, the impact of steric hindrance on catalysis becomes more evident, as less hindered complexes are more prone to form and stabilize the dimeric intermediate.

This reaction is expected to exhibit strong pH dependence if deprotonation of hydrogen peroxide constitutes a key step in the mechanism (Figure 8A). Indeed, no conversion was observed at pH 7.5, whereas increasing the pH to 8.5 enabled the reaction. Notably, the reaction rate at pH 8.5 (*k*_obs_= 3 × 10^–4^ s^–1^) was approximately ten times slower than at pH 10.5 (Figure S28), confirming that deprotonation by the hydroxo moiety is essential for reactivity. The increase in activity from pH 7.5 to pH 8.5 agrees with the p*K*_a_ of 7.9 observed for complex **4**, as in higher pHs there is a higher concentration of Cu–OH species. In fact, this species was observed in the HRMS analysis of the reaction between **4** and H_2_O_2_ (Figure S21), where a peak at *m*/*z* 308.01943 can correspond to C_11_H_14_CuN_2_NaO_3_ (calc. *m*/*z* 308.01981), indicating its importance in the reaction mechanism. Consistent with this, the determined kinetic isotope effect (KIE) of (k_H_/k_D_) ≅1.7 (Figure S29) suggests that the rate-determining step may involve formation of a deprotonated peroxide species. The relatively modest KIE aligns with a proton transfer involving a weak O–H bond with minimal covalent character,^94^ such as the ones from H_2_O_2_.

Considering that LPMO activity with O_2_ as a substrate is due to the formation of H_2_O_2_, we picked the two best mimics with peroxygenase-like activity (complexes **3** and **4**) and evaluated their ability to use oxygen as a cosubstrate in the presence of ascorbate. These reactions were performed in aerobic conditions and the oxidative decomposition of 4-nitrophenyl-β-d-glucopyranoside was assessed.^95^ Both complexes were able to decompose the substrate in the presence of ascorbate and oxygen (Figure S30). In a closed vial, after 24 h of reaction, **4** was able to degrade 147 ± 14 μM of the substrate (0.8% yield), reaching the saturation level expected based on the limited dioxygen solubility in aqueous solutions (150 μM). By comparison, the control reaction with copper sulfate and the assay in the presence of complex **3** exhibited lower levels of decomposition (60 ± 10 μM (0.3%) and 111 ± 12 mM (0.6%), respectively, Figure S31). The higher reactivity of complex **4** toward the use of oxygen as a substrate was expected as the redox potential of dioxygen (O_2_ + e^–^ → O_2_^•–^) is −365 mV (vs Ag/AgCl 3.5 M KCl),^8^ and results in a positive free Gibbs energy whereas reduced complex **3** (120 mV vs Ag/AgCl 3.5 M KCl) was not expected to have a spontaneous redox reaction with dioxygen. Complex **3** was considered inactive as a monooxygenase, as its catalytic yields were comparable to the control. Confirmation that the reaction was dependent on oxygen was obtained by the comparison of a reaction in anaerobic conditions using **4** as a catalyst. As expected, low levels of *p*-nitrophenolate were detected (Figure 9B), corroborating the idea that oxygen is activated to oxidize the substrate.

To further assess the substrate scope of complex **4** we extended our studies from 4-nitrophenyl-β-d-glucopyranoside to cellobiose. As cellobiose does not have a UV–vis spectral feature that allows the monitoring of the reaction, we formed intermediate **4ox1** and monitored its spectral features after adding a cellobiose solution. As can be observed in Figure 9C, **4ox1** is consumed by such glycosidic substrates. For instance, when cellobiose solution was added (10 mM, final concentration), a new species was formed (**4ox3**). The species **4ox3** has a band at 850 nm and by monitoring the emergence of the bands at 450 nm (**4ox1**) and 850 nm (**4ox3**) (Figure 9D) we verified that **4ox1** is formed with a rate of 3 s^–1^ and is consumed at a 3 × 10^–3^ s^–1^ rate when cellobiose is added (Figure 9E). HPLC-MS of this reaction reveals the conversion of 3.4% of cellobiose into glucose after 24 h of reaction (Figure S32). This conversion efficiency is consistent with the solubility limit of oxygen in aqueous solution of a closed vial and corroborates the activation of dioxygen for the consumption of this oligosaccharide. In comparison, intermediate **4ox2** reacts with cellobiose at a rate roughly 6-fold higher than that observed for **4ox1** (Figure S33). This difference in reactivity supports the conclusion that the two species possess distinct chemical characteristics, in agreement with the EPR and UV–vis analyses.

Considering that cellobiose is the disaccharide of cellulose, we decided to evaluate the ability of complex **4** to degrade filter paper as a substrate in the presence of different additives (H_2_O_2_ or ascorbate). The activity was evaluated by a colorimetric method using DNS methodology to verify the amount of reducing sugars formed in the reaction.^96^ Three assays were performed, one with **4** without any additive and two others using either ascorbate or H_2_O_2_ (Figure S33). These reactions were compared with blank experiments without catalyst. Interestingly, complex **4** has a hydrolytic activity at pH 10.5, producing 1.61 μmol (±0.05) of reducing sugars per minute per milliliter in the absence of any additive. Upon addition of ascorbate the produced sugars increased by a factor of 2 (3.48 ± 0.10 μmol), whereas the addition of H_2_O_2_ did not improve the degradation (1.39 ± 0.02 μmol of reducing sugars). Although the degradation of 4-nitrophenyl-β-d-glucopyranoside yielded higher conversions when peroxide was used as a cosubstrate, adding H_2_O_2_ to cellulose degradation proved detrimental. HRMS analysis of the reaction between complex **4** and either peroxide or O_2_ (Figures S34 and S35) revealed that peroxide progressively degrades the catalyst, whereas O_2_/ascorbate does not. This suggests that while radicals in the complex can protect it from oxidative damage to some extent, high peroxide concentrations cause irreversible oxidation, as peroxide at concentrations equimolar to the substrate produces a large excess of hydroxyl radicals, which in turn can accelerate the consumption of the active complex.

## 3 Conclusions

This study demonstrates that ligand-centered radicals can play a crucial role in the reactivity and stability of copper complexes designed as LPMO biomimetic models. For example, the N,N,O,O-coordinated copper complex **4** exhibited a high proportion of ligand-centered radicals, that enabled its reaction with dioxygen, forming H_2_O_2_ in the absence of reducing agents. The detection of two distinct carbon-based radicals suggests a sequential radical transformation reminiscent of the hole-hopping mechanism proposed for LPMOs. Importantly, these radicals appear to act as protective intermediates, preventing overoxidation of the complex in the presence of hydrogen peroxide. Overall, these results establish that controlled formation of ligand-centered radicals can emulate the redox buffering and electron-transfer functions observed in copper-dependent oxidative enzymes. This insight provides a valuable framework for the rational design of next-generation copper catalysts capable of combining reactivity with self-protection mechanisms.

## 4 Experimental Part

All chemicals were used as purchased from Sigma-Aldrich and were used without previous purification unless otherwise indicated.

### 4.1 Synthesis of Complex 4

To 8 mL of methanol were added 154 mg of Cu­(ClO_4_)_2_.6H_2_O (0.414 mmol). After 5 min, a methanolic solution of 91 mg of ligand 1-(2-pyridinylmethyl)-l-proline methyl ester (0.414 mmol) was added dropwise. The reaction proceeded at 40 °C for 4 h. After that, the reaction mixture was cooled to room temperature until blue crystals formed. A mixture of methanol/diethyl ether was added to the supernatant, which allowed obtaining more blue crystals. The crystals were washed with diethyl ether resulting in 112 mg (70% yield). Conductivity (μS cm^–1^): 132.0 s ± 0.5 in methanol. HRMS (*m*/*z*): isotopic cluster found 452.5138 ([**4**]_3_Cu]^2+^) (calc 451.5158). FTIR in KBr (cm^–1^): 3477; 2964, 2947; 1651, 1614; 1566; 1483, 1446, 1421, 1357, 1300; 1109, 1093; 765, 627. Anal. Calcd for C_11_H_15_ClCuN_2_O_7_: C 34.21; H 3.91; N 7.25. Found: C: 34.08; H: 4.22; N 7.36.

Note: the synthesis, storage and handling of complex **4** was performed under aerobic conditions, unless otherwise stated.

### 4.2 Kinetics of Peroxygenase Activity

Activity assays were run by monitoring the increase in 405 nm absorbance on an Ocean Optics QE65000 spectrometer with a xenon lamp source, following the oxidation of 4-nitrophenyl-β-d-glucopyranoside to 4-nitrophenolate every 5 s. To run the assays, 100 mM sodium carbonate pH 10.5 buffer, 4-nitrophenyl-β-d-glucopyranoside (20 mM), and copper catalyst (1 mM) were added into a cuvette and allowed to equilibrate at 37 °C in an Ocean Optics QPOD temperature-controlled cuvette stage for 5 min. The reaction initiated with the addition of a hydrogen peroxide solution (final concentration 20 mM). All experiments were performed in triplicate. For the determination of the turnover rate (s^–1^) either a double exponential or single exponential was fit to the data. Yields were determined from the concentration of 4-nitrophenolate formed, calculated using the absorbance at 405 nm (ε = 18,000 M^–1^ cm^–1^). The concentration of 4-nitrophenolate obtained at the specified time point was divided by the initial substrate concentration (20 mM) and multiplied by 100 to give the reaction yield (%).

### 4.3 Kinetics of Monooxygenase Activity

Activity assays were run by monitoring the increase in 405 nm absorbance on an Ocean Optics QE65000 spectrometer with a xenon lamp source, following the oxidation of 4-nitrophenyl-β-d-glucopyranoside to 4-nitrophenolate every 5 s. To run the assays, a 100 mM sodium carbonate pH 10.5 buffer, 4-nitrophenyl-β-d-glucopyranoside (20 mM), and copper catalyst (1 mM) were added into a cuvette and allowed to equilibrate at 37 °C in an Ocean Optics QPOD temperature-controlled cuvette stage for 5 min. The reaction initiated with the addition of a sodium ascorbate solution (final concentration 20 mM). All experiments were performed in triplicate. For the determination of the turnover rate (s^–1^) either a double exponential or single exponential was fit to the data. Control data in anaerobic conditions were performed by purging the initial solutions with N_2_ for 20 min and preparing the sample in an anaerobic box ([O_2_] < 10 ppm) using a cell capped with a screw-cap. Yields were determined from the concentration of 4-nitrophenolate formed, calculated using the absorbance at 405 nm (ε = 18,000 M^–1^ cm^–1^). The concentration of 4-nitrophenolate obtained at the specified time point was divided by the initial substrate concentration (20 mM) and multiplied by 100 to give the reaction yield (%).

### 4.4 Complex 4 Activity with Filter Paper as Substrates in the Presence and Absence of the Additives

Complex **4** activity assays against filter paper substrate were developed using the DNS method, estimating the concentration of reducing sugars released in the reactions. To run the assays, 62.4 μL of 50 mM bicarbonate buffer pH 10.5, filter paper (0.5 × 0.75 mm) and 31.2 μL of 5 mM complex 4 were incubated at 45 °C for 1 h. After this period, 188 μL of DNS reagent was added to the reaction, which was incubated at 100 °C for 15 min. Then, the solution was cooled in an ice bath for 5 min and finally 1250 μL of water was added. The absorbance reading at 540 nm of the solution was taken on a Thermo Scientific Biomate 160 spectrophotometer and the measurement obtained was compared with data from a glucose standard curve.

Monooxygenase and peroxidase activities of complex **4** against filter paper were determined using the same method, replacing the volume of bicarbonate buffer with solutions of ascorbic acid or hydrogen peroxide (additives) at a final concentration of 10 mM. A solution containing all reagents without complex **4** was used as a blank. All experiments were performed in triplicate.

### 4.5 Computational Protocol

A conformational search was carried out using the Conformer Rotamer Ensemble Sampling Tool (CREST) protocol,^97^ based on the semiempirical extended Tight-Binding approach GFN1-xTB.^98^ Conformers found within the energy window of 0–6 kcal mol^–1^ were optimized using the ORCA package^99^ at the density functional theory (DFT) level of theory with default convergence criteria employing Ahlrichs’ triple-ζ def2-TZVP basis set^100^ and PBE0 (default 25% exchange) exchange-correlation functional.^101^ The resolution of the identity approximation for Coulomb (RI-J) integrals in conjunction with matching auxiliary basis sets (def2/J option) was applied to speed up DFT calculations.^102^ The D3 London dispersion correction scheme was applied.^103^ In the postprocessing, we performed single point calculations at using the PBE0-DH exchange-correlation functional,^104^ and Ahlrichs’ triple-ζ def2-TZVPP4 basis set, with matching auxiliary basis sets (def2/J and def-SVP/C options) into extreme convergence SCF criteria.

Solvation effects were considered by the implicit solvation with methanol using the Conductor-like Polarizable Continuum Model (CPCM) for the DFT level,^105^ and the generalized born (GB) model with a solvent accessible surface area (SASA) termed as GBSA,10 as implemented for GFN1-xTB in CREST.

The CI-NEB calculations were performed in the ORCA package.^99^ In between the initial and final states calculated from the models, a global maximum was found along the reaction coordinate. To determine the barrier within the initial state and the final state, we used 12 images. The calculations were conducted under the same protocol used for the ground-state geometry optimization.

The topological analysis of the electron density can provide insights into the nature of the bonding in the system. The electronic densities of the systems were evaluated through the Quantum Theory of Atoms in Molecules (QTAIM) yielded the liquid Bader’s charges (Δq = Z – q_Bader_, where Z is the number of electrons nonpseudodized), the electronic density, ρ_BCP_(r_c_), and the Laplacian, ∇^2^ρ_BCP_(r_c_), of the Bond Critical Point (BCP), and other topological descriptors, were calculated at the atoms, using the CRITIC2 program.^106^

BCP descriptors, Bader and Mulliken charges, Macchi’s classification based on local (BCP) and integral properties, Bianchi classification based on local (BCP) and integral properties are found in the SI (Tables S4–S7).

## 5 Methods

### 5.1 Solid State FTIR

Fourier Transform Infrared (FTIR) spectra were obtained in KBr pellets using a Bomen-Michelson FT spectrometer model MB-102. All measurements were obtained in the interval of 400 and 4000 cm^–1^.

### 5.2 Electronic Spectroscopy in the UV–Vis Region

Electronic spectra were recorded in a HP–Hewlett-Packard 8452 A spectrophotometer. The samples were analyzed in solution using a quartz cell with 1 mL maximum volume and optical path of 1.0 cm. Values of molar absorptivity, ε, were calculated using the maximum absorbance value of the bands from the Lambert–Beer law (ε = A/bC), in which A = absorbance, b = optical path and C = concentration in mol L^–1^.

### 5.3 Electronic Paramagnetic Resonance (EPR)

Measurements were recorded at room temperature (296 K) and at liquid nitrogen temperature (77 K). For the measurements an EPR equipment model Varian E109, X band, using a rectangular cavity and modulation at 100 kHz. The parameters for the measurements were power of microwave (20 mW), modulation amplitude (0.4 mT peak to peak) with automatic gain for each sample, field scanning of 160 mT, 0.064s. Scanning of 3 min. To calibrate the magnetic field an EPR standard was employed (MgO:Cr­(III) g = 1.9797 crystal) and the resonance frequency was measured with a microwave frequency meter.

### 5.4 Microanalysis

All microanalyses were performed by the Analytical Central from the Department of Chemistry at UFSCar using an EAGER 200 CE equipment.

### 5.5 Electrochemical Measurements

The electrochemical measurements were performed using an EG&G potentiostat Princeton Applied Research Model 273A/A conventional glass cell with three electrodes was used. The electrodes used were vitreous carbon (0,071 cm^2^), platin and Ag_(s)_/AgCl_(s)_|KCl^–^ (3.5 M) as working, auxiliary and reference electrodes, respectively. All measurements were performed in methanol containing tetrabutylammonium perchlorate 0.1 M as an electrolyte. To remove dissolved oxygen, argon was purged in the cell for 15 min prior to each scan. The working electrode was polished with alumina 0.05 μm before the experiments, followed by washing with water. The auxiliary and reference electrodes were washed with methanol prior to their addition to the cell.

### 5.6 Mass Spectrometry

HRMS of complex 4 at high concentrations and with H2O2 were performed at Emory University with Thermo Exactive Plus using methods equivalent to APCI or ESI.

HRMS of complex 4 at low concentrations and its reaction with H2O2 and ascorbate (low concentrations) were performed at the Chemistry Department of UFSCar using LC-QqTOF system from BRUKER DALTONICS.

LC-MS analysis was performed at Emory University using Thermo LTQ-FTMS and using a C18 column starting at 100% Water, then 1% formic 15 min gradient to 100% Acetonitrile after holding for 5 min at 100% Water.

### 5.7 Single Crystal X-ray Diffraction

The measurements of single crystals by X-ray diffraction were performed on Rigaku XtaLAB mini II diffractometer with graphite monochromated Mo Kα radiation (λ = 0.71073 Å). Cell refinements were carried out using the CrysAlisPro software, and the structures were obtained by the intrinsic phasing method using the SHELXT program. The Gaussian method was used for the absorption corrections. Table and structure representations were generated by OLEX2 and MERCURY, respectively. The Table S1 summarizes the structural and crystallographic parameters. Crystal Structures (S35–S37) and tables with bond angles and distances (Tables S8–S33) can be found in the SI.

### 5.8 X-ray Emission Spectroscopy (XES)

The samples for XES were prepared by grinding the crystalline forms of compounds 1–4 and packing the powder in an aluminum cell. The measurements were measured at the PINK beamline at BESSY II Synchrotron (Berlin) as described by Gerz et al.^107^ The energy calibration was performed using Zn Kα and Cu Kβ lines measured from metallic foils, according to the procedure described elsewhere.^108^

## Supplementary Material



## Data Availability

The data that supports the findings of this study are available in the manuscript and SI. Other data not available in the manuscript can be obtained upon contact with the corresponding author, CGCMN, upon reasonable request.

## Notes

Figure S1: FTIR of **4**; Figure S2: UV–vis and EPR of **4** at RT; Figure S3: EPR of **4** at 30 K; Figure S4: molar absorptivity experiment; Figure S5: RT EPR of complex **4** before and after DMPO addition; Figure S6: DFT calculation of the HOMO–1 of C-centered DMPO spin adduct; Figure S7: XES of 1–3; Figure S8: RT EPR of complex **3**, **3-Zn**, and **3-Ligand**; Figure S9: XPS of **3** and **4** (Cu 2p); Figure S10: HOMO and LUMO of **3**; Figure S11: HOMO and LUMO of **3-Zn**; Figure S12: HOMO and LUMO of **4**; Table S2: Wigberg/Mayer bond orders; Table S3: HOMO and LUMO orbital contributions; Figure S13: HRMS of 4; Figure S14: electronic spectroscopy at the UV-Vis region of complex 4 and complex 4­(Ar); Figure S51: EPR of DMPO-trapped analogous complexes to **4**; Figure S16: EPR of DMPO-trapped analogous complexes to **4-Zn**; Figure S17: UV–vis and EPR of the reaction of **4** with ascorbate; Figure S18: UV–vis of **4ox1**; Figure S19: H_2_O_2_ detection; Figure S20: UV–vis and EPR of the reaction of **1–3** with H_2_O_2_; Figure S21: kinetics of **4ox2** formation; Figure S22: HRMS of **4** in the presence of peroxide; Figure S23: controls of oxidative reactions; Figure S24: CV of complex 4­(Ar); Figure S25: kinetics at different substrate concentrations; Figure S26: UV–vis spectra of peroxidase activity of **1–4**; Figure S27: fits of 4-nitrophenolate production; Figure S28: 24h concentration of 4-nitrophenolate of reactions with H_2_O_2_; Figure S29: influence of the reaction pH on the reaction kinetics; Figure S30: kinetic Isotope Effect of the reaction of 4-nitrophenyl-β-d-glucopyranoside degradation; Figure S31: kinetics at different ascorbate concentrations; Figure S32: 24h concentration of 4-nitrophenolate of reactions with ascorbate; Figure S33: HPLC-MS; Figure S34: Consumption of 4ox2 by cellobiose; Figure S35: DNS quantification of cellulose degradation; Figure S36: HRMS of reaction between **4** and ascorbate; Figure S37: HRMS of the reaction between complex **4** and peroxide; Tables S3–S5: BCP descriptors and Bader and Mulliken charges; Figure S38: X-ray structure of **4** crystallized in ACN; Table S6–S13: X-ray crystal parameters for **4** crystallized in acetonitrile; Figure S39: X-ray structure of **4** crystallized in MeOH; Table S13–S22: X-ray crystal parameters for **4** crystallized in methanol; Figure S40: X-ray structure of **4** crystallized in water; Tables S23–S31; X-ray crystal parameters for **4** crystallized in water (PDF)
