# Supplementary material for: Ligand Radicals Tune LPMO Activity in Model Complex
Source: Inorg Chem. 2026 Feb 26;65(10):5357–73. doi: 10.1021/acs.inorgchem.5c05285 (PMC12997159; doi:10.1021/acs.inorgchem.5c05285)
Supplement: Supplementary file 1 [file ic5c05285_si_001.pdf]

**Supporting Information**

# Ligand Radicals Tune LPMO Activity in Model Complex

*Caterina G. C. Marques Netto [a,b] *, Ritika Pandey [a], Caio Bezerra de Castro [b], Larissa Moreno[b], Millena P. Ferreira[b], Lullie Gomes Rodrigues [b], Walber Gonçalves Guimaraes[b], João Honorato de Araujo-Neto[c], Gabrielle Conciani [b], R. Brian Dyer [a], Sergio A. V. Jannuzzi [d], André F. de Moura [b], Dulce H. Ferreira de Souza[b], Otaciro R. Nascimento [e]*

a. Department of Chemistry, Emory University, 1515 Dickey Drive, Atlanta-GA, 30322, United States of America

b. Departamento de Química, Universidade Federal de São Carlos (UFSCar), Rod. Washinton Luiz, s/n km 235, CEP 13565905, São Carlos-SP, Brazil

c. Instituto de Química, Departamento de Química Fundamental, Universidade de São Paulo (USP), Av. Prof. Dr. Lineu Prestes, 748, CEP 05513-970 São Paulo, SP, Brazil

d. Max Planck Institute for Chemical Energy Conversion, Stiftstr. 34–36, 45470 Mülheim an der Ruhr, Germany

e. Instituto de Fisica de São Carlos, Universidade de São Paulo (USP), Av. Joao Dagnone, 1100, CEP 13563-120, Sao Carlos, SP-Brazil

*caterina@ufscar.br

# Contents

Table S1. ........................................................................6
Spectra and Results ........................................................................6
Figure S1. ........................................................................6
Figure S2. ........................................................................7
Figure S3. ........................................................................7
Figure S4. ........................................................................8
Figure S5. ........................................................................8
Figure S6. ........................................................................9
Figure S7. ........................................................................10
Figure S8. ........................................................................11
Figure S9. ........................................................................11
Figure S10........................................................................11
Figure S11. ........................................................................12
Table S2 ........................................................................12
Table S3. ........................................................................12
Figure S12. ........................................................................13
Figure S13. ........................................................................14
Figure S14. ........................................................................15
Figure S15. ........................................................................15
Figure S16. ........................................................................16
Figure S17........................................................................16
Figure S18........................................................................17
Figure S19........................................................................18
Figure S20........................................................................18
Figure S21........................................................................18
Figure S22........................................................................19
Figure S23........................................................................20
Figure S24........................................................................21
Figure S25........................................................................22
Figure S26........................................................................23
Figure S27........................................................................24

Figure S28 .......... 24
Figure S29 .......... 25
Figure S30 .......... 25
Figure S31 .......... 26
Figure S32 .......... 26
Figure S33 .......... 27
Figure S34 .......... 28
Figure S35 .......... 28
Figure S36 .......... 29
Figure S37 .......... 30
Table S4 .......... 31
Table S5 .......... 31
Table S6 .......... 31
Table S7 .......... 31
Figure S38 .......... 32
Table S8 .......... 32
Table S9 .......... 33
Table S10 .......... 34
Table S11 .......... 35
Table S12 .......... 35
Table S11 .......... 36
Table S13 .......... 37
Table S14 .......... 38
**CuL-ACN** .......... 38
Table S15 .......... 38
Figure S39 .......... 39
Table S16 .......... 39
Table S17 .......... 40
Table S18 .......... 41
Table S19 .......... 42
Table S20 .......... 43
Table S21 .......... 43
Table S22 .......... 44

Table S23........................................................................................................45
**CuL-MeOH**........................................................................................................45
Table S24........................................................................................................45
Figure S40........................................................................................................46
X-Ray Crystal Parameters for 4 crystallized in water........................................................................................................46
Table S25........................................................................................................46
Table S26........................................................................................................48
Table S27........................................................................................................49
Table S28........................................................................................................50
Table S29........................................................................................................51
Table S30........................................................................................................52
Table S31........................................................................................................53
Table S32........................................................................................................54
CuL-water........................................................................................................54
Table S33........................................................................................................54

| Identification | CuL-water | CuL-MeOH | CuL-ACN |
|---|---|---|---|
| CCDC code | 2337595 | 2337594 | 2337593 |
| Empirical formula | $C_{11}H_{15}ClCuN_2O_7$ | $C_{12}H_{17}ClCuN_2O_7$ | $C_{13}H_{16}ClCuN_3O_6$ |
| Formula weight | 386.24 | 400.26 | 409.28 |
| Temperature/K | 293(2) | 293(2) | 293(2) |
| Crystal system | orthorhombic | orthorhombic | monoclinic |
| Space group | $P2_12_12_1$ | $P2_12_12_1$ | $P2_1$ |
| a/Å | 7.1965(8) | 6.9604(8) | 11.4151(8) |
| b/Å | 10.9293(13) | 12.3739(13) | 6.9420(3) |
| c/Å | 18.810(2) | 18.413(3) | 12.0661(9) |
| α/° | 90 | 90 | 90 |
| β/° | 90 | 90 | 118.066(9) |
| γ/° | 90 | 90 | 90 |
| Volume/$Å^3$ | 1479.5(3) | 1585.8(3) | 843.72(11) |
| Z | 4 | 4 | 2 |
| $\rho_{calc}g/cm^3$ | 1.734 | 1.676 | 1.611 |
| $\mu/mm^{-1}$ | 1.693 | 1.582 | 1.487 |
| F(000) | 788.0 | 820.0 | 418.0 |
| Crystal size/$mm^3$ | 0.239 × 0.116 × 0.066 | 0.373 × 0.147 × 0.06 | 0.42 × 0.314 × 0.207 |
| Radiation | Mo Kα (λ = 0.71073) | Mo Kα (λ = 0.71073) | Mo Kα (λ = 0.71073) |
| 2Θ range for data collection/° | 5.714 to 51.49 | 5.516 to 51.472 | 6.75 to 68.654 |
| Index ranges | -8 ≤ h ≤ 8, -13 ≤ k ≤ 12, -22 ≤ l ≤ 22 | -8 ≤ h ≤ 8, -15 ≤ k ≤ 15, -18 ≤ l ≤ 22 | -18 ≤ h ≤ 17, -10 ≤ k ≤ 10, -16 ≤ l ≤ 18 |
| Reflections collected | 9103 | 14857 | 11268 |
| Independent reflections | 2813 [$R_{int}$ = 0.0333, $R_{sigma}$ = 0.0306] | 3037 [$R_{int}$ = 0.0733, $R_{sigma}$ = 0.0531] | 6022 [$R_{int}$ = 0.0223, $R_{sigma}$ = 0.0375] |
| Data/restraints/parameters | 2813/85/214 | 3037/101/248 | 6022/81/227 |
| Goodness-of-fit on $F^2$ | 1.104 | 1.092 | 1.084 |
| Final R indexes [I>=2σ (I)] | $R_1$ = 0.0331, $wR_2$ = 0.0811 | $R_1$ = 0.0765, $wR_2$ = 0.2005 | $R_1$ = 0.0388, $wR_2$ = 0.0939 |
| Final R indexes [all data] | $R_1$ = 0.0386, $wR_2$ = 0.0862 | $R_1$ = 0.0888, $wR_2$ = 0.2185 | $R_1$ = 0.0518, $wR_2$ = 0.1077 |
| Largest diff. peak/hole / e $Å^{-3}$ | 0.41/-0.30 | 1.61/-0.48 | 0.39/-0.65 |
| Flack parameter | -0.010(8) | -0.005(15) | 0.005(6) |

Table S1. Crystal data and structure refinement for CuL-water, CuL-MeOH and CuL-ACN.

## Spectra and Results

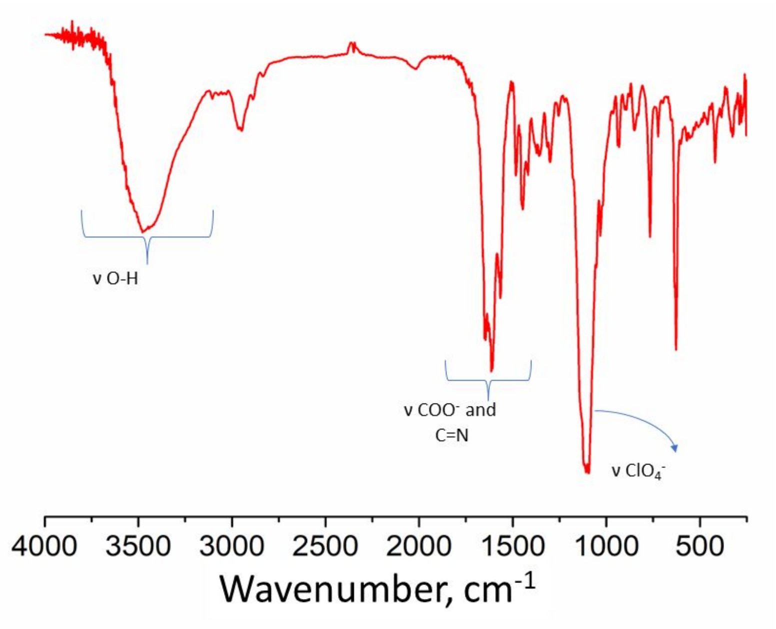


Figure S1. FTIR spectra of complex **4** obtained from KBr pellets.

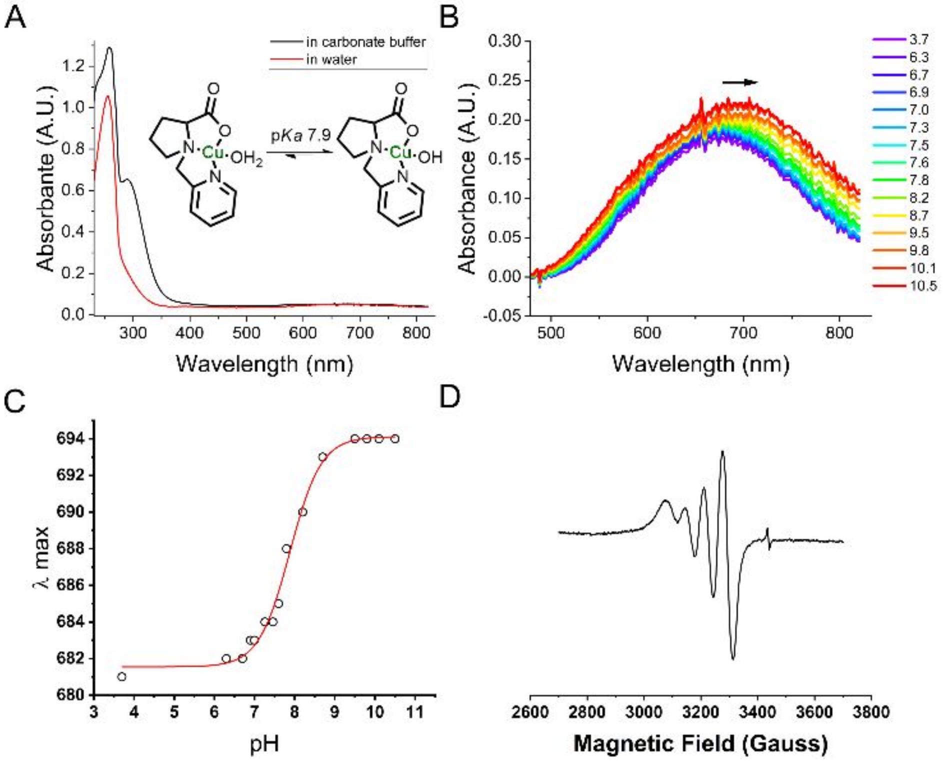

Figure S2. Electronic spectroscopy of complex **4** in the UV-Vis region in water (pH=5, red line) and carbonate buffer (pH-10.5, black line) (A). The d-d band shifts from 668 to 694 $cm^{-1}$ upon basification of the system (B) and this shift is associated with a *pKa* of 7.9 (C). The RT-EPR of complex **4** in carbonate buffer pH 10.5 is shown in (D), evidencing the presence of a single species in solution (the high field line is a g-marker of $Cr^{III}$ in MgO crystal, g = 1.9797).

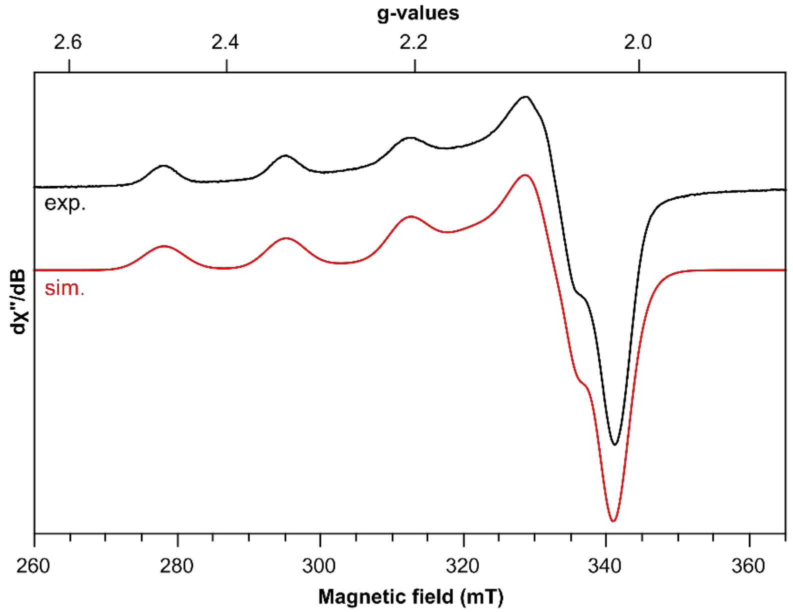


Figure S3. Perpendicular-mode X-band electron paramagnetic resonance spectrum at 30 K of a 1 mM frozen solution of **4** in 100 mM carbonate buffer at pH 10.5. Frequency 9.6351 GHz, power 0.25 mW, modulation amplitude 0.75 mT, modulation frequency 100 MHz. Best-fit simulation (red trace) for $g$ = [2.056 2.089 2.266] and $A$ = [50 94 530] MHz = [16.68 31.36 176.79]×$10^{-4}$ $cm^{-1}$.

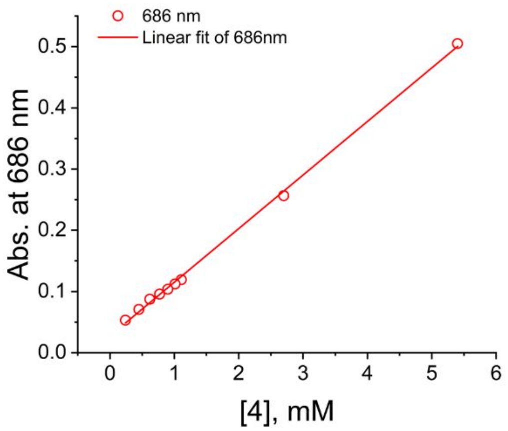

Figure S4. Absorbance of the d-d band of complex **4** at different concentrations. This experiment reveals the linear correlation between concentration and absorption, indicating that complex 4 in solution behaves as a single molecule and not as a dynamic polymeric structure.

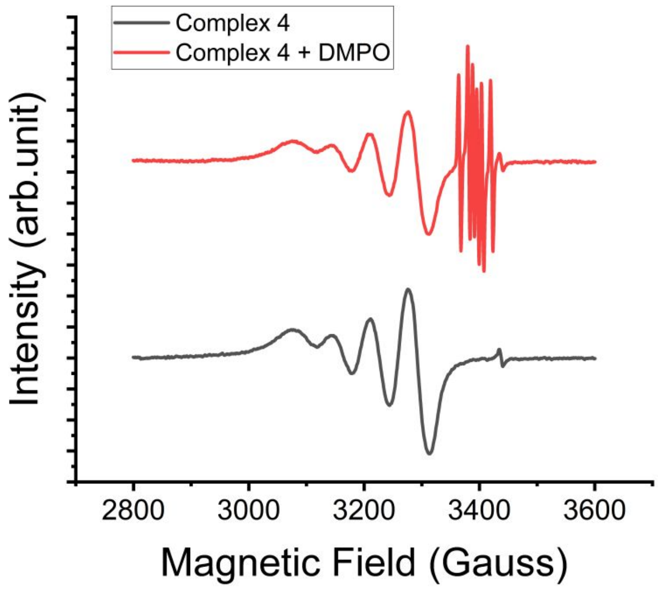


Figure S5. EPR spectra of complex **4** in the presence and absence of DMPO. It is visible that the copper center remains unaltered upon DMPO addition.

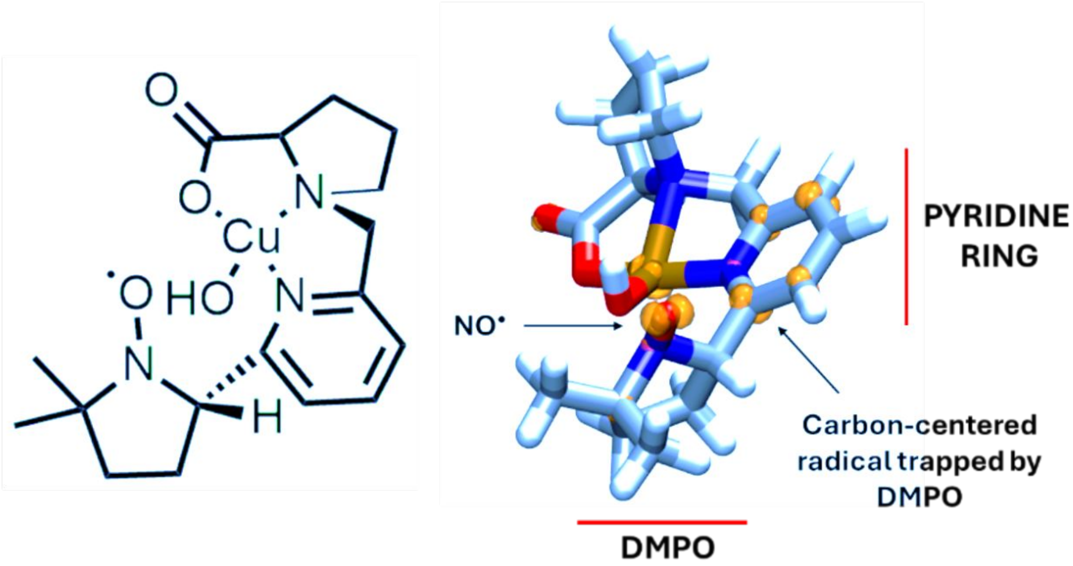


Figure S6. DFT calculation of the SOMO orbital of the C-centered DMPO spin adduct proposed as radical 1. The spin density of the SOMO orbital is shown in orange color.

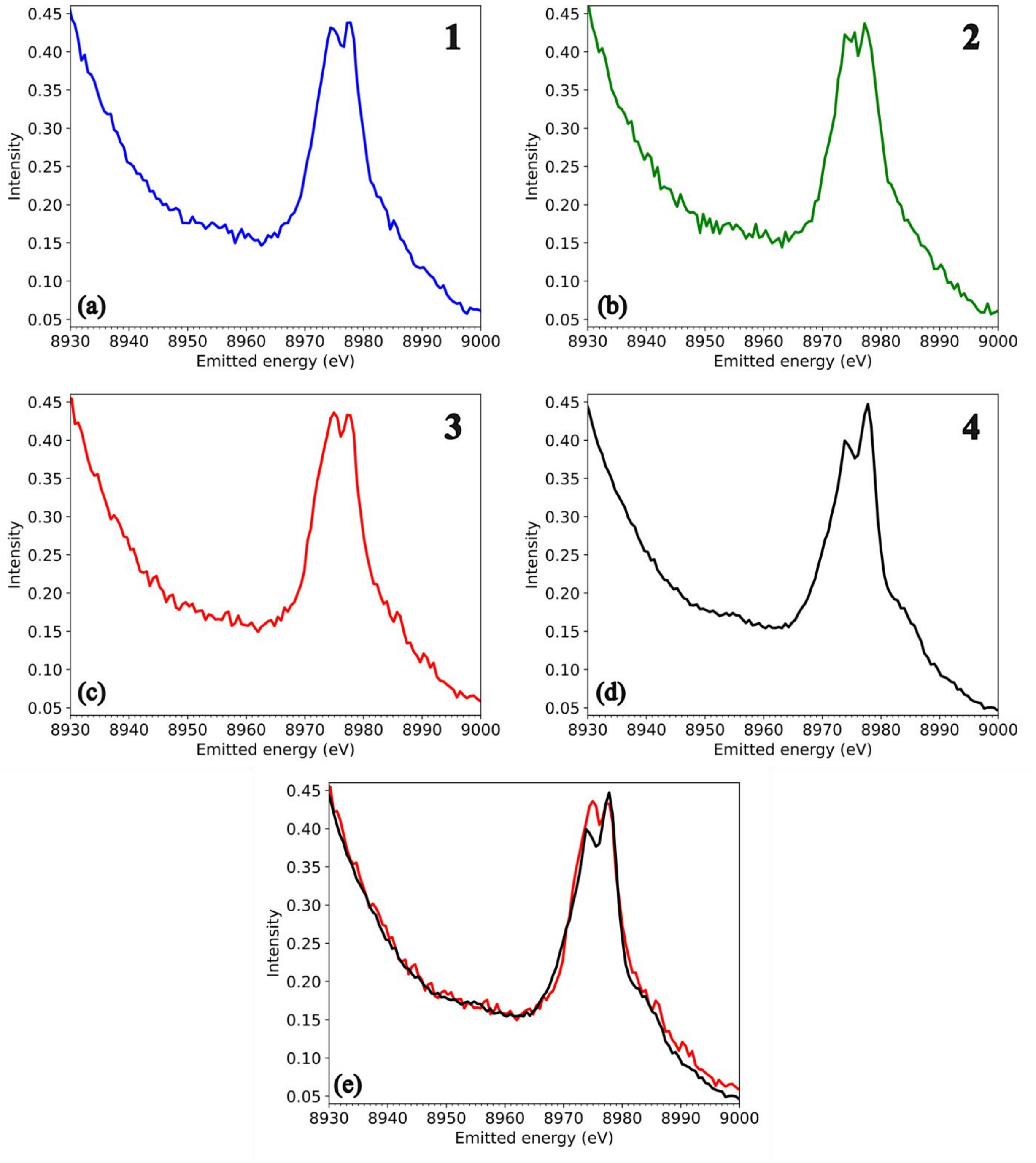


Figure S7. Cu Kβ X-ray emission spectra at the valence-to-core region of complexes (a) **1**, (b) **2**, (c) **3** and (d) **4**, and (e) overlaid spectra of **3** and **4**. The $K\beta_{2,5}$ features at 8965-8980 eV, associated with transitions from valence molecular orbitals with main N(2p) character to the Cu(1s) core hole, are similar for the N,N,N complexes **1**-**3**. However, in **4** the low-energy feature (8973 eV) is broader and the high-energy feature (8977 eV) is sharper relative to **1**-**3**. This slight but noticeable redistribution of intensity towards lower emitted energies (clearer in e) is consequence of the more stabilized valence orbitals involving O(2p) owing the N,N,O donor set of **4**.

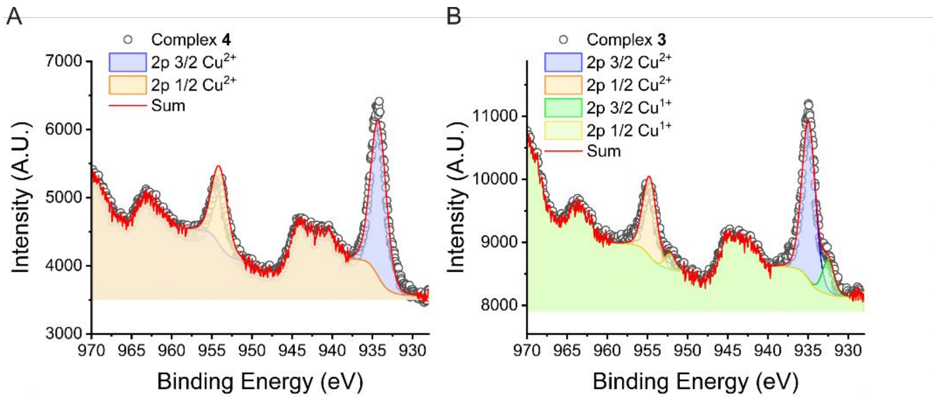


Figure S8. XPS Cu2p of complexes **4** and **3**.

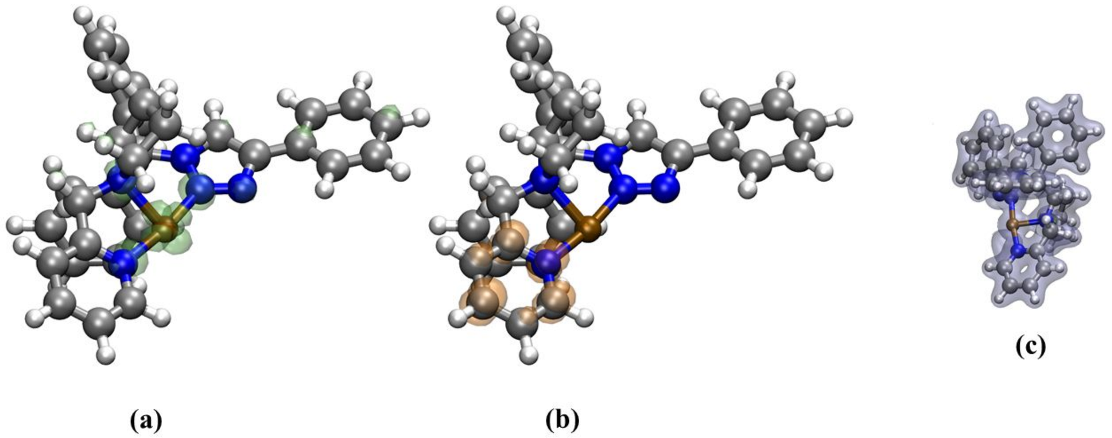


Figure S9. (a) HOMO (b) LUMO (c) Electronic Density surfaces. Isovalues equal to 0.05 |*e*| Å$^{-3}$. Color-code: Cu (ochre); N (blue); C (gray); H (white); HOMO (green surface); LUMO (orange surface); Electronic Density (ice-blue surface).

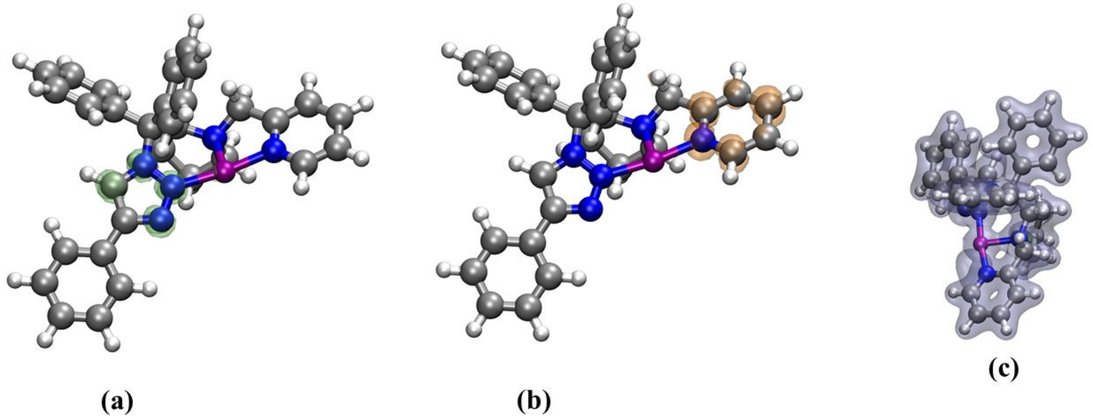


Figure S10. (a) HOMO (b) LUMO (c) Electronic Density surfaces. Isovalues equal to 0.05 |*e*| Å$^{-3}$. Color-code: Zn (purple); N (blue); C (gray); H (white); HOMO (green surface); LUMO (orange surface); Electronic Density (ice-blue surface).

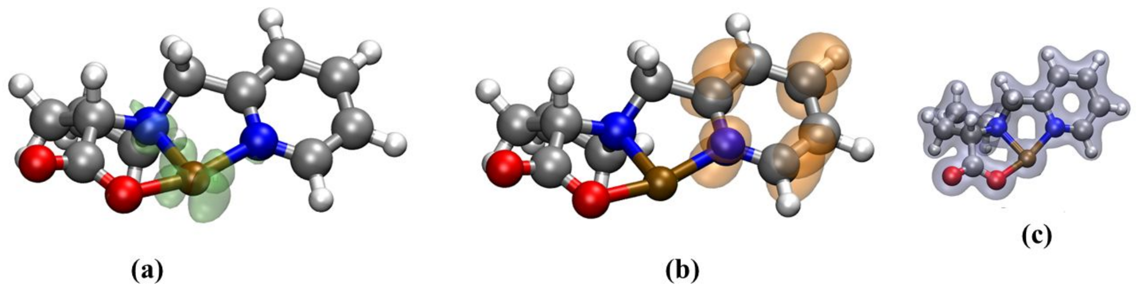


Figure S11. (a) HOMO (b) LUMO (c) Electronic Density surfaces. Isovalues equal to 0.05 |*e*| Å$^{-3}$. Color-code: Cu (ochre); O (red); N (blue); C (gray); H (white); HOMO (green surface); LUMO (orange surface); Electronic Density (ice-blue surface).

Table S2 - Wiberg/Mayer bond order for complexes **3**, **3-Zn** and **4**.

| Complex | Bonding | Wiberg | Mayer |
|---|---|---|---|
| **- 3** | Cu – N1 | 0.6 | 0.4 pirrol |
| | Cu – N2 | 0.2 | 0.1 piridina |
| | Cu – N3 | 0.6 | 0.4 triazol |
| **3-Zn** | Zn – N1 | 0.5 | 0.4 |
| | Zn – N2 | 0.4 | 0.3 |
| | Zn – N3 | 0.5 | 0.4 |
| **4** | Cu – N1 | 0.5 | 0.4 |
| | Cu – N2 | 0.2 | 0.1 |
| | Cu – O | 0.5 | 0.4 |

Table S3. HOMO and LUMO orbitals contributions.

| **Frontier Orbitals** | **Species** | | |
|---|---|---|---|
| | **3** | **3-Zn** | **4** |
| **HOMO** | 78% Cu-d<br>22% N-p | 56% N-p<br>44% C-p | 84% Cu-d<br>15% N-p<br>1% O-p |
| **LUMO** | 64% C-p<br>34% N-p | 71% C-p<br>28% N-p | 79% C-p<br>19% N-p |

| | | | |
|---|---|---|---|
| | 2% H-s | 1% H-s | 2% H-s |

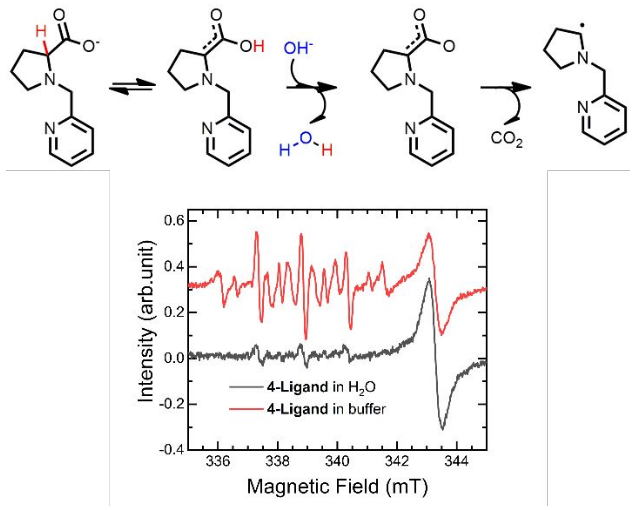


Figure S12. Scheme of ligand tautomerization and EPR experiment of DMPO trapping of the ligand in water pH 7 and buffer pH 10.5. The concentration of radicals trapped in buffer are 1% of the expected, whereas the concentration of the radicals in water are less than 0.1%.

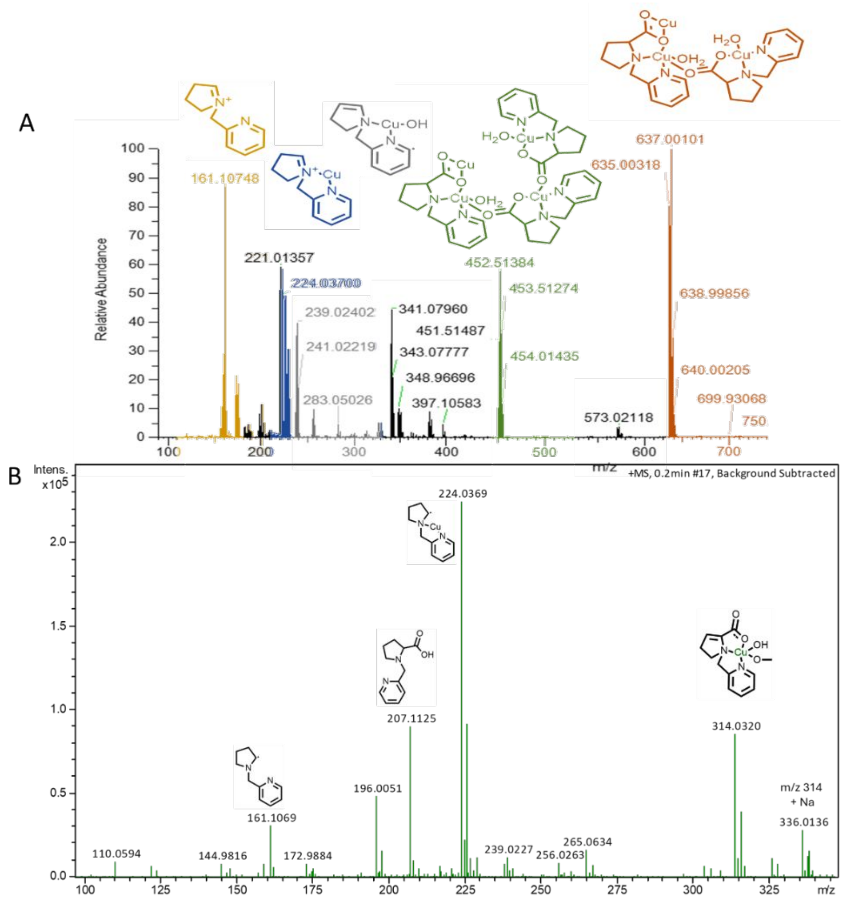


Figure S13. HRMS of complex **4** is dependent on the solution concentration. The HRMS of a highly concentrated methanolic solution (A) reveals peaks corresponding to a polymeric nature (Proposed structure that corresponds to the peak at m/z 452.51384 (2+ charge) and molecular formula of $C_{33}H_{43}Cu_4N_6O_8$ (theoretical mass 451.51577). Peak at m/z 161.1074 corresponds to the ligand without the carboxylic group ($C_{10}H_{13}N_2$ calc. 161.1078). Peak at m/z 224.0370 corresponds to the monomeric species of the complex without the carboxylic group ($C_{10}H_{13}CuN_2$ calc. 224.0374). Peak at m/z 635.0031 corresponds to another polymeric specie ($C_{22}H_{30}Cu_3N_4O_6$ calc. 635.0053)). The HRMS of a low concentration methanolic solution (B) reveals peaks corresponding to the monomer (peak at m/z 314.0320 corresponds to $C_{12}H_{15}CuN_2O_4$ calc. 314.0327, and m/z 336.0136 corresponds to the sodium salt of 314.0320).

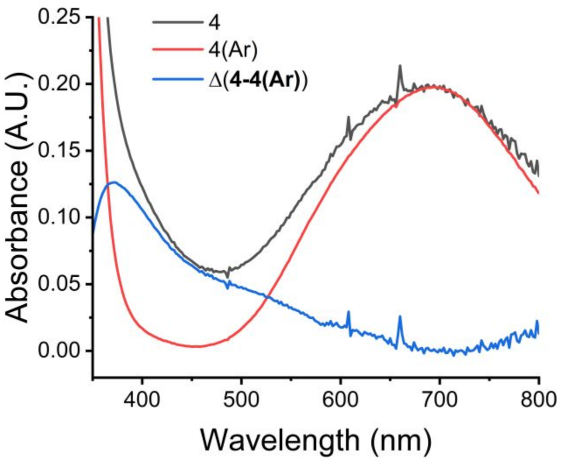


Figure S14. Electronic spectroscopy at the UV-Vis region of complex **4** and complex **4(Ar)** in water solutions. The blue line is the difference between the spectrum of **4** and the spectrum of **4(Ar)**, revealing that bands at 372 nm and 500 nm are only present in the complex that has been sitting at aerobic conditions (synthesis, storage and handling).

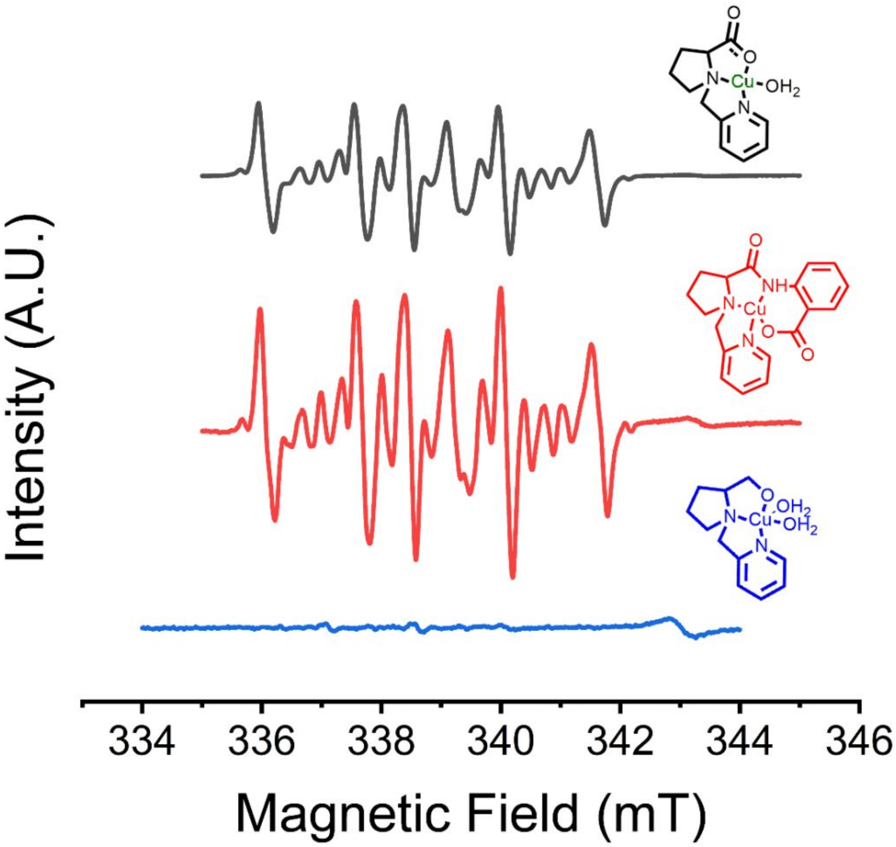


Figure S15. EPR spectrum of the DMPO trapped experiment of complex **4** and similar complexes with different functionalities close to the chiral carbon (**4-alcohol** and **4-amide**). The experiments were performed in carbonate buffer solution using 100mM of DMPO and 1 mM of complex.

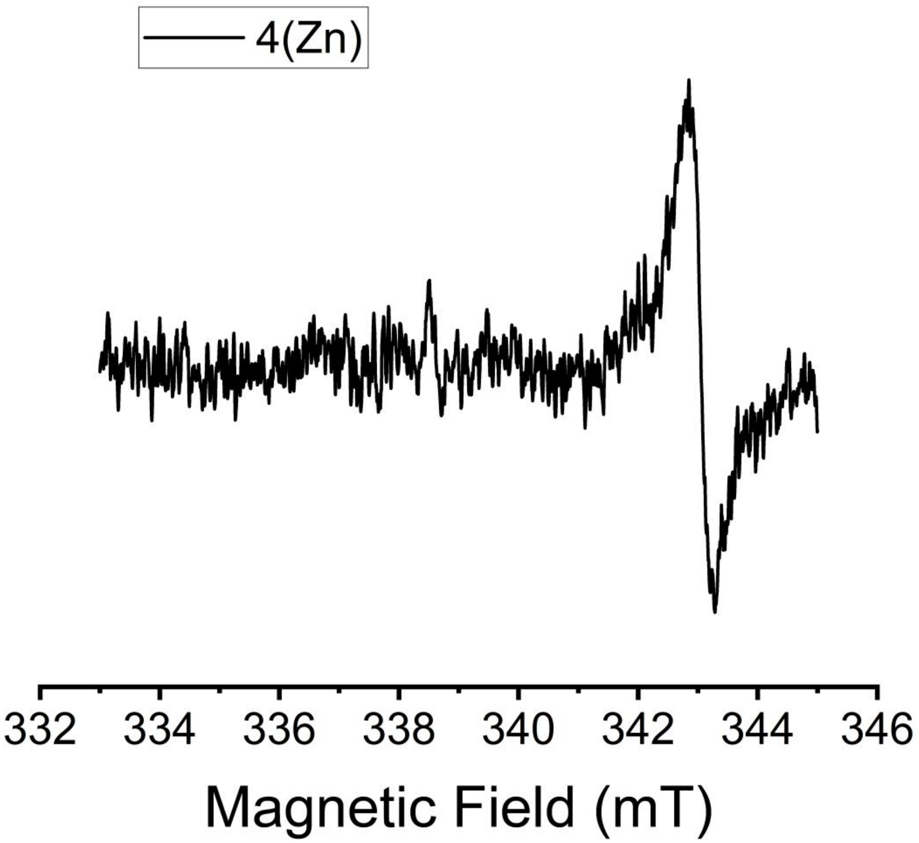


Figure S16. EPR spectrum of the DMPO trapped experiment of complex 4(Zn) in carbonate buffer solution.

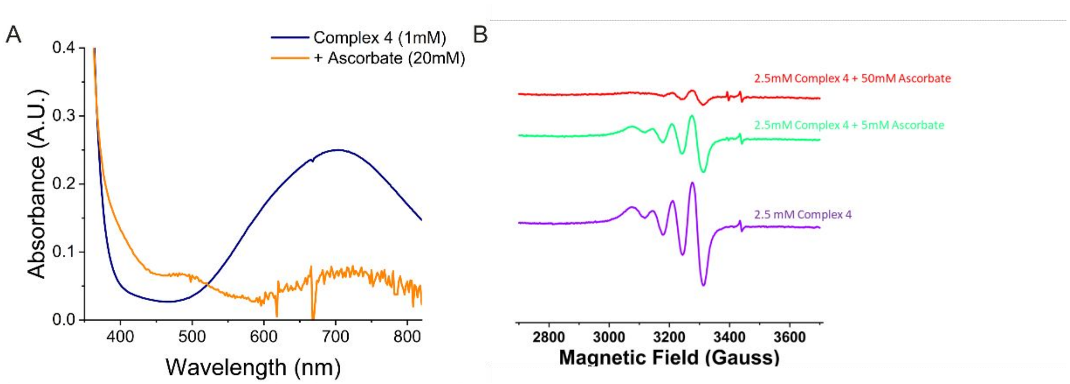


Figure S17. Effect of the addition of ascorbate at complex 4. (A) UV-Vis spectra of complex 4 at 1mM concentration dissolved in 0.1M carbonate buffer pH 10.5 before (blue line) and after the addition of ascorbate (final concentration 20 mM, orange line). (B) EPR spectra at room temperature of complex 4 at 2.5 mM concentration dissolved in 0.1M carbonate buffer pH 10.5 (purple line) and in the presence of 5mM ascorbate (green line) and 50 mM ascorbate (red line).

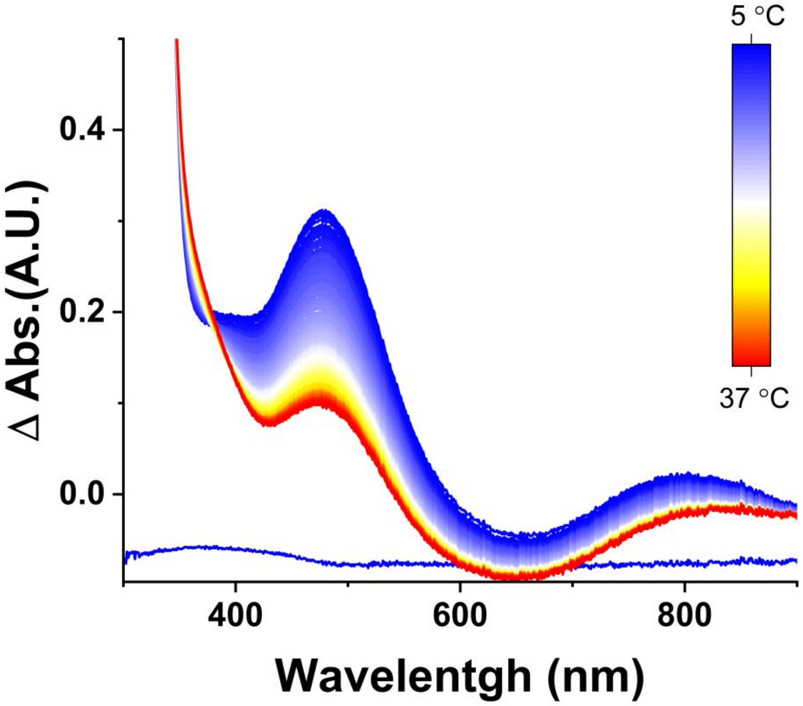


Figure S18. Effect of the increase of temperature of a solution of **4ox1**. Complex **4** at 1mM concentration dissolved in 0.1M carbonate buffer pH 10.5 after the addition of ascorbate in aerobic conditions.

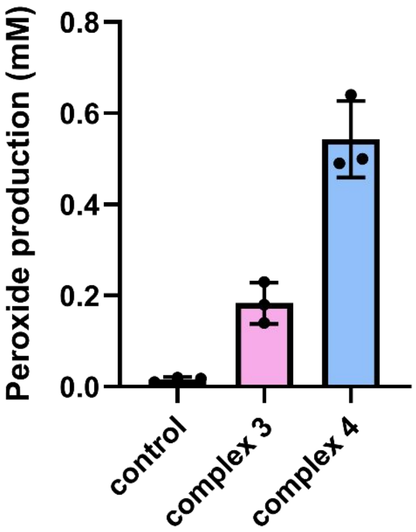

Figure S19. Concentration of hydrogen peroxide detected using a thiocyanate assay after bubbling a solution of solvent (blank), complex **3** or complex **4** with dioxygen. All complexes were used at 2.5mM concentration in carbonate buffer solution.

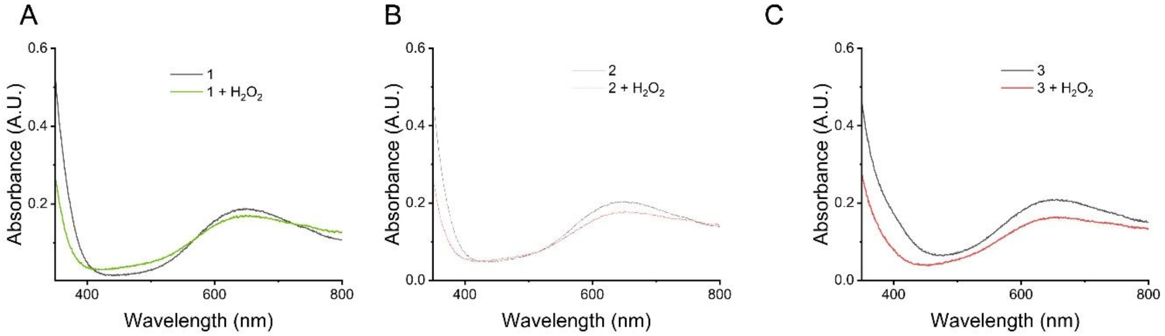


Figure S20. UV-Vis spectroscopy of the reaction of peroxide with complexes **1** (A), **2**(B) and **3**(C).

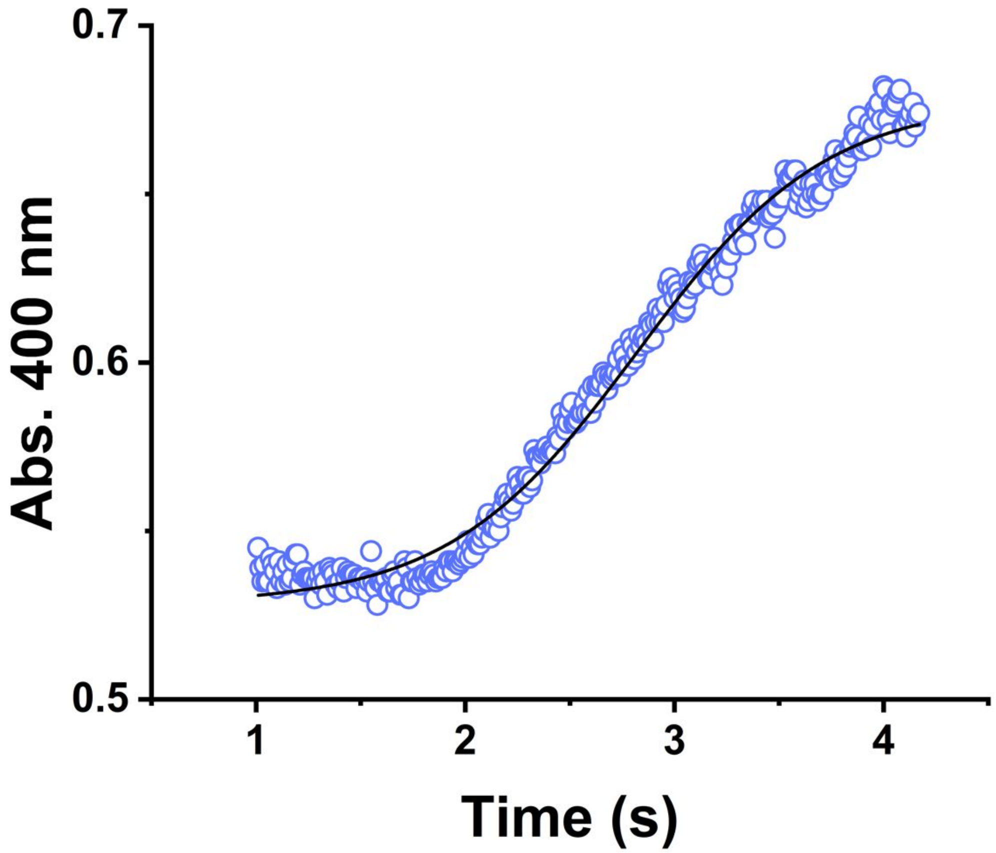


Figure S21. UV-Vis monitoring of the emergence of the band at 400 nm from the reaction between **4** and hydrogen peroxide. The reaction was monitored at 5°C, using 1mM of complex 4 in 100mM carbonate buffer (pH=10.5).

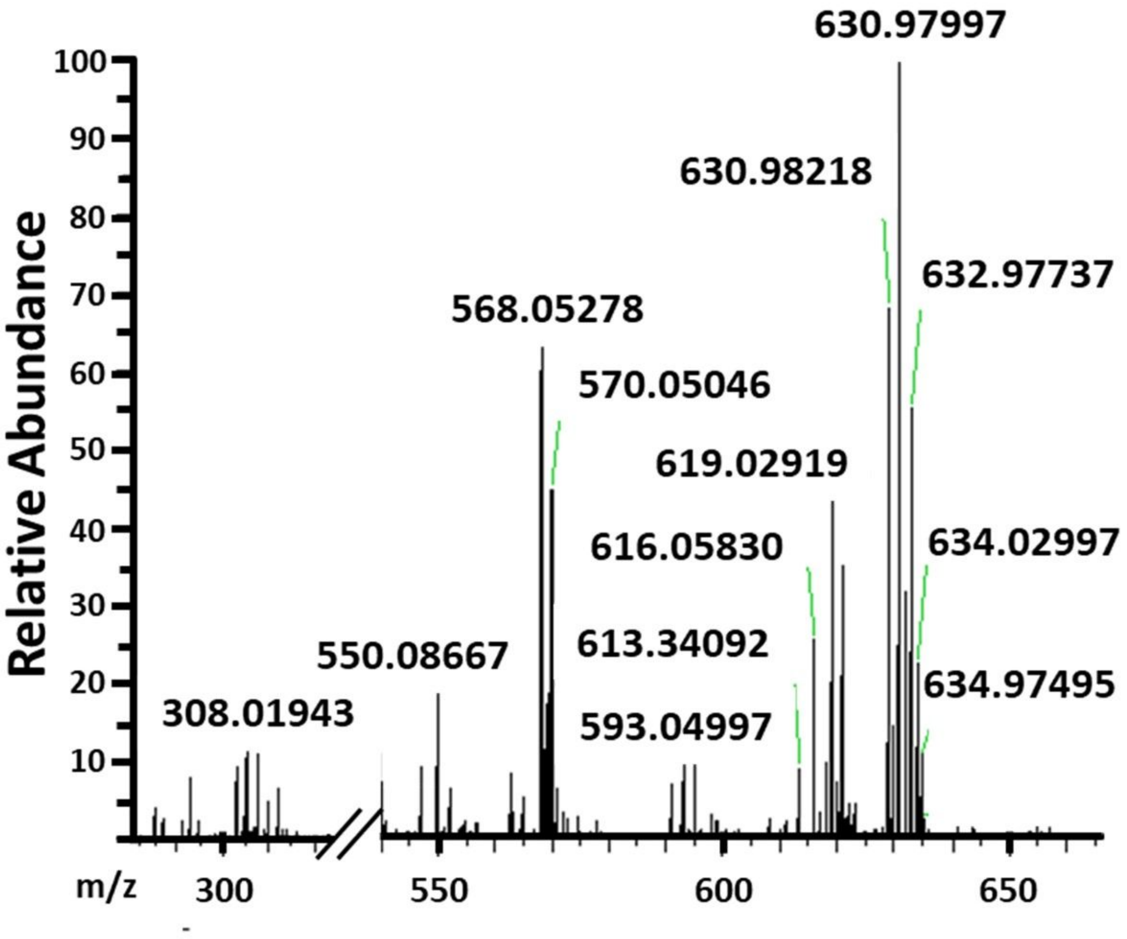


Figure S22.HRMS of complex **4** in the presence of peroxide.

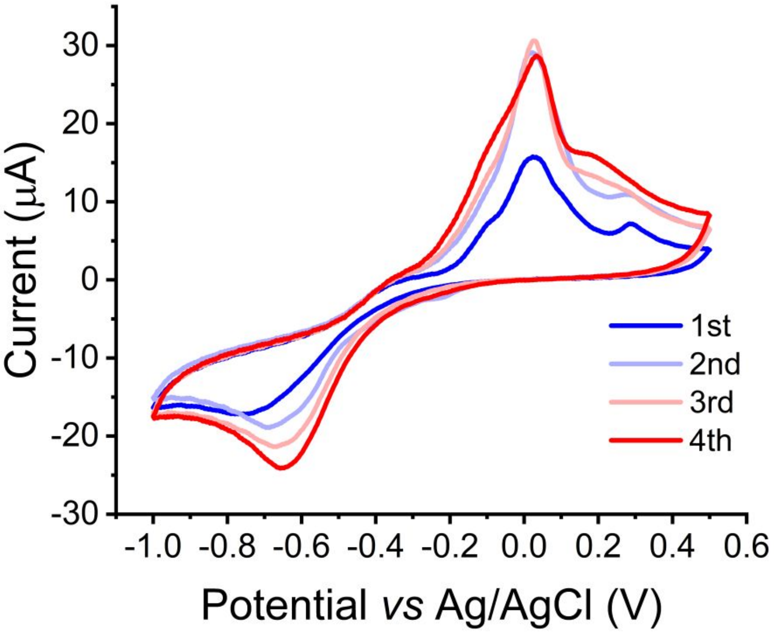


Figure S23.Cyclic Voltammogram of complex **4(Ar)** in carbonate buffer pH 10.5 0.1M at 100mVs$^{-1}$ scan rate. A cell containing three electrodes was employed: vitreous carbon (WE), platinum (CE) and Ag/AgCl 3.5M (Reference). The cathodic scan started at 0V.

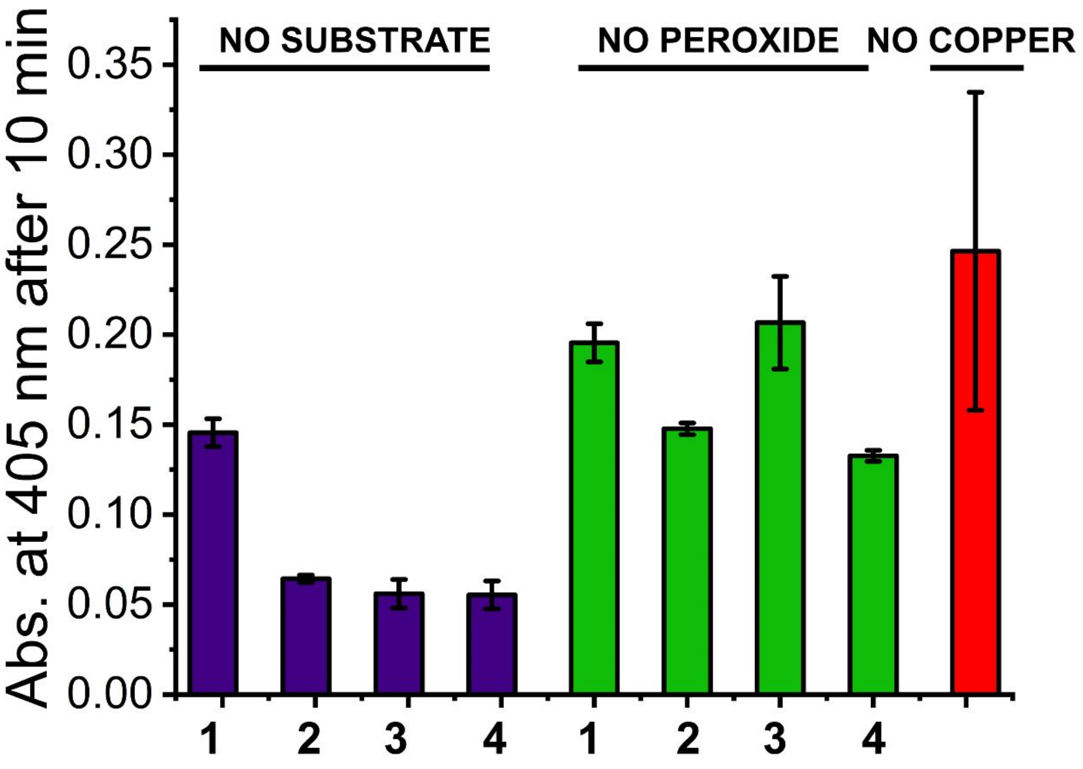


Figure S24. **Controls of the reactions.** The reactions were monitored at 405 nm by UV-Vis spectroscopy. For the assays without substrate we employed 20 mM of peroxide and 1mM of complex. For the assays without peroxide we employed 20 mM of substrate and 1mM of complex. The reaction without copper used 20 mM of substrate and 20 mM of peroxide. Total volume of the reactions was 100µL and reaction temperature of 37˚C. All assays were performed in 100mM carbonate solution (pH=10.5) and in triplicate and are represented as mean and standard deviation.

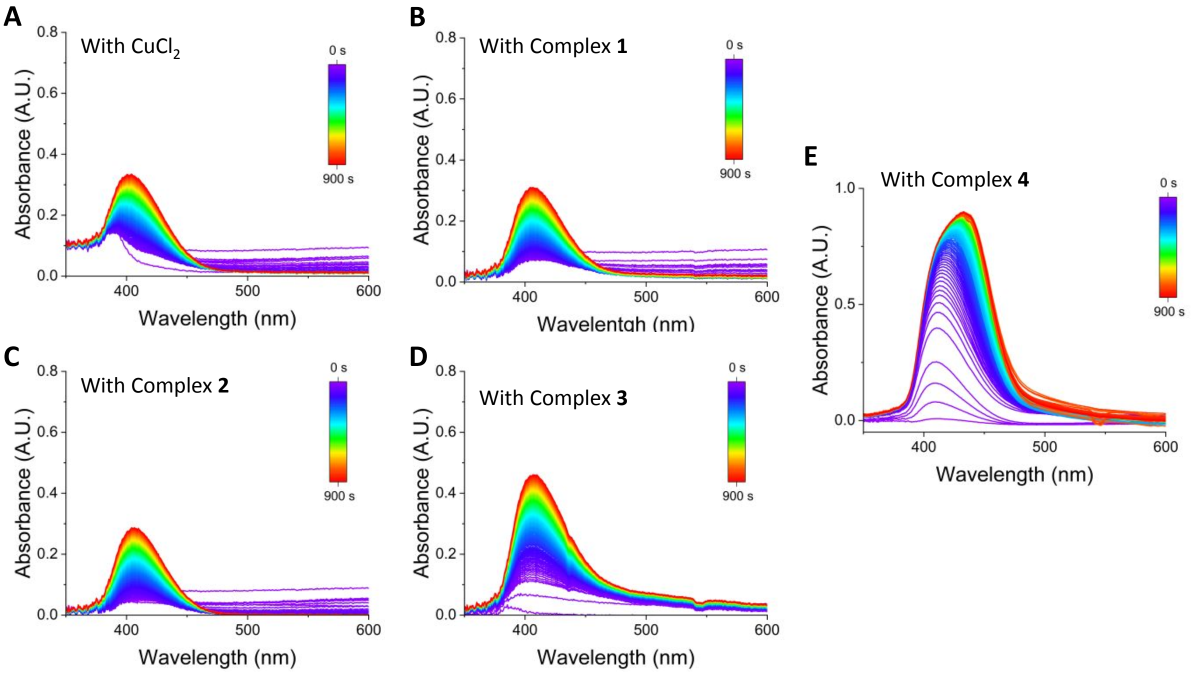


Figure S25. **Kinetics of the oxidative cleavage of 4-nitrophenyl-β-D-glucopyranoside by copper complexes.** The reactions were monitored by UV-Vis spectroscopy over time at 405nm. All reactions were performed with 0.1 mM of the copper compound in a 100 mM carbonate solution (pH=10.5), 20 mM $H_2O_2$ and 20 mM substrate. The cuvette containing all components was used as a blank. The reaction started after addition of the copper compound as shown in: (A) reaction with **$CuCl_2$**, (B) reaction with **1**, (C), reaction with **2**, (D) reaction using **3,** (E) reaction using **4**.

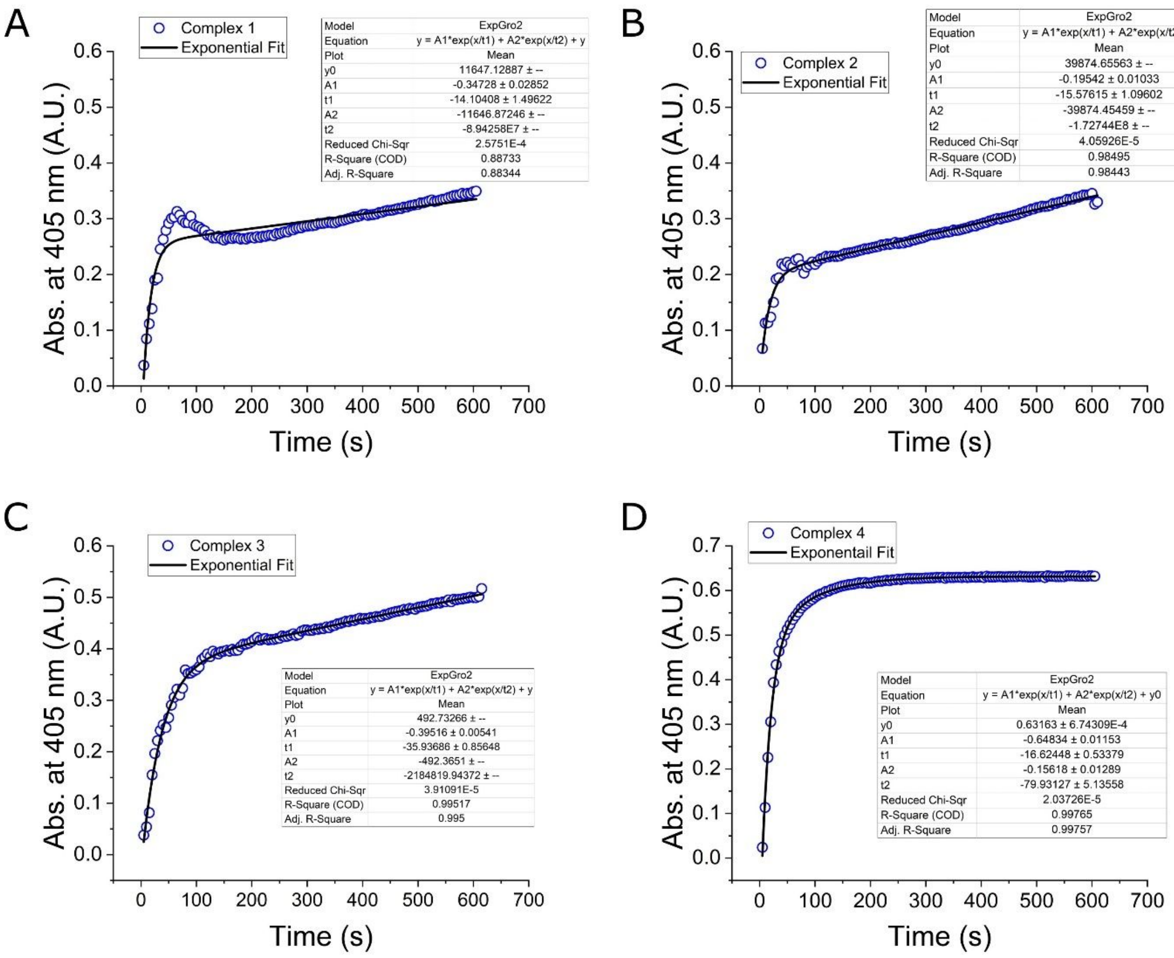


Figure S26. **Double Exponential Fits of the kinetics of the oxidative cleavage of 4-nitrophenyl-β-D-glucopyranoside by copper complexes.** The reactions were monitored by UV-Vis spectroscopy over time at 405nm. All reactions were performed with 0.1 mM of the copper compound in a 100 mM carbonate solution (pH=10.5), 20 mM $H_2O_2$ and 20 mM substrate. The cuvette containing all components was used as a blank. The reaction started after addition of the copper compound as shown in: (A) reaction with **1**, (B) reaction with **2**, (C), reaction with **3** and (D) reaction using **4**. The mean values of the kinetics were fit to the ExpGro2 function in Origin 2022 software. The kinetic constants $k_{obs}$ were obtained by calculating $-1/t_1$ and $-1/t_2$.

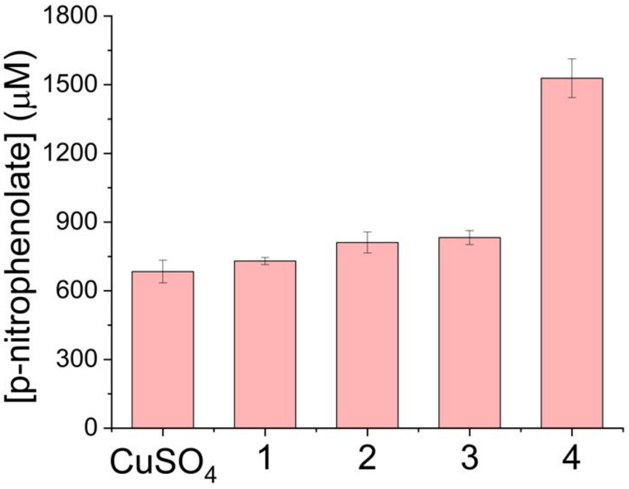


Figure S27. **24 hours oxidative activity.** The reactions were monitored at 405 nm by UV-Vis spectroscopy after 24 hours of incubation at 37˚C. For these assays, 100mM carbonate solution (pH=10.5), 20mM of substrate, 20mM of hydrogen peroxide and 1mM copper complex were used. All assays were performed in triplicate and are represented as mean and standard deviation.

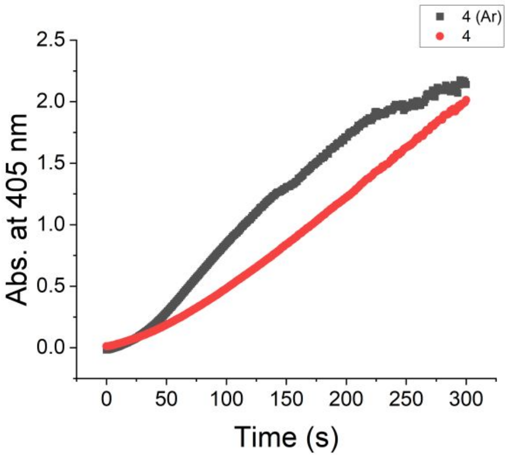


Figure S28. Reaction kinetics of the oxidative degradation of p-nitrophenyl-β-D-glucopyranoside using **4** and a freshly synthesized **4** under anaerobic conditions **4(Ar)**.

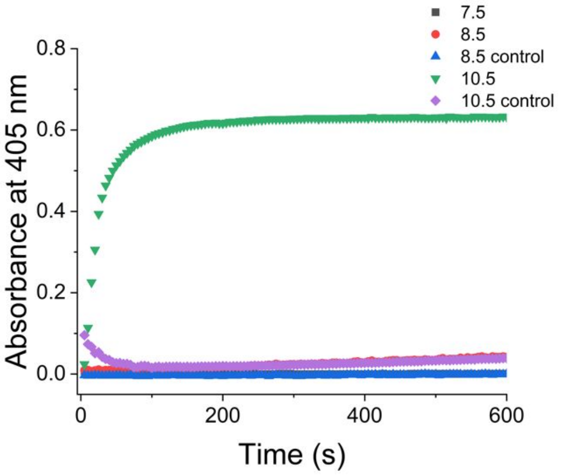


Figure S29. **Influence of the reaction pH on the reaction kinetics.** Variation of pH (7.5, 8.5 and 10.5) and the absorbance at 405nm over time. The increase in the pH increases the kinetics. For these assays, 100mM buffer solution, 20mM of substrate, 20mM of hydrogen peroxide and 1mM complex 4 were used. Control reactions were performed in the absence of the catalyst.

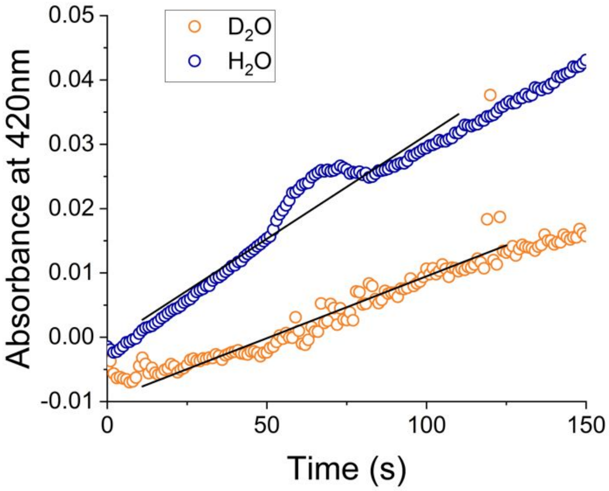


Figure S30. Kinetic Isotope Effect of the reaction of 4-nitrophenyl-□-D-glucopyranoside degradation. The reaction was performed using 20mM of substrate, 1mM of complex 4 and 20mM of $H_2O_2$ either in deuterated carbonate buffer (orange dots) or in normal carbonate buffer (blue dots) at pH 10.5 and 100mM concentration.

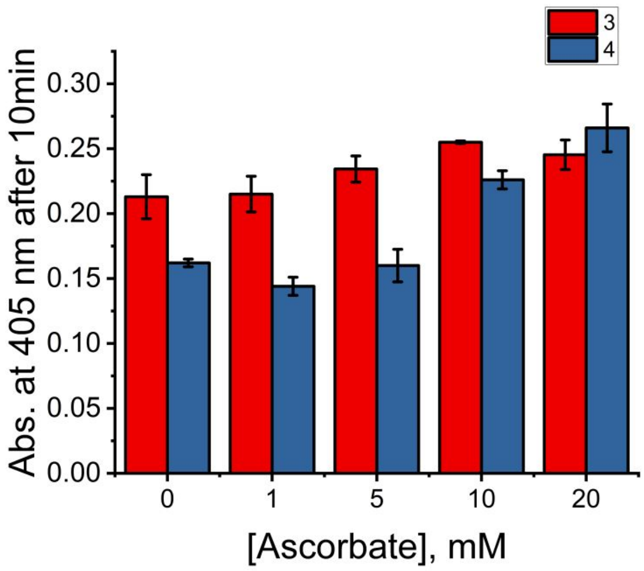


Figure S31. **Influence of the ascorbate concentration in catalysis.** The absorbance at 405nm was monitored after 10 minutes of reaction. For these assays, a 100mM buffer solution, 20mM of substrate and 1mM of complex **3** or **4** were used at a reaction temperature of 37˚C.

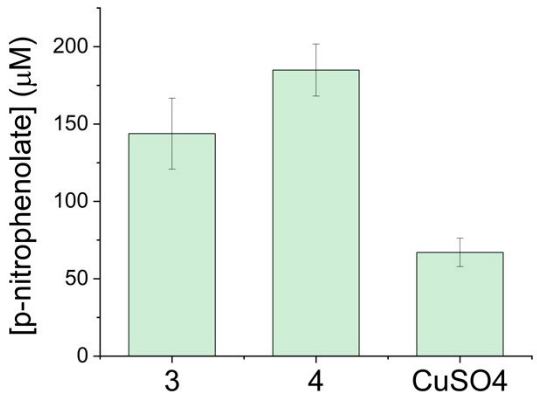


Figure S32. **24 hours oxidative activity.** The reactions were monitored at 405 nm by UV-Vis spectroscopy after 24 hours of incubation at 37˚C. For these assays, 100mM carbonate solution (pH=10.5), 20mM of substrate, 20mM of sodium ascorbate and 1mM of copper complex were used. All assays were performed in triplicate and are represented as mean and standard deviation.

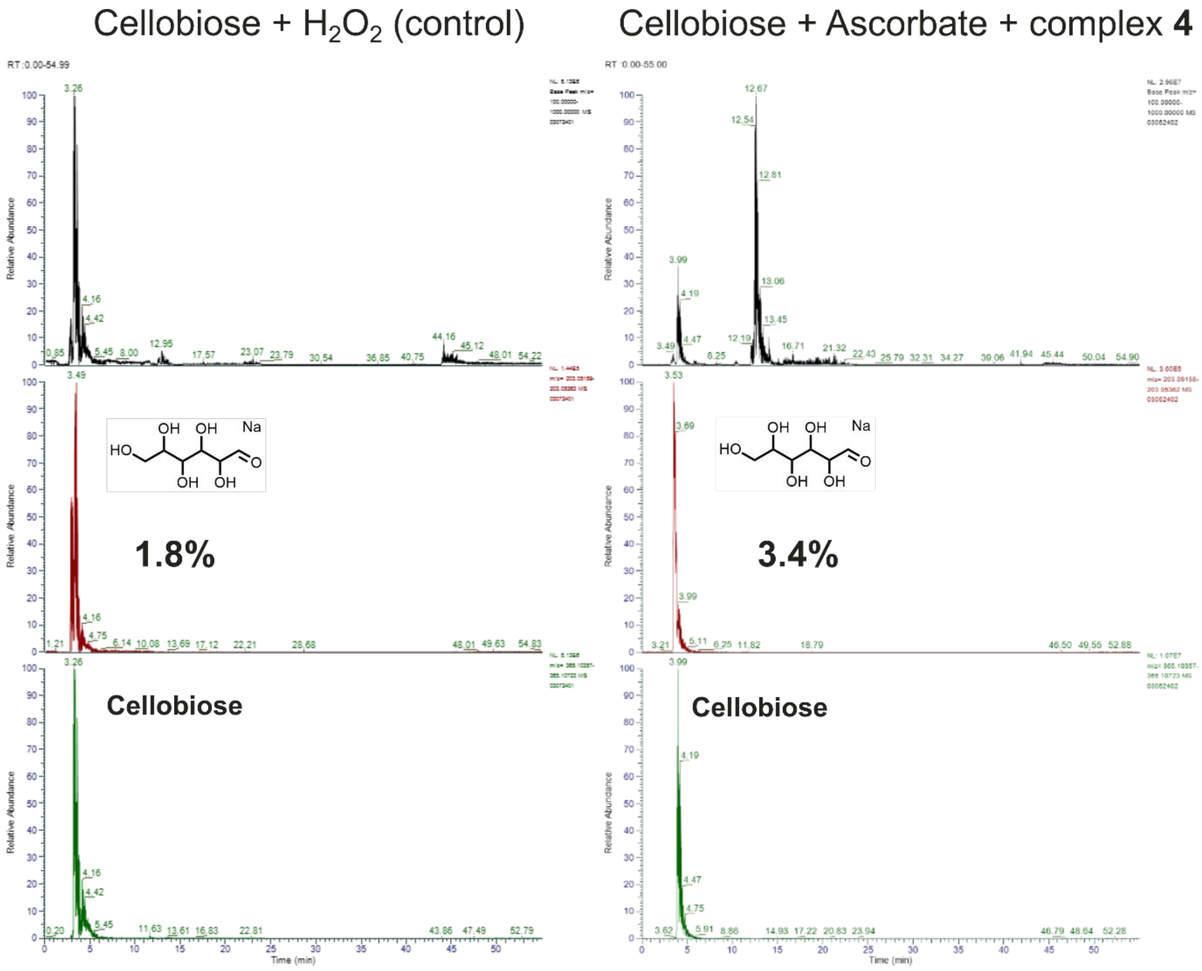


Figure S33. HPLC-MS of the decomposition reaction of cellobiose in the presence of 4 and ascorbate.

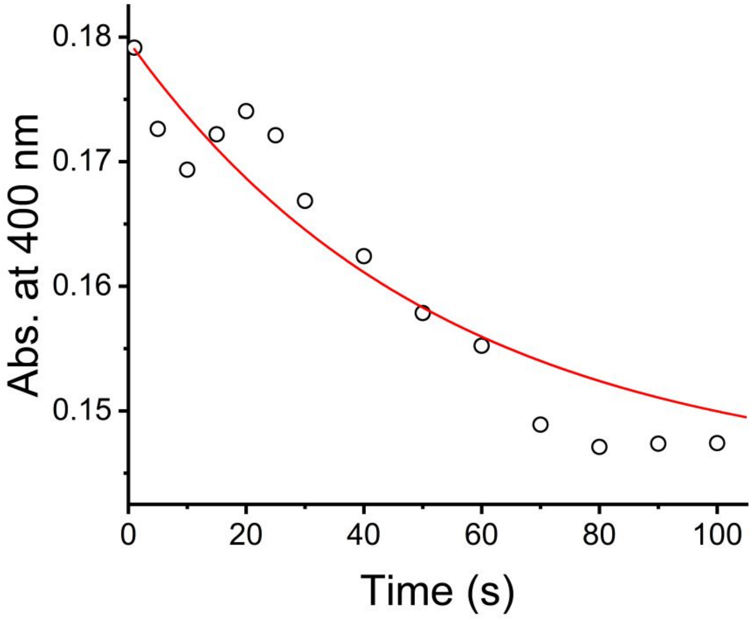


Figure S34. **Consumption of intermediate 4ox2 by cellobiose.** The reaction was performed with 1 mM of the compound **4** in bicarbonate buffer pH= 10.5 and 5 mM of $H_2O_2$ to obtain **4ox2**. Once it was formed, the absorbance at 400 nm was monitored after the addition of cellobiose. The experiment was performed at 5°C to ensure stability of the intermediate.

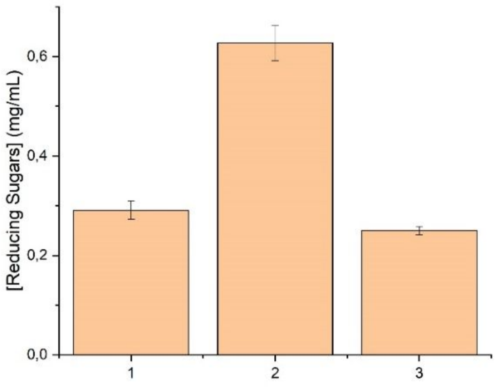


Figure S35. **Complex 4 activity against filter paper as a substrate with and without additives.** The reactions were performed with 5 mM of the compound **4** in bicarbonate buffer pH= 10.5 and filter paper (0.5 x 0.75 mm). (1) reaction in bicarbonate buffer, (2) reaction with ascorbate 10 mM, (3) reaction with $H_2O_2$ 10 mM. The reactions were monitored by a glucose standard curve using the DNS method. Sample containing all components without the complex **4** was used as a blank.

A

B

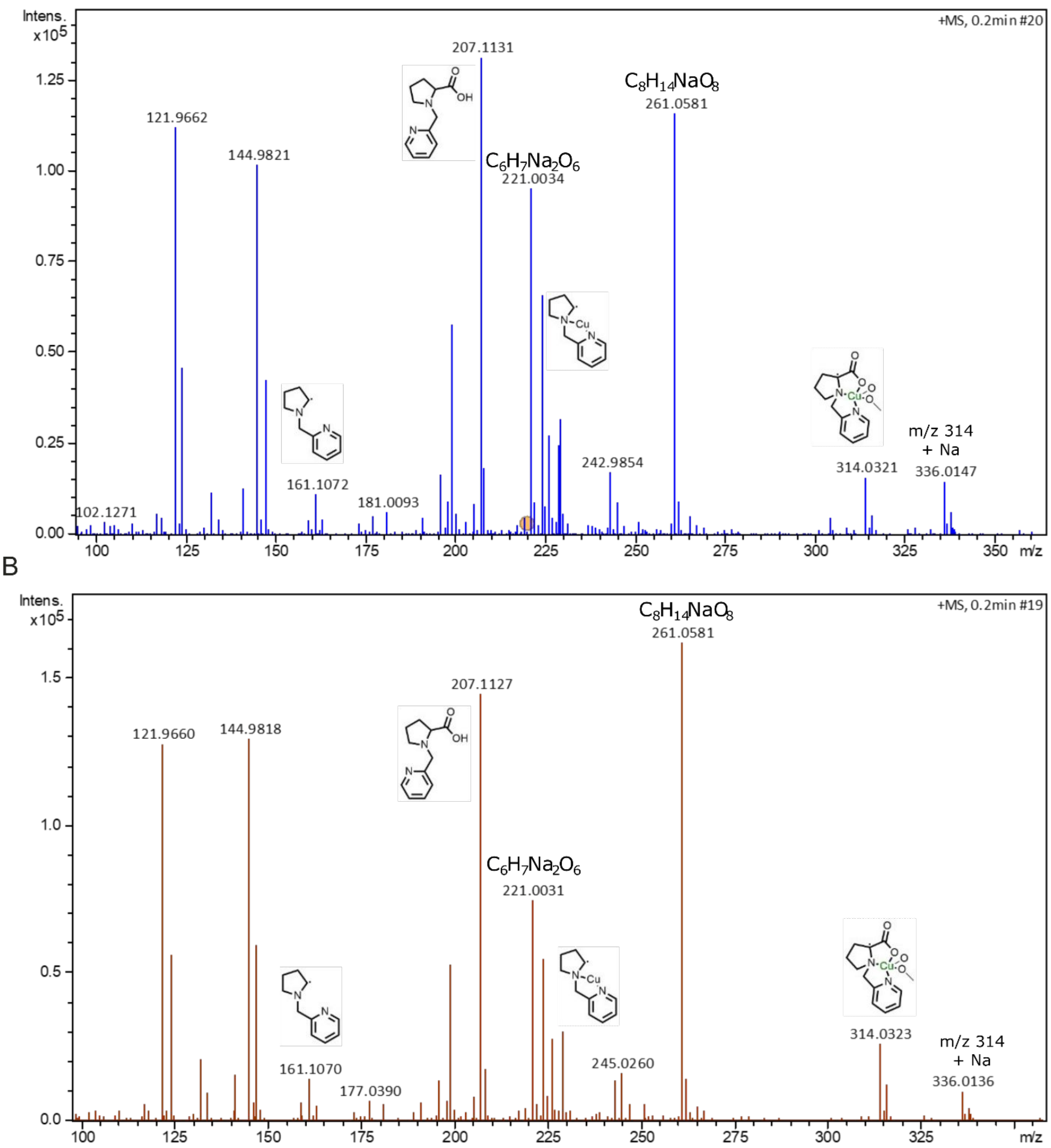


Figure S36. HRMS of reaction between **4** and ascorbate after 5 minutes (A) and after 20 minutes of reaction (B). The spectra evidence the presence of the intact complex, free ligand and ascorbate related products.

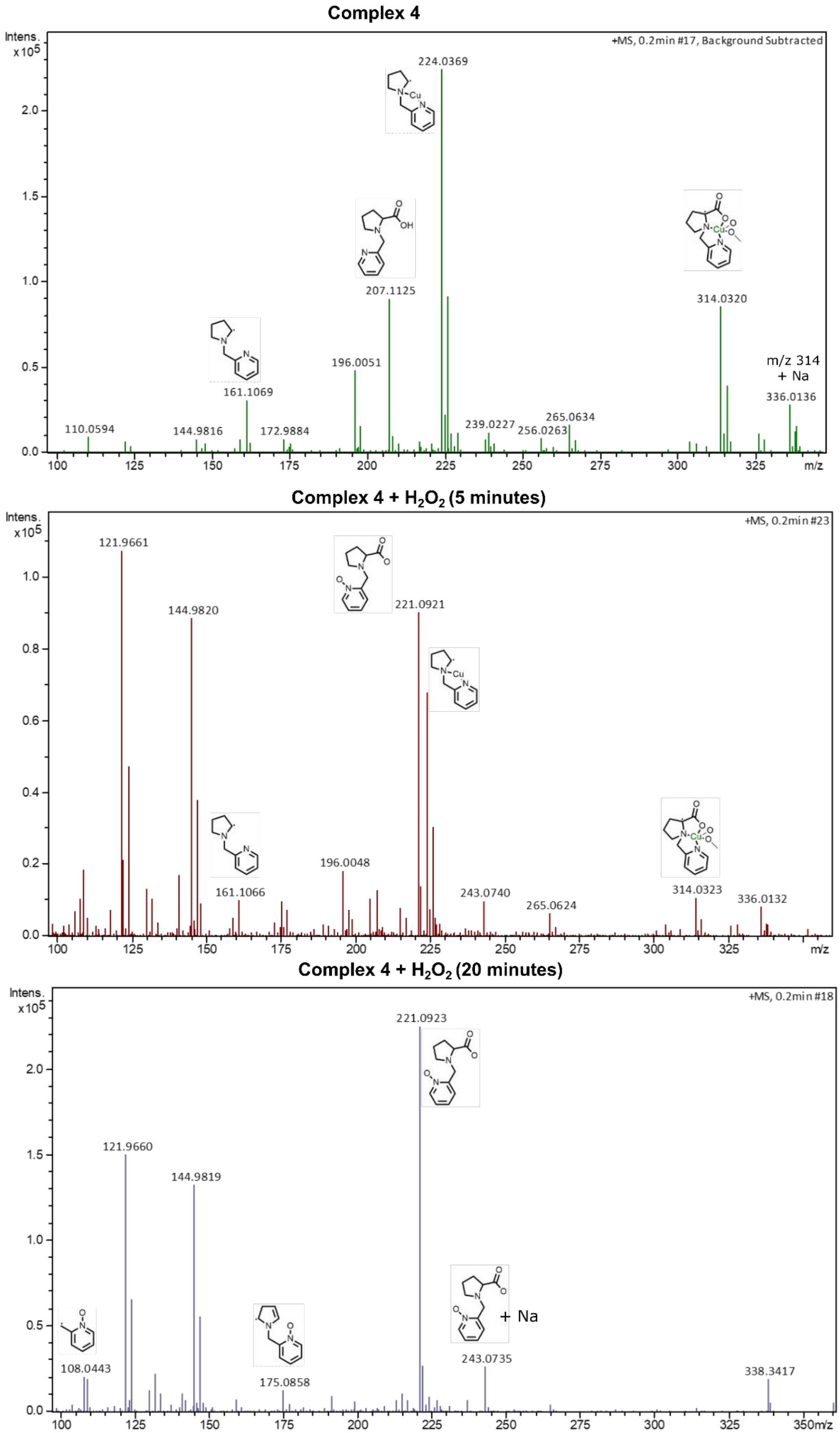


Figure S37. Time Resolved HRMS of the reaction between complex **4** and peroxide.

Table S4 – BCP descriptors for the Cu – O and O – O bonding.

| Complex | Chemical means | $\rho_{BCP}(r_c)$ (\|e\| Å$^{-3}$ 10$^{-1}$) | $\nabla^2\rho_{BCP}(r_c)$ (\|e\| Å$^{-5}$ 10$^{-1}$) | Distance (Å) |
|---|---|---|---|---|
| **(a)** | Cu – O | 1.35 | 4.42 | 1.81 |
| | O – O | 4.22 | -2.10 | 1.29 |
| **(b)** | Cu – O | 1.50 | 9.10 | 1.76 |

Table S5 – Bader and Mulliken charges on the Cu center in the mechanism in the Fig. 9.

| System | Bader charge (\|e\|) | Mulliken charge (\|e\|) |
|---|---|---|
| **CuPH-OH** | 1.56 | 0.76 |
| **CuPH-$H_2O$** | 1.23 | 0.76 |
| **$Cu_2$PH-OO** | 0.89 – 1.35 | 0.66 – 0.70 |
| **2CuPH-O** | 1.15 – 1.16 | 0.64 – 0.64 |
| **TS1** | 0.98 – 1.12 | 0.63 – 0.67 |
| **TS2** | 1.19 – 1.21 | 0.62 – 0.62 |

Table S6 – Macchi's classification based on local (BCP) and integral properties.[14]

| Bond type | $\rho(r_c)$ | $\nabla^2\rho(r_c)$ | $G(r_c)$ | $V(r_c)$ |
|---|---|---|---|---|
| **Bonds between light atoms (Z < 18)** | | | | |
| Covalent | Large | << 0 | < 1 | << 0 |
| Intermediate | Large | Any value | ≥ 1 | << 0 |
| Closed-shell | Small | > 0 | ≥ 1 | ~ 0 |
| **Bonds between heavy atoms (Z > 18)** | | | | |
| Open-shell (e.g. Co-Co) | Small | ~ 0 | < 1 | < 0 |
| Donor-acceptor (e.g. Co-As) | Small | > 0 | ~ 1 | < 0 |

Table S7 – Bianchi classification based on local (BCP) and integral properties.[15]

| Bond type | $\rho(r_c)$ | $\nabla^2\rho_b$ | $G(r_c)$ | $V(r_c)$ |
|---|---|---|---|---|

| | | | | |
|---|---|---|---|---|
| Ionic | low | > 0 | $G_b/\rho_b \approx \|V_b/\rho_b\|$ | < 0 |
| Covalent | high | < 0 | $G_b/\rho_b << \|V_b/\rho_b\|$ | << 0 |

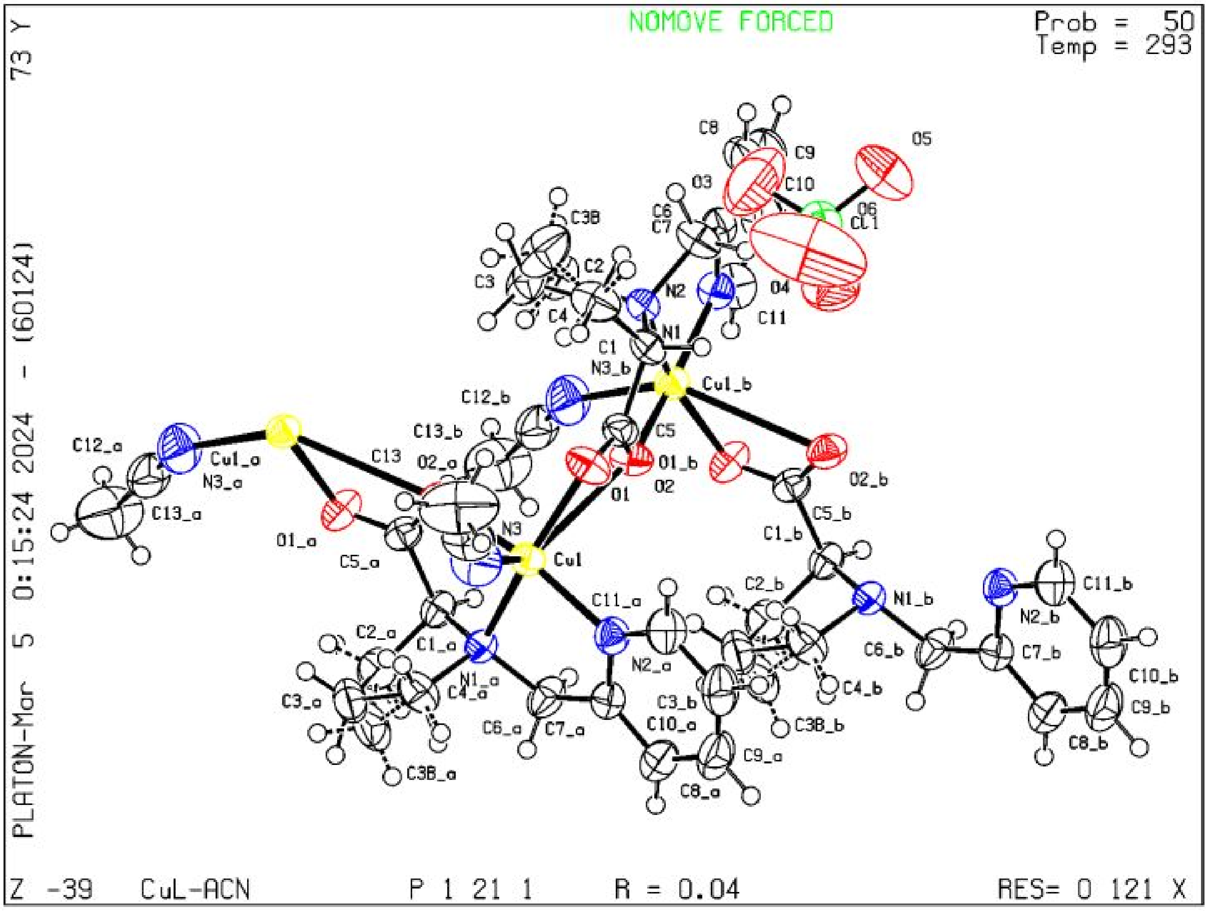


Figure S38. Thermal ellipsoids of complex 4 crystallized in acetonitrile.

X-Ray Crystal Parameters for 4 crystallized in acetonitrile

Table S8 **Crystal data and structure refinement for CuL-ACN.**

| | |
|---|---|
| Identification code | CuL-ACN |
| Empirical formula | $C_{13}H_{16}ClCuN_3O_6$ |
| Formula weight | 409.28 |
| Temperature/K | 293(2) |
| Crystal system | monoclinic |
| Space group | $P2_1$ |

| | |
|---|---|
| a/Å | 11.4151(8) |
| b/Å | 6.9420(3) |
| c/Å | 12.0661(9) |
| α/° | 90 |
| β/° | 118.066(9) |
| γ/° | 90 |
| Volume/Å$^3$ | 843.72(11) |
| Z | 2 |
| $\rho_{calc}$g/cm$^3$ | 1.611 |
| μ/mm$^{-1}$ | 1.487 |
| F(000) | 418.0 |
| Crystal size/mm$^3$ | 0.42 × 0.314 × 0.207 |
| Radiation | Mo Kα (λ = 0.71073) |
| 2Θ range for data collection/° | 6.75 to 68.654 |
| Index ranges | -18 ≤ h ≤ 17, -10 ≤ k ≤ 10, -16 ≤ l ≤ 18 |
| Reflections collected | 11268 |
| Independent reflections | 6022 [$R_{int}$ = 0.0223, $R_{sigma}$ = 0.0375] |
| Data/restraints/parameters | 6022/81/227 |
| Goodness-of-fit on F$^2$ | 1.084 |
| Final R indexes [I>=2σ (I)] | $R_1$ = 0.0388, w$R_2$ = 0.0939 |
| Final R indexes [all data] | $R_1$ = 0.0518, w$R_2$ = 0.1077 |
| Largest diff. peak/hole / e Å$^{-3}$ | 0.39/-0.65 |
| Flack parameter | 0.005(6) |

Table S9 **Fractional Atomic Coordinates (×10$^4$) and Equivalent Isotropic Displacement Parameters (Å$^2$×10$^3$) for CuL-ACN. $U_{eq}$ is defined as 1/3 of the trace of the orthogonalised $U_{IJ}$ tensor.**

| **Atom** | ***x*** | ***y*** | ***z*** | **U(eq)** |
|---|---|---|---|---|
| Cu1 | 5013.6(3) | 2136(3) | 3600.2(3) | 35.48(11) |
| O1 | 6523(3) | 3656(5) | 4838(3) | 46.2(6) |
| O2 | 5097(3) | 5471(5) | 5131(2) | 44.2(5) |
| N1 | 6831(3) | 6163(5) | 7494(3) | 33.3(5) |
| N2 | 5364(3) | 8790(5) | 7871(3) | 39.8(6) |
| N3 | 6490(4) | 138(7) | 3398(4) | 67.0(10) |
| C1 | 7398(3) | 5675(4) | 6640(3) | 36.5(6) |
| C2 | 8481(3) | 4226(3) | 7353(3) | 55.6(10) |
| C3 | 7868(5) | 2989(4) | 7973(6) | 69(3) |

Table S9 Fractional Atomic Coordinates (×10$^4$) and Equivalent Isotropic Displacement Parameters (Å$^2$×10$^3$) for CuL-ACN. $U_{eq}$ is defined as 1/3 of the trace of the orthogonalised $U_{IJ}$ tensor.

| Atom | *x* | *y* | *z* | U(eq) |
|---|---|---|---|---|
| C3B | 8240(5) | 3484(11) | 8405(5) | 80(5) |
| C4 | 6907(3) | 4285(5) | 8152(4) | 53.2(9) |
| C6 | 7558(4) | 7723(6) | 8402(4) | 47.0(8) |
| C7 | 6634(4) | 8780(6) | 8757(3) | 40.2(7) |
| C8 | 7050(4) | 9760(7) | 9866(4) | 51.9(9) |
| C9 | 6120(6) | 10764(7) | 10066(5) | 61.6(12) |
| C10 | 4829(5) | 10780(7) | 9166(5) | 59.4(11) |
| C11 | 4458(4) | 9740(7) | 8074(4) | 51.7(9) |
| C5 | 6252(3) | 4871(6) | 5442(3) | 36.9(6) |
| C12 | 7496(5) | -364(9) | 3622(5) | 63.6(11) |
| C13 | 8836(7) | -990(16) | 3945(7) | 111(3) |
| Cl1 | 10706.9(10) | 9360(4) | 7839.6(11) | 56.4(2) |
| O3 | 10656(8) | 8071(12) | 8718(7) | 169(3) |
| O4 | 9393(4) | 9726(8) | 6925(4) | 83.6(11) |
| O5 | 11401(8) | 11003(10) | 8455(6) | 145(3) |
| O6 | 11376(6) | 8512(18) | 7258(8) | 215(5) |

Table S10 Anisotropic Displacement Parameters (Å$^2$×10$^3$) for CuL-ACN. The Anisotropic displacement factor exponent takes the form: $-2\pi^2[h^2a^{*2}U_{11}+2hka^*b^*U_{12}+\ldots]$.

| Atom | $U_{11}$ | $U_{22}$ | $U_{33}$ | $U_{23}$ | $U_{13}$ | $U_{12}$ |
|---|---|---|---|---|---|---|
| Cu1 | 31.50(18) | 38.62(16) | 34.30(18) | -1.95(17) | 13.80(14) | -1.54(17) |
| O1 | 40.0(13) | 54.2(13) | 47.1(14) | -19.0(11) | 22.7(12) | -9.4(11) |
| O2 | 34.7(12) | 55.2(13) | 37.2(12) | -6.7(11) | 12.4(10) | 1.3(11) |
| N1 | 32.9(13) | 32.8(11) | 33.0(13) | -4.1(9) | 14.7(11) | -3.5(9) |
| N2 | 41.3(15) | 39.2(13) | 42.2(15) | -1.6(11) | 22.4(13) | 3.0(11) |
| N3 | 61(2) | 70(2) | 76(3) | -6(2) | 38(2) | 14(2) |
| C1 | 32.1(15) | 37.9(14) | 40.6(16) | -8.1(12) | 18.1(13) | -7.5(12) |
| C2 | 37(2) | 63(2) | 58(2) | -14.1(19) | 14.6(18) | 8.6(17) |
| C3 | 112(9) | 42(4) | 55(6) | 8(4) | 42(6) | 29(5) |
| C3B | 121(12) | 63(6) | 69(8) | 17(6) | 55(8) | 54(8) |
| C4 | 59(2) | 47.5(17) | 55(2) | 15.7(16) | 28(2) | 8.0(17) |
| C6 | 35.1(18) | 51.9(18) | 51(2) | -18.8(15) | 17.5(17) | -5.3(13) |
| C7 | 43.3(18) | 35.7(14) | 42.9(17) | -2.6(12) | 21.3(16) | -1.6(13) |
| C8 | 59(2) | 47.7(18) | 49(2) | -11.1(16) | 25(2) | -6.4(17) |
| C9 | 89(4) | 48.2(19) | 62(3) | -13.3(18) | 48(3) | -2(2) |

Table S10 Anisotropic Displacement Parameters (Å$^2$×10$^3$) for CuL-ACN. The Anisotropic displacement factor exponent takes the form: $-2\pi^2[h^2a^{*2}U_{11}+2hka^*b^*U_{12}+\ldots]$.

| Atom | $U_{11}$ | $U_{22}$ | $U_{33}$ | $U_{23}$ | $U_{13}$ | $U_{12}$ |
|---|---|---|---|---|---|---|
| C10 | 72(3) | 47.7(19) | 77(3) | -5.4(19) | 50(3) | 7.2(19) |
| C11 | 48(2) | 52.8(19) | 64(2) | -2.6(18) | 35(2) | 3.9(17) |
| C5 | 36.7(16) | 40.6(14) | 33.8(15) | -3.1(11) | 16.9(14) | -7.9(12) |
| C12 | 61(3) | 73(3) | 58(3) | -12(2) | 29(2) | 14(2) |
| C13 | 66(4) | 176(9) | 88(5) | -12(5) | 33(4) | 43(5) |
| Cl1 | 44.4(5) | 55.5(5) | 62.7(6) | 0.0(4) | 19.9(5) | 0.4(4) |
| O3 | 121(5) | 174(7) | 147(6) | 91(5) | 11(4) | -30(5) |
| O4 | 60(2) | 95(3) | 76(3) | 2(2) | 16.2(19) | 15(2) |
| O5 | 150(6) | 106(4) | 114(4) | -24(3) | 9(4) | -59(4) |
| O6 | 80(4) | 353(13) | 209(8) | -134(9) | 67(5) | 14(6) |

Table S11 Bond Lengths for CuL-ACN.

| Atom | Atom | Length/Å | Atom | Atom | Length/Å |
|---|---|---|---|---|---|
| Cu1 | O1 | 1.973(3) | C2 | C3 | 1.5100(3) |
| Cu1 | O2[1] | 1.969(3) | C2 | C3B | 1.5100(3) |
| Cu1 | N1[1] | 2.005(3) | C3 | C4 | 1.5100(3) |
| Cu1 | N2[1] | 1.983(3) | C3B | C4 | 1.5100(3) |
| Cu1 | N3 | 2.283(4) | C6 | C7 | 1.503(5) |
| O1 | C5 | 1.245(4) | C7 | C8 | 1.371(5) |
| O2 | C5 | 1.259(4) | C8 | C9 | 1.382(6) |
| N1 | C1 | 1.491(4) | C9 | C10 | 1.358(7) |
| N1 | C4 | 1.508(4) | C10 | C11 | 1.383(6) |
| N1 | C6 | 1.486(4) | C12 | C13 | 1.456(7) |
| N2 | C7 | 1.337(5) | Cl1 | O3 | 1.410(6) |
| N2 | C11 | 1.343(4) | Cl1 | O4 | 1.405(4) |
| N3 | C12 | 1.104(6) | Cl1 | O5 | 1.387(5) |
| C1 | C2 | 1.5100(3) | Cl1 | O6 | 1.388(6) |
| C1 | C5 | 1.527(4) | | | |

[1]1-X,-1/2+Y,1-Z

Table S12 Bond Angles for CuL-ACN.

| Atom | Atom | Atom | Angle/˚ | Atom | Atom | Atom | Angle/˚ |
|---|---|---|---|---|---|---|---|
| O1 | Cu1 | N1[1] | 162.12(10) | C3 | C2 | C1 | 102.4(3) |

Table S12 **Bond Angles for CuL-ACN.**

| Atom | Atom | Atom | Angle/˚ | Atom | Atom | Atom | Angle/˚ |
|---|---|---|---|---|---|---|---|
| O1 | Cu1 | N2[1] | 96.45(13) | C3B | C2 | C1 | 106.4(3) |
| O1 | Cu1 | N3 | 88.86(14) | C4 | C3 | C2 | 105.6(3) |
| O2[1] | Cu1 | O1 | 94.49(11) | C4 | C3B | C2 | 105.6(3) |
| O2[1] | Cu1 | N1[1] | 83.22(11) | N1 | C4 | C3 | 107.8(3) |
| O2[1] | Cu1 | N2[1] | 165.69(12) | N1 | C4 | C3B | 103.2(3) |
| O2[1] | Cu1 | N3 | 92.70(14) | N1 | C6 | C7 | 110.1(3) |
| N1[1] | Cu1 | N3 | 108.93(14) | N2 | C7 | C6 | 114.6(3) |
| N2[1] | Cu1 | N1[1] | 83.53(12) | N2 | C7 | C8 | 121.7(3) |
| N2[1] | Cu1 | N3 | 96.70(14) | C8 | C7 | C6 | 123.6(4) |
| C5 | O1 | Cu1 | 115.8(2) | C7 | C8 | C9 | 118.6(4) |
| C5 | O2 | Cu1[2] | 113.8(2) | C10 | C9 | C8 | 119.9(4) |
| C1 | N1 | Cu1[2] | 106.54(18) | C9 | C10 | C11 | 119.2(4) |
| C1 | N1 | C4 | 103.4(2) | N2 | C11 | C10 | 120.9(4) |
| C4 | N1 | Cu1[2] | 114.9(2) | O1 | C5 | O2 | 124.3(3) |
| C6 | N1 | Cu1[2] | 106.8(2) | O1 | C5 | C1 | 117.6(3) |
| C6 | N1 | C1 | 113.7(2) | O2 | C5 | C1 | 118.1(3) |
| C6 | N1 | C4 | 111.6(3) | N3 | C12 | C13 | 178.5(6) |
| C7 | N2 | Cu1[2] | 114.2(2) | O4 | Cl1 | O3 | 107.4(4) |
| C7 | N2 | C11 | 119.7(3) | O5 | Cl1 | O3 | 109.5(4) |
| C11 | N2 | Cu1[2] | 125.8(3) | O5 | Cl1 | O4 | 113.7(4) |
| C12 | N3 | Cu1 | 154.1(4) | O5 | Cl1 | O6 | 107.9(5) |
| N1 | C1 | C2 | 105.2(2) | O6 | Cl1 | O3 | 109.8(7) |
| N1 | C1 | C5 | 106.3(2) | O6 | Cl1 | O4 | 108.5(4) |
| C2 | C1 | C5 | 113.8(3) | | | | |

[1]1-X,-1/2+Y,1-Z; [2]1-X,1/2+Y,1-Z

Table S11 **Torsion Angles for CuL-ACN.**

| A | B | C | D | Angle/˚ | A | B | C | D | Angle/˚ |
|---|---|---|---|---|---|---|---|---|---|
| Cu1 | O1 | C5 | O2 | -16.5(5) | C1 | C2 | C3 | C4 | -28.3(5) |
| Cu1 | O1 | C5 | C1 | 163.4(2) | C1 | C2 | C3B | C4 | 8.7(7) |
| Cu1[1] | O2 | C5 | O1 | 172.7(3) | C2 | C1 | C5 | O1 | -34.8(4) |
| Cu1[1] | O2 | C5 | C1 | -7.1(4) | C2 | C1 | C5 | O2 | 145.1(3) |
| Cu1[1] | N1 | C1 | C2 | -156.8(2) | C2 | C3 | C4 | N1 | 7.3(5) |
| Cu1[1] | N1 | C1 | C5 | -35.8(2) | C2 | C3B | C4 | N1 | -30.1(6) |
| Cu1[1] | N1 | C4 | C3 | 132.8(4) | C4 | N1 | C1 | C2 | -35.3(3) |

Table S11 Torsion Angles for CuL-ACN.

| A | B | C | D | Angle/˚ | A | B | C | D | Angle/˚ |
|---|---|---|---|---|---|---|---|---|---|
| Cu1[1] | N1 | C4 | C3B | 156.1(4) | C4 | N1 | C1 | C5 | 85.6(3) |
| Cu1[1] | N1 | C6 | C7 | 35.0(4) | C4 | N1 | C6 | C7 | -91.3(4) |
| Cu1[1] | N2 | C7 | C6 | 7.3(4) | C6 | N1 | C1 | C2 | 85.9(3) |
| Cu1[1] | N2 | C7 | C8 | -176.3(3) | C6 | N1 | C1 | C5 | -153.2(3) |
| Cu1[1] | N2 | C11 | C10 | 176.8(3) | C6 | N1 | C4 | C3 | -105.5(4) |
| N1 | C1 | C2 | C3 | 39.9(4) | C6 | N1 | C4 | C3B | -82.1(4) |
| N1 | C1 | C2 | C3B | 16.5(5) | C6 | C7 | C8 | C9 | 176.7(4) |
| N1 | C1 | C5 | O1 | -150.1(3) | C7 | N2 | C11 | C10 | 2.9(6) |
| N1 | C1 | C5 | O2 | 29.8(4) | C7 | C8 | C9 | C10 | -0.7(7) |
| N1 | C6 | C7 | N2 | -28.8(4) | C8 | C9 | C10 | C11 | 1.8(7) |
| N1 | C6 | C7 | C8 | 154.9(3) | C9 | C10 | C11 | N2 | -3.0(6) |
| N2 | C7 | C8 | C9 | 0.7(6) | C11 | N2 | C7 | C6 | -178.1(3) |
| C1 | N1 | C4 | C3 | 17.1(4) | C11 | N2 | C7 | C8 | -1.7(5) |
| C1 | N1 | C4 | C3B | 40.5(4) | C5 | C1 | C2 | C3 | -76.0(4) |
| C1 | N1 | C6 | C7 | 152.2(3) | C5 | C1 | C2 | C3B | -99.4(5) |

[1]1-X,1/2+Y,1-Z

Table S13 Hydrogen Atom Coordinates (Å×10$^4$) and Isotropic Displacement Parameters (Å$^2$×10$^3$) for CuL-ACN.

| Atom | *x* | *y* | *z* | U(eq) |
|---|---|---|---|---|
| H1 | 7767.72 | 6827.57 | 6450.9 | 44 |
| H2AA | 8687.62 | 3473.71 | 6789.79 | 67 |
| H2AB | 9282.71 | 4852.62 | 7971.77 | 67 |
| H2BC | 8443.55 | 3178.11 | 6805.5 | 67 |
| H2BD | 9346.92 | 4832.03 | 7688.82 | 67 |
| H3A | 7405.38 | 1900.49 | 7443.25 | 83 |
| H3B | 8544.37 | 2510.5 | 8775.27 | 83 |
| H3BA | 8225.71 | 2087.28 | 8405.88 | 96 |
| H3BB | 8930.81 | 3926.45 | 9211.47 | 96 |
| H4AA | 6037.7 | 3688.16 | 7799.01 | 64 |
| H4AB | 7214.11 | 4509.32 | 9039.68 | 64 |
| H4BC | 6193.01 | 3425.64 | 7619.5 | 64 |
| H4BD | 6868.47 | 4496.98 | 8928.72 | 64 |
| H6A | 7925.33 | 8615.46 | 8028.23 | 56 |
| H6B | 8285.67 | 7176.52 | 9147.48 | 56 |
| H8 | 7938.81 | 9750.36 | 10470.42 | 62 |

Table S13 **Hydrogen Atom Coordinates (Å×10⁴) and Isotropic Displacement Parameters (Å²×10³) for CuL-ACN.**

| Atom | *x* | *y* | *z* | U(eq) |
|---|---|---|---|---|
| H9 | 6378.09 | 11427.47 | 10814.53 | 74 |
| H10 | 4202.15 | 11481.89 | 9283.1 | 71 |
| H11 | 3568.74 | 9697.41 | 7471.62 | 62 |
| H13A | 8994.7 | -792.49 | 3239.43 | 167 |
| H13B | 8931.42 | -2332.46 | 4160.07 | 167 |
| H13C | 9465.37 | -256.47 | 4648.31 | 167 |

Table S14 **Atomic Occupancy for CuL-ACN.**

| Atom | *Occupancy* | Atom | *Occupancy* | Atom | *Occupancy* |
|---|---|---|---|---|---|
| H2AA | 0.5384 | H2AB | 0.5384 | H2BC | 0.4616 |
| H2BD | 0.4616 | C3 | 0.5384 | H3A | 0.5384 |
| H3B | 0.5384 | C3B | 0.4616 | H3BA | 0.4616 |
| H3BB | 0.4616 | H4AA | 0.5384 | H4AB | 0.5384 |
| H4BC | 0.4616 | H4BD | 0.4616 | | |

**CuL-ACN**

Table S15 **Crystal data and structure refinement for CuL-ACN.**

| | |
|---|---|
| Identification code | CuL-ACN |
| Empirical formula | $C_{13}H_{16}ClCuN_3O_6$ |
| Formula weight | 409.28 |
| Temperature/K | 293(2) |
| Crystal system | monoclinic |
| Space group | $P2_1$ |
| a/Å | 11.4151(8) |
| b/Å | 6.9420(3) |
| c/Å | 12.0661(9) |
| α/° | 90 |
| β/° | 118.066(9) |
| γ/° | 90 |
| Volume/Å³ | 843.72(11) |
| Z | 2 |
| $\rho_{calc}$g/cm³ | 1.611 |
| μ/mm⁻¹ | 1.487 |
| F(000) | 418.0 |

| | |
|---|---|
| Crystal size/mm$^3$ | 0.42 × 0.314 × 0.207 |
| Radiation | Mo Kα (λ = 0.71073) |
| 2Θ range for data collection/° | 6.75 to 68.654 |
| Index ranges | -18 ≤ h ≤ 17, -10 ≤ k ≤ 10, -16 ≤ l ≤ 18 |
| Reflections collected | 11268 |
| Independent reflections | 6022 [$R_{int}$ = 0.0223, $R_{sigma}$ = 0.0375] |
| Data/restraints/parameters | 6022/81/227 |
| Goodness-of-fit on $F^2$ | 1.084 |
| Final R indexes [I>=2σ (I)] | $R_1$ = 0.0388, $wR_2$ = 0.0939 |
| Final R indexes [all data] | $R_1$ = 0.0518, $wR_2$ = 0.1077 |
| Largest diff. peak/hole / e Å$^{-3}$ | 0.39/-0.65 |
| Flack parameter | 0.005(6) |

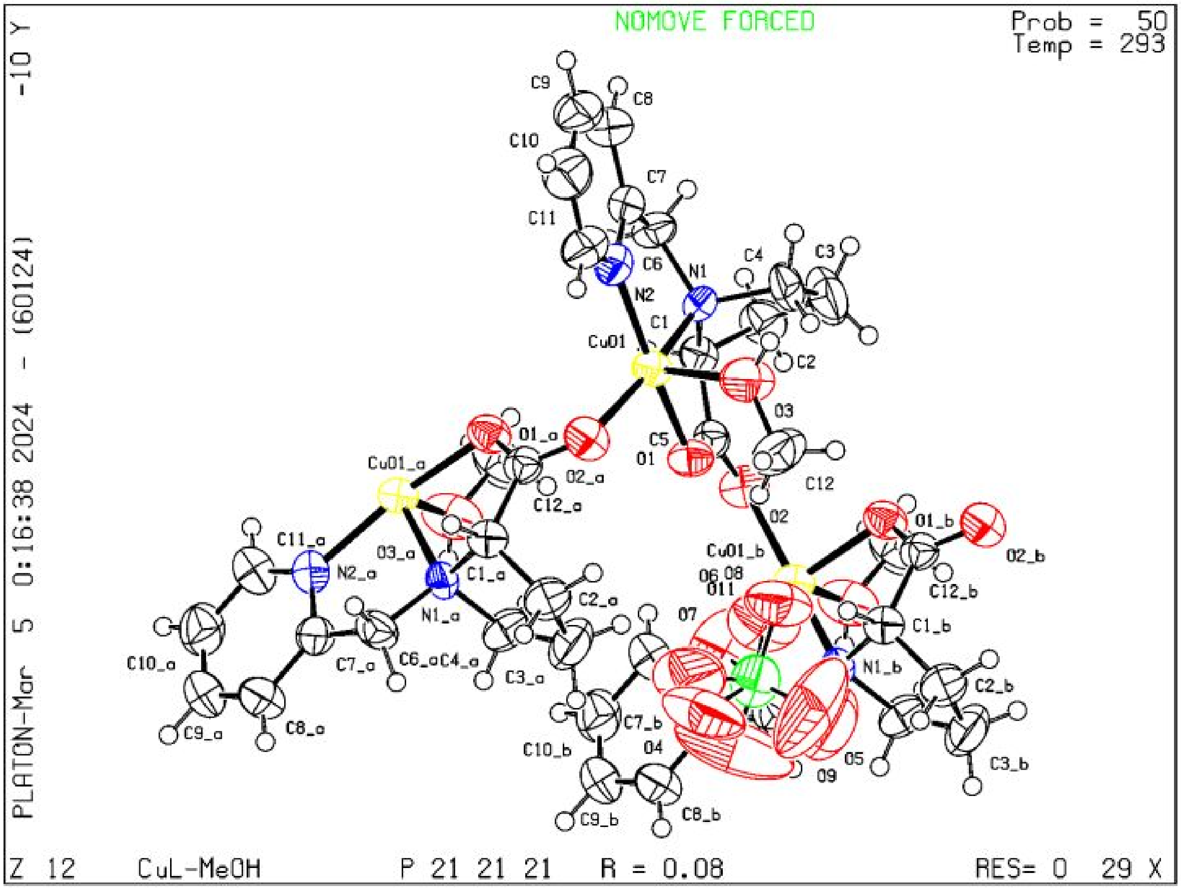


Figure S39. Thermal ellipsoids of complex 4 crystallized in methanol.

X-Ray Crystal Parameters for 4 crystallized in methanol

Table S16 **Crystal data and structure refinement for CuL-MeOH.**

| | |
|---|---|
| Identification code | CuL-MeOH |
| Empirical formula | $C_{12}H_{17}ClCuN_2O_7$ |

| | |
|---|---|
| Formula weight | 400.26 |
| Temperature/K | 293(2) |
| Crystal system | orthorhombic |
| Space group | $P2_12_12_1$ |
| a/Å | 6.9604(8) |
| b/Å | 12.3739(13) |
| c/Å | 18.413(3) |
| α/° | 90 |
| β/° | 90 |
| γ/° | 90 |
| Volume/Å$^3$ | 1585.8(3) |
| Z | 4 |
| $\rho_{calc}$g/cm$^3$ | 1.676 |
| μ/mm$^{-1}$ | 1.582 |
| F(000) | 820.0 |
| Crystal size/mm$^3$ | 0.373 × 0.147 × 0.06 |
| Radiation | Mo Kα (λ = 0.71073) |
| 2Θ range for data collection/° | 5.516 to 51.472 |
| Index ranges | -8 ≤ h ≤ 8, -15 ≤ k ≤ 15, -18 ≤ l ≤ 22 |
| Reflections collected | 14857 |
| Independent reflections | 3037 [$R_{int}$ = 0.0733, $R_{sigma}$ = 0.0531] |
| Data/restraints/parameters | 3037/101/248 |
| Goodness-of-fit on $F^2$ | 1.092 |
| Final R indexes [I>=2σ (I)] | $R_1$ = 0.0765, $wR_2$ = 0.2005 |
| Final R indexes [all data] | $R_1$ = 0.0888, $wR_2$ = 0.2185 |
| Largest diff. peak/hole / e Å$^{-3}$ | 1.61/-0.48 |
| Flack parameter | -0.005(15) |

Table S17 **Fractional Atomic Coordinates (×10$^4$) and Equivalent Isotropic Displacement Parameters (Å$^2$×10$^3$) for CuL-MeOH. $U_{eq}$ is defined as 1/3 of the trace of the orthogonalised $U_{IJ}$ tensor.**

| Atom | *x* | *y* | *z* | U(eq) |
|---|---|---|---|---|
| Cu01 | 2688.8(17) | 8219.1(9) | 5806.3(6) | 43.0(4) |
| O1 | 4320(12) | 7625(6) | 5028(4) | 49.1(17) |
| O2 | 6230(11) | 8137(6) | 4117(4) | 52.8(18) |
| O3 | 4568(19) | 7616(8) | 6687(6) | 77(3) |
| N1 | 3847(11) | 9605(7) | 5523(5) | 42.3(18) |

Table S17 Fractional Atomic Coordinates (×10[4]) and Equivalent Isotropic Displacement Parameters ($Å^2×10^3$) for CuL-MeOH. $U_{eq}$ is defined as 1/3 of the trace of the orthogonalised $U_{IJ}$ tensor.

| Atom | *x* | *y* | *z* | U(eq) |
|---|---|---|---|---|
| N2 | 1103(13) | 9143(8) | 6443(5) | 47(2) |
| C1 | 4404(8) | 9496(4) | 4718(5) | 42(2) |
| C2 | 5972(8) | 10319(6) | 4595(3) | 59(3) |
| C3 | 7068(9) | 10334(10) | 5303(3) | 87(5) |
| C4 | 5739(11) | 9864(8) | 5869(4) | 60(3) |
| C5 | 5015(13) | 8335(4) | 4616(5) | 42(2) |
| C6 | 2446(13) | 10480(8) | 5643(4) | 52(2) |
| C7 | 1318(12) | 10206(9) | 6319(4) | 49(3) |
| C8 | 420(20) | 10971(12) | 6756(8) | 69(4) |
| C9 | -680(20) | 10625(12) | 7319(9) | 73(4) |
| C10 | -860(20) | 9542(14) | 7444(8) | 74(4) |
| C11 | 21(19) | 8831(12) | 7010(7) | 62(3) |
| C12 | 5550(20) | 6626(12) | 6677(10) | 84(5) |
| Cl1 | 5756(5) | 3272(3) | 6416(2) | 72.4(9) |
| O8 | 5740(40) | 3969(17) | 7079(11) | 100(8) |
| O9 | 7730(30) | 2960(30) | 6310(20) | 167(15) |
| O10 | 4470(50) | 2430(20) | 6620(20) | 147(16) |
| O11 | 5190(50) | 4040(30) | 5871(16) | 156(16) |
| O4 | 5840(80) | 2810(30) | 5670(15) | 210(30) |
| O5 | 7000(60) | 2740(40) | 6768(19) | 210(30) |
| O6 | 5850(50) | 4270(20) | 6350(20) | 145(15) |
| O7 | 4020(50) | 2960(20) | 6600(30) | 170(20) |

Table S18 Anisotropic Displacement Parameters ($Å^2×10^3$) for CuL-MeOH. The Anisotropic displacement factor exponent takes the form: $-2\pi^2[h^2a^{*2}U_{11}+2hka^*b^*U_{12}+\ldots]$.

| Atom | $U_{11}$ | $U_{22}$ | $U_{33}$ | $U_{23}$ | $U_{13}$ | $U_{12}$ |
|---|---|---|---|---|---|---|
| Cu01 | 49.5(6) | 37.7(6) | 41.7(7) | -3.0(5) | -1.4(5) | 0.2(5) |
| O1 | 60(5) | 37(4) | 50(4) | 1(3) | 6(4) | 2(3) |
| O2 | 67(4) | 47(4) | 44(4) | 9(3) | 13(3) | 1(3) |
| O3 | 105(8) | 65(6) | 62(6) | -8(4) | -31(5) | 24(6) |
| N1 | 32(4) | 45(4) | 49(5) | -8(4) | 0(3) | 7(3) |
| N2 | 41(4) | 54(5) | 45(5) | 2(4) | 2(4) | 3(4) |
| C1 | 47(5) | 41(5) | 36(5) | 3(4) | -5(4) | 1(4) |
| C2 | 65(7) | 44(6) | 68(8) | 8(6) | 5(6) | -8(6) |
| C3 | 54(7) | 127(13) | 81(10) | -32(9) | 7(7) | -34(9) |

Table S18 Anisotropic Displacement Parameters (Å$^2$×10$^3$) for CuL-MeOH. The Anisotropic displacement factor exponent takes the form: $-2\pi^2[h^2a^{*2}U_{11}+2hka^*b^*U_{12}+\ldots]$.

| Atom | $U_{11}$ | $U_{22}$ | $U_{33}$ | $U_{23}$ | $U_{13}$ | $U_{12}$ |
|---|---|---|---|---|---|---|
| C4 | 48(5) | 72(8) | 58(7) | -8(6) | -21(5) | -9(5) |
| C5 | 41(5) | 43(5) | 42(5) | -1(4) | -7(4) | 0(4) |
| C6 | 56(6) | 39(5) | 61(6) | -3(4) | 1(5) | 11(5) |
| C7 | 41(5) | 47(6) | 58(7) | -6(5) | -7(4) | 4(4) |
| C8 | 68(8) | 59(7) | 80(9) | -20(7) | 1(7) | 9(6) |
| C9 | 62(7) | 78(9) | 78(9) | -24(7) | 22(7) | 3(7) |
| C10 | 67(8) | 91(11) | 64(9) | -3(7) | 18(7) | -9(8) |
| C11 | 61(7) | 64(8) | 60(8) | -3(6) | 6(6) | 13(6) |
| C12 | 84(10) | 76(10) | 93(11) | -4(8) | -4(8) | 35(9) |
| Cl1 | 70.4(18) | 62.9(19) | 84(2) | 20.5(18) | 0.9(15) | -1.3(17) |
| O8 | 150(20) | 58(12) | 90(14) | -9(11) | -21(15) | 11(13) |
| O9 | 92(18) | 240(40) | 170(40) | -50(30) | -1(18) | 44(19) |
| O10 | 200(30) | 90(20) | 150(30) | -48(18) | 50(30) | -60(30) |
| O11 | 150(30) | 230(40) | 86(17) | 70(20) | 20(20) | 30(30) |
| O4 | 450(90) | 110(20) | 61(18) | -14(15) | 30(30) | -70(40) |
| O5 | 150(30) | 390(70) | 100(30) | 30(30) | -30(20) | 150(40) |
| O6 | 150(30) | 51(13) | 230(50) | 20(20) | 20(30) | -22(15) |
| O7 | 120(20) | 80(20) | 300(60) | -40(30) | 80(30) | -50(20) |

Table S19 Bond Lengths for CuL-MeOH.

| Atom | Atom | Length/Å | Atom | Atom | Length/Å |
|---|---|---|---|---|---|
| Cu01 | O1 | 1.971(8) | C2 | C3 | 1.5100(3) |
| Cu01 | O2[1] | 1.967(8) | C3 | C4 | 1.5100(2) |
| Cu01 | O3 | 2.213(9) | C6 | C7 | 1.5100(3) |
| Cu01 | N1 | 1.966(9) | C7 | C8 | 1.390(16) |
| Cu01 | N2 | 1.975(9) | C8 | C9 | 1.36(2) |
| O1 | C5 | 1.257(12) | C9 | C10 | 1.37(2) |
| O2 | C5 | 1.272(12) | C10 | C11 | 1.34(2) |
| O3 | C12 | 1.404(16) | Cl1 | O8 | 1.495(15) |
| N1 | C1 | 1.539(12) | Cl1 | O9 | 1.445(16) |
| N1 | C4 | 1.497(11) | Cl1 | O10 | 1.419(17) |
| N1 | C6 | 1.473(12) | Cl1 | O11 | 1.437(16) |
| N2 | C7 | 1.343(13) | Cl1 | O4 | 1.49(3) |
| N2 | C11 | 1.344(16) | Cl1 | O5 | 1.26(3) |
| C1 | C2 | 1.5100(2) | Cl1 | O6 | 1.24(2) |
| C1 | C5 | 1.5100(3) | Cl1 | O7 | 1.31(3) |

[1]-1/2+X,3/2-Y,1-Z

Table S20 Bond Angles for CuL-MeOH.

| Atom | Atom | Atom | Angle/˚ | Atom | Atom | Atom | Angle/˚ |
|---|---|---|---|---|---|---|---|
| O1 | Cu01 | O3 | 93.8(4) | C2 | C3 | C4 | 106.4(5) |
| O1 | Cu01 | N2 | 165.9(3) | N1 | C4 | C3 | 109.1(6) |
| O2[1] | Cu01 | O1 | 91.8(3) | O1 | C5 | O2 | 123.8(6) |
| O2[1] | Cu01 | O3 | 88.0(4) | O1 | C5 | C1 | 118.8(8) |
| O2[1] | Cu01 | N2 | 99.4(4) | O2 | C5 | C1 | 117.4(7) |
| N1 | Cu01 | O1 | 84.1(3) | N1 | C6 | C7 | 107.6(7) |
| N1 | Cu01 | O2[1] | 167.3(3) | N2 | C7 | C6 | 114.7(8) |
| N1 | Cu01 | O3 | 104.2(4) | N2 | C7 | C8 | 121.2(10) |
| N1 | Cu01 | N2 | 83.2(4) | C8 | C7 | C6 | 123.9(11) |
| N2 | Cu01 | O3 | 95.2(4) | C9 | C8 | C7 | 118.7(13) |
| C5 | O1 | Cu01 | 113.6(6) | C8 | C9 | C10 | 119.4(12) |
| C5 | O2 | Cu01[2] | 117.1(5) | C11 | C10 | C9 | 120.0(14) |
| C12 | O3 | Cu01 | 124.9(9) | C10 | C11 | N2 | 122.2(14) |
| C1 | N1 | Cu01 | 106.4(5) | O9 | Cl1 | O8 | 105.7(18) |
| C4 | N1 | Cu01 | 115.8(6) | O10 | Cl1 | O8 | 101.7(16) |
| C4 | N1 | C1 | 101.9(6) | O10 | Cl1 | O9 | 116(2) |
| C6 | N1 | Cu01 | 109.3(6) | O10 | Cl1 | O11 | 120(2) |
| C6 | N1 | C1 | 112.0(7) | O11 | Cl1 | O8 | 100.8(17) |
| C6 | N1 | C4 | 111.2(8) | O11 | Cl1 | O9 | 110(2) |
| C7 | N2 | Cu01 | 113.8(6) | O5 | Cl1 | O4 | 104(2) |
| C7 | N2 | C11 | 118.4(9) | O5 | Cl1 | O7 | 110(2) |
| C11 | N2 | Cu01 | 127.5(8) | O6 | Cl1 | O4 | 106(2) |
| C2 | C1 | N1 | 105.5(6) | O6 | Cl1 | O5 | 122(3) |
| C2 | C1 | C5 | 114.8(6) | O6 | Cl1 | O7 | 112(2) |
| C5 | C1 | N1 | 105.9(7) | O7 | Cl1 | O4 | 100(2) |
| C3 | C2 | C1 | 104.1(6) | | | | |

[1]-1/2+X,3/2-Y,1-Z; [2]1/2+X,3/2-Y,1-Z

Table S21 Torsion Angles for CuL-MeOH.

| A | B | C | D | Angle/˚ | A | B | C | D | Angle/˚ |
|---|---|---|---|---|---|---|---|---|---|
| Cu01 | O1 | C5 | O2 | 173.9(8) | C1 | C2 | C3 | C4 | -20.0(10) |
| Cu01 | O1 | C5 | C1 | -5.2(11) | C2 | C1 | C5 | O1 | 143.2(8) |

Table S21 Torsion Angles for CuL-MeOH.

| A | B | C | D | Angle/˚ | A | B | C | D | Angle/˚ |
|---|---|---|---|---|---|---|---|---|---|
| Cu01[1] | O2 | C5 | O1 | -20.0(13) | C2 | C1 | C5 | O2 | -36.0(12) |
| Cu01[1] | O2 | C5 | C1 | 159.2(6) | C2 | C3 | C4 | N1 | -2.0(11) |
| Cu01 | N1 | C1 | C2 | -156.8(4) | C4 | N1 | C1 | C2 | -35.0(8) |
| Cu01 | N1 | C1 | C5 | -34.6(7) | C4 | N1 | C1 | C5 | 87.1(8) |
| Cu01 | N1 | C4 | C3 | 137.5(7) | C4 | N1 | C6 | C7 | -93.0(9) |
| Cu01 | N1 | C6 | C7 | 36.1(9) | C5 | C1 | C2 | C3 | -81.8(9) |
| Cu01 | N2 | C7 | C6 | 10.0(11) | C6 | N1 | C1 | C2 | 83.9(8) |
| Cu01 | N2 | C7 | C8 | -174.9(9) | C6 | N1 | C1 | C5 | -153.9(7) |
| Cu01 | N2 | C11 | C10 | 174.0(11) | C6 | N1 | C4 | C3 | -97.0(9) |
| N1 | C1 | C2 | C3 | 34.4(8) | C6 | C7 | C8 | C9 | 175.0(11) |
| N1 | C1 | C5 | O1 | 27.3(10) | C7 | N2 | C11 | C10 | 1.0(19) |
| N1 | C1 | C5 | O2 | -152.0(8) | C7 | C8 | C9 | C10 | 0(2) |
| N1 | C6 | C7 | N2 | -30.5(11) | C8 | C9 | C10 | C11 | 0(2) |
| N1 | C6 | C7 | C8 | 154.5(10) | C9 | C10 | C11 | N2 | 0(2) |
| N2 | C7 | C8 | C9 | 0.2(19) | C11 | N2 | C7 | C6 | -176.1(9) |
| C1 | N1 | C4 | C3 | 22.5(10) | C11 | N2 | C7 | C8 | -1.0(16) |
| C1 | N1 | C6 | C7 | 153.7(7) | | | | | |

[1]1/2+X,3/2-Y,1-Z

Table S22 Hydrogen Atom Coordinates (Å×10$^4$) and Isotropic Displacement Parameters (Å$^2$×10$^3$) for CuL-MeOH.

| Atom | *x* | *y* | *z* | U(eq) |
|---|---|---|---|---|
| H3 | 4800(200) | 7950(80) | 7080(50) | 116 |
| H1 | 3295.27 | 9658.28 | 4408.2 | 50 |
| H2A | 6798.6 | 10104.55 | 4195.84 | 71 |
| H2B | 5433.01 | 11024.93 | 4489.55 | 71 |
| H3A | 7425.09 | 11067.14 | 5430.12 | 105 |
| H3B | 8227.17 | 9902.34 | 5263.3 | 105 |
| H4A | 6299.32 | 9213.52 | 6072.28 | 71 |
| H4B | 5555.14 | 10380.51 | 6259.33 | 71 |
| H6A | 1590.69 | 10539.13 | 5228.96 | 63 |
| H6B | 3106.42 | 11163.77 | 5704.88 | 63 |
| H8 | 577.36 | 11704.92 | 6665.27 | 82 |
| H9 | -1295.55 | 11122 | 7617.3 | 87 |
| H10 | -1608.21 | 9298.28 | 7830.65 | 89 |
| H11 | -117.2 | 8096.73 | 7104.07 | 74 |

Table S22 **Hydrogen Atom Coordinates (Å×10[4]) and Isotropic Displacement Parameters (Å[2]×10[3]) for CuL-MeOH.**

| **Atom** | *x* | *y* | *z* | **U(eq)** |
|---|---|---|---|---|
| H12A | 5460.29 | 6312.16 | 6201.87 | 127 |
| H12B | 4987.58 | 6145.08 | 7026.71 | 127 |
| H12C | 6877 | 6743.82 | 6796.8 | 127 |

Table S23 **Atomic Occupancy for CuL-MeOH.**

| **Atom** | ***Occupancy*** | **Atom** | ***Occupancy*** | **Atom** | ***Occupancy*** |
|---|---|---|---|---|---|
| O8 | 0.5 | O9 | 0.5 | O10 | 0.5 |
| O11 | 0.5 | O4 | 0.5 | O5 | 0.5 |
| O6 | 0.5 | O7 | 0.5 | | |

**CuL-MeOH**

Table S24 **Crystal data and structure refinement for CuL-MeOH.**

| | |
|---|---|
| Identification code | CuL-MeOH |
| Empirical formula | $C_{12}H_{17}ClCuN_2O_7$ |
| Formula weight | 400.26 |
| Temperature/K | 293(2) |
| Crystal system | orthorhombic |
| Space group | $P2_12_12_1$ |
| a/Å | 6.9604(8) |
| b/Å | 12.3739(13) |
| c/Å | 18.413(3) |
| α/° | 90 |
| β/° | 90 |
| γ/° | 90 |
| Volume/Å[3] | 1585.8(3) |
| Z | 4 |
| $\rho_{calc}$g/cm[3] | 1.676 |
| μ/mm[-1] | 1.582 |
| F(000) | 820.0 |
| Crystal size/mm[3] | 0.373 × 0.147 × 0.06 |
| Radiation | Mo Kα (λ = 0.71073) |
| 2Θ range for data collection/° | 5.516 to 51.472 |
| Index ranges | -8 ≤ h ≤ 8, -15 ≤ k ≤ 15, -18 ≤ l ≤ 22 |

| | |
|---|---|
| Reflections collected | 14857 |
| Independent reflections | 3037 [$R_{int}$ = 0.0733, $R_{sigma}$ = 0.0531] |
| Data/restraints/parameters | 3037/101/248 |
| Goodness-of-fit on $F^2$ | 1.092 |
| Final R indexes [I>=2σ (I)] | $R_1$ = 0.0765, $wR_2$ = 0.2005 |
| Final R indexes [all data] | $R_1$ = 0.0888, $wR_2$ = 0.2185 |
| Largest diff. peak/hole / e Å$^{-3}$ | 1.61/-0.48 |
| Flack parameter | -0.005(15) |

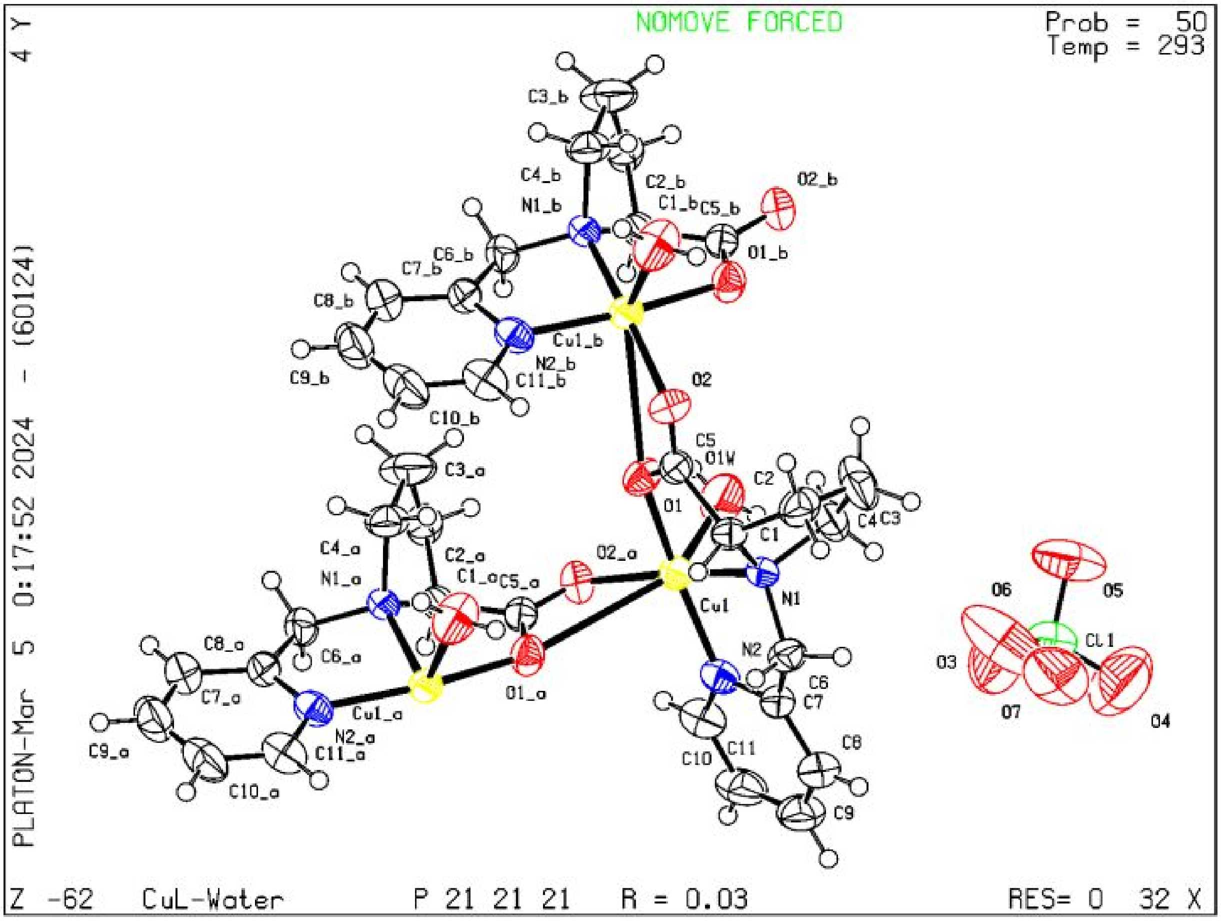


Figure S40. Thermal ellipsoids of complex 4 crystallized in water.

X-Ray Crystal Parameters for 4 crystallized in water

Table S25 **Crystal data and structure refinement for CuL-water.**

| | |
|---|---|
| Identification code | CuL-water |
| Empirical formula | $C_{11}H_{15}ClCuN_2O_7$ |
| Formula weight | 386.24 |

| | |
|---|---|
| Temperature/K | 293(2) |
| Crystal system | orthorhombic |
| Space group | $P2_12_12_1$ |
| a/Å | 7.1965(8) |
| b/Å | 10.9293(13) |
| c/Å | 18.810(2) |
| α/° | 90 |
| β/° | 90 |
| γ/° | 90 |
| Volume/Å$^3$ | 1479.5(3) |
| Z | 4 |
| $\rho_{calc}$g/cm$^3$ | 1.734 |
| μ/mm$^{-1}$ | 1.693 |
| F(000) | 788.0 |
| Crystal size/mm$^3$ | 0.239 × 0.116 × 0.066 |
| Radiation | Mo Kα (λ = 0.71073) |
| 2Θ range for data collection/° | 5.714 to 51.49 |
| Index ranges | -8 ≤ h ≤ 8, -13 ≤ k ≤ 12, -22 ≤ l ≤ 22 |
| Reflections collected | 9103 |
| Independent reflections | 2813 [$R_{int}$ = 0.0333, $R_{sigma}$ = 0.0306] |
| Data/restraints/parameters | 2813/85/214 |
| Goodness-of-fit on $F^2$ | 1.104 |
| Final R indexes [I>=2σ (I)] | $R_1$ = 0.0331, $wR_2$ = 0.0811 |
| Final R indexes [all data] | $R_1$ = 0.0386, $wR_2$ = 0.0862 |

Largest diff. peak/hole / e Å$^{-3}$ 0.41/-0.30

Flack parameter -0.010(8)

Table S26 **Fractional Atomic Coordinates (×10$^4$) and Equivalent Isotropic Displacement Parameters (Å$^2$×10$^3$) for CuL-water. $U_{eq}$ is defined as 1/3 of the trace of the orthogonalised $U_{IJ}$ tensor.**

| Atom | *x* | *y* | *z* | U(eq) |
|---|---|---|---|---|
| Cu1 | 2821.5(8) | 6798.6(5) | 4229.3(3) | 34.86(18) |
| O1 | 4520(6) | 7371(3) | 4981(2) | 42.3(8) |
| O1W | 4713(8) | 7588(4) | 3398(2) | 64.7(13) |
| O2 | 6437(5) | 6656(3) | 5805.9(18) | 43.1(8) |
| N1 | 3922(5) | 5164(4) | 4464(2) | 32.4(9) |
| N2 | 1130(6) | 5833(4) | 3630(2) | 40.4(10) |
| C1 | 4453(7) | 5219(4) | 5222(2) | 34.0(10) |
| C2 | 5868(8) | 4211(5) | 5321(3) | 49.4(14) |
| C3 | 6926(10) | 4217(7) | 4624(3) | 71.2(19) |
| C4 | 5733(8) | 4896(5) | 4097(3) | 48.9(13) |
| C5 | 5197(7) | 6518(4) | 5347(2) | 33.9(10) |
| C6 | 2505(7) | 4210(4) | 4308(3) | 43.0(12) |
| C7 | 1322(7) | 4619(5) | 3698(3) | 39.7(11) |
| C8 | 349(8) | 3822(6) | 3268(3) | 55.7(15) |
| C9 | -832(9) | 4275(7) | 2770(4) | 65.4(18) |
| C10 | -1035(8) | 5526(7) | 2706(3) | 63.2(17) |
| C11 | -29(8) | 6276(6) | 3139(3) | 53.5(14) |
| Cl1 | 5810(2) | 1101.5(14) | 3363.3(8) | 55.2(4) |

Table S26 **Fractional Atomic Coordinates (×10^4) and Equivalent Isotropic Displacement Parameters ($Å^2×10^3$) for CuL-water. $U_{eq}$ is defined as 1/3 of the trace of the orthogonalised $U_{IJ}$ tensor.**

| Atom | *x* | *y* | *z* | U(eq) |
|---|---|---|---|---|
| O3 | 4649(10) | 2063(5) | 3138(3) | 107(2) |
| O4 | 5696(14) | 239(8) | 2801(5) | 175(4) |
| O5 | 7648(7) | 1462(6) | 3410(4) | 115(2) |
| O6 | 5360(19) | 1100(20) | 4084(6) | 131(7) |
| O7 | 5230(20) | 205(11) | 3849(9) | 113(6) |

Table S27 **Anisotropic Displacement Parameters ($Å^2×10^3$) for CuL-water. The Anisotropic displacement factor exponent takes the form: $-2π^2[h^2a^{*2}U_{11}+2hka^*b^*U_{12}+…]$.**

| Atom | $U_{11}$ | $U_{22}$ | $U_{33}$ | $U_{23}$ | $U_{13}$ | $U_{12}$ |
|---|---|---|---|---|---|---|
| Cu1 | 34.0(3) | 30.7(3) | 39.9(3) | -0.2(2) | 0.5(3) | 0.7(2) |
| O1 | 50(2) | 30.7(17) | 46.0(18) | 0.2(15) | -8.0(18) | -2.0(15) |
| O1W | 86(3) | 53(2) | 55(3) | -1(2) | 24(3) | -12(2) |
| O2 | 45.1(18) | 41.3(18) | 42.9(17) | -0.6(18) | -6.0(17) | -10.0(15) |
| N1 | 26(2) | 33(2) | 39(2) | -1.5(16) | 1.6(15) | -2.5(16) |
| N2 | 32(2) | 49(3) | 39(2) | 4.5(19) | 3.6(18) | -3.2(19) |
| C1 | 33(2) | 33(2) | 35(2) | 2.8(19) | 0.0(19) | -3.7(19) |
| C2 | 52(3) | 35(3) | 62(3) | 0(3) | -14(3) | 2(2) |
| C3 | 53(4) | 92(5) | 68(4) | -7(4) | 0(3) | 37(4) |
| C4 | 38(3) | 48(3) | 60(4) | -1(3) | 14(3) | 7(2) |
| C5 | 35(2) | 35(3) | 32(2) | -2.1(19) | 5(2) | -3.8(19) |
| C6 | 39(3) | 33(2) | 57(3) | -2(2) | -3(2) | -5.7(19) |
| C7 | 34(2) | 38(3) | 47(3) | -7(2) | 5(2) | -5(2) |

Table S27 Anisotropic Displacement Parameters ($Å^2×10^3$) for CuL-water. The Anisotropic displacement factor exponent takes the form: $-2π^2[h^2a^{*2}U_{11}+2hka^*b^*U_{12}+…]$.

| Atom | $U_{11}$ | $U_{22}$ | $U_{33}$ | $U_{23}$ | $U_{13}$ | $U_{12}$ |
|---|---|---|---|---|---|---|
| C8 | 44(3) | 58(3) | 65(4) | -13(3) | -3(3) | -8(3) |
| C9 | 46(3) | 88(5) | 62(4) | -20(4) | -9(3) | -12(3) |
| C10 | 39(3) | 91(5) | 60(4) | 0(4) | -12(3) | -6(3) |
| C11 | 42(3) | 66(4) | 52(3) | 7(3) | -1(3) | -2(3) |
| Cl1 | 42.8(7) | 60.9(9) | 61.9(9) | 14.7(7) | -7.2(7) | -8.8(7) |
| O3 | 117(5) | 85(4) | 119(5) | -9(3) | -38(4) | 37(4) |
| O4 | 170(8) | 143(7) | 213(8) | -89(7) | -67(7) | 19(6) |
| O5 | 55(3) | 134(5) | 155(6) | 24(4) | -7(3) | -33(3) |
| O6 | 71(8) | 260(20) | 67(7) | 93(11) | 18(6) | 28(12) |
| O7 | 92(9) | 70(8) | 175(13) | 45(8) | 34(9) | -16(7) |

Table S28 Bond Lengths for CuL-water.

| Atom | Atom | Length/Å | Atom | Atom | Length/Å |
|---|---|---|---|---|---|
| Cu1 | O1 | 1.970(4) | C1 | C5 | 1.535(6) |
| Cu1 | O1W | 2.246(4) | C2 | C3 | 1.516(9) |
| Cu1 | O2[1] | 1.962(3) | C3 | C4 | 1.507(8) |
| Cu1 | N1 | 2.004(4) | C6 | C7 | 1.498(7) |
| Cu1 | N2 | 1.966(4) | C7 | C8 | 1.379(8) |
| O1 | C5 | 1.257(6) | C8 | C9 | 1.358(9) |
| O2 | C5 | 1.251(6) | C9 | C10 | 1.381(10) |
| N1 | C1 | 1.478(6) | C10 | C11 | 1.363(9) |
| N1 | C4 | 1.504(6) | Cl1 | O3 | 1.408(5) |

Table S28 **Bond Lengths for CuL-water.**

| Atom | Atom | Length/Å | Atom | Atom | Length/Å |
|---|---|---|---|---|---|
| N1 | C6 | 1.487(6) | Cl1 | O4 | 1.419(7) |
| N2 | C7 | 1.340(7) | Cl1 | O5 | 1.383(5) |
| N2 | C11 | 1.335(7) | Cl1 | O6 | 1.393(12) |
| C1 | C2 | 1.511(7) | Cl1 | O7 | 1.403(10) |

[1]-1/2+X,3/2-Y,1-Z

Table S29 **Bond Angles for CuL-water.**

| Atom | Atom | Atom | Angle/˚ | Atom | Atom | Atom | Angle/˚ |
|---|---|---|---|---|---|---|---|
| O1 | Cu1 | O1W | 90.12(18) | C1 | C2 | C3 | 103.3(4) |
| O1 | Cu1 | N1 | 83.10(15) | C4 | C3 | C2 | 106.6(5) |
| O2[1] | Cu1 | O1 | 93.78(15) | N1 | C4 | C3 | 106.7(4) |
| O2[1] | Cu1 | O1W | 87.36(17) | O1 | C5 | C1 | 117.8(4) |
| O2[1] | Cu1 | N1 | 167.35(14) | O2 | C5 | O1 | 124.4(4) |
| O2[1] | Cu1 | N2 | 97.36(16) | O2 | C5 | C1 | 117.8(4) |
| N1 | Cu1 | O1W | 104.86(17) | N1 | C6 | C7 | 109.4(4) |
| N2 | Cu1 | O1 | 164.92(16) | N2 | C7 | C6 | 115.2(4) |
| N2 | Cu1 | O1W | 100.48(18) | N2 | C7 | C8 | 121.2(5) |
| N2 | Cu1 | N1 | 83.83(16) | C8 | C7 | C6 | 123.3(5) |
| C5 | O1 | Cu1 | 113.5(3) | C9 | C8 | C7 | 119.4(6) |
| C5 | O2 | Cu1[2] | 116.3(3) | C8 | C9 | C10 | 119.2(6) |
| C1 | N1 | Cu1 | 106.2(3) | C11 | C10 | C9 | 119.1(6) |
| C1 | N1 | C4 | 103.1(4) | N2 | C11 | C10 | 121.8(6) |
| C1 | N1 | C6 | 113.4(4) | O3 | Cl1 | O4 | 103.7(5) |

Table S29 Bond Angles for CuL-water.

| Atom | Atom | Atom | Angle/˚ | Atom | Atom | Atom | Angle/˚ |
|---|---|---|---|---|---|---|---|
| C4 | N1 | Cu1 | 114.5(3) | O5 | Cl1 | O3 | 112.0(4) |
| C6 | N1 | Cu1 | 108.1(3) | O5 | Cl1 | O4 | 107.0(5) |
| C6 | N1 | C4 | 111.5(4) | O5 | Cl1 | O6 | 99.3(7) |
| C7 | N2 | Cu1 | 114.4(4) | O5 | Cl1 | O7 | 116.3(8) |
| C11 | N2 | Cu1 | 126.1(4) | O6 | Cl1 | O3 | 99.1(7) |
| C11 | N2 | C7 | 119.2(5) | O6 | Cl1 | O4 | 135.1(10) |
| N1 | C1 | C2 | 105.2(4) | O7 | Cl1 | O3 | 122.7(7) |
| N1 | C1 | C5 | 106.0(4) | O7 | Cl1 | O4 | 90.2(8) |
| C2 | C1 | C5 | 114.9(4) | | | | |

[1]-1/2+X,3/2-Y,1-Z; [2]1/2+X,3/2-Y,1-Z

Table S30 Torsion Angles for CuL-water.

| A | B | C | D | Angle/˚ | A | B | C | D | Angle/˚ |
|---|---|---|---|---|---|---|---|---|---|
| Cu1 | O1 | C5 | O2 | 172.9(4) | C1 | C2 | C3 | C4 | -17.7(7) |
| Cu1 | O1 | C5 | C1 | -7.1(5) | C2 | C1 | C5 | O1 | 146.9(5) |
| Cu1[1] | O2 | C5 | O1 | -16.2(6) | C2 | C1 | C5 | O2 | -33.0(6) |
| Cu1[1] | O2 | C5 | C1 | 163.7(3) | C2 | C3 | C4 | N1 | -5.8(7) |
| Cu1 | N1 | C1 | C2 | -159.9(3) | C4 | N1 | C1 | C2 | -39.1(5) |
| Cu1 | N1 | C1 | C5 | -37.8(4) | C4 | N1 | C1 | C5 | 83.0(4) |
| Cu1 | N1 | C4 | C3 | 142.4(4) | C4 | N1 | C6 | C7 | -95.0(5) |
| Cu1 | N1 | C6 | C7 | 31.7(4) | C5 | C1 | C2 | C3 | -80.7(5) |
| Cu1 | N2 | C7 | C6 | 11.3(6) | C6 | N1 | C1 | C2 | 81.6(5) |
| Cu1 | N2 | C7 | C8 | -174.3(4) | C6 | N1 | C1 | C5 | -156.3(4) |

Table S30 Torsion Angles for CuL-water.

| A | B | C | D | Angle/˚ | A | B | C | D | Angle/˚ |
|---|---|---|---|---|---|---|---|---|---|
| Cu1 | N2 | C11 | C10 | 174.2(4) | C6 | N1 | C4 | C3 | -94.5(5) |
| N1 | C1 | C2 | C3 | 35.5(5) | C6 | C7 | C8 | C9 | 173.6(5) |
| N1 | C1 | C5 | O1 | 31.2(6) | C7 | N2 | C11 | C10 | 0.4(8) |
| N1 | C1 | C5 | O2 | -148.8(4) | C7 | C8 | C9 | C10 | -0.1(9) |
| N1 | C6 | C7 | N2 | -29.0(6) | C8 | C9 | C10 | C11 | 0.7(10) |
| N1 | C6 | C7 | C8 | 156.7(5) | C9 | C10 | C11 | N2 | -0.9(10) |
| N2 | C7 | C8 | C9 | -0.4(9) | C11 | N2 | C7 | C6 | -174.2(4) |
| C1 | N1 | C4 | C3 | 27.5(6) | C11 | N2 | C7 | C8 | 0.2(8) |
| C1 | N1 | C6 | C7 | 149.1(4) | | | | | |

[1]1/2+X,3/2-Y,1-Z

Table S31 Hydrogen Atom Coordinates (Å×10[4]) and Isotropic Displacement Parameters (Å[2]×10[3]) for CuL-water.

| Atom | *x* | *y* | *z* | U(eq) |
|---|---|---|---|---|
| H1WA | 4720(110) | 7330(60) | 2990(18) | 97 |
| H1WB | 4970(120) | 8330(30) | 3400(30) | 97 |
| H1 | 3366.29 | 5069.21 | 5523.58 | 41 |
| H2A | 5265.88 | 3429.16 | 5399.7 | 59 |
| H2B | 6686.61 | 4385.89 | 5717.73 | 59 |
| H3A | 8113.91 | 4624.01 | 4680.84 | 85 |
| H3B | 7146.62 | 3386.29 | 4462.8 | 85 |
| H4A | 6333.97 | 5651.04 | 3953.26 | 59 |
| H4B | 5524.04 | 4399.07 | 3676.93 | 59 |

**Table S31 Hydrogen Atom Coordinates (Å×10⁴) and Isotropic Displacement Parameters (Å²×10³) for CuL-water.**

| Atom | *x* | *y* | *z* | U(eq) |
|---|---|---|---|---|
| H6A | 1734.59 | 4075.97 | 4723.99 | 52 |
| H6B | 3114.8 | 3445.45 | 4189.84 | 52 |
| H8 | 501.1 | 2981.71 | 3319.02 | 67 |
| H9 | -1495.45 | 3748.57 | 2476.37 | 78 |
| H10 | -1847.32 | 5853.63 | 2372.1 | 76 |
| H11 | -152.85 | 7118.62 | 3090.85 | 64 |

**Table S32 Atomic Occupancy for CuL-water.**

| Atom | *Occupancy* | Atom | *Occupancy* | Atom | *Occupancy* |
|---|---|---|---|---|---|
| O6 | 0.5 | O7 | 0.5 | | |

CuL-water

**Table S33 Crystal data and structure refinement for CuL-water.**

| | |
|---|---|
| Identification code | CuL-water |
| Empirical formula | $C_{11}H_{15}ClCuN_2O_7$ |
| Formula weight | 386.24 |
| Temperature/K | 293(2) |
| Crystal system | orthorhombic |
| Space group | $P2_12_12_1$ |
| a/Å | 7.1965(8) |
| b/Å | 10.9293(13) |
| c/Å | 18.810(2) |
| α/° | 90 |

| | |
|---|---|
| β/° | 90 |
| γ/° | 90 |
| Volume/Å$^3$ | 1479.5(3) |
| Z | 4 |
| $\rho_{calc}$g/cm$^3$ | 1.734 |
| μ/mm$^{-1}$ | 1.693 |
| F(000) | 788.0 |
| Crystal size/mm$^3$ | 0.239 × 0.116 × 0.066 |
| Radiation | Mo Kα (λ = 0.71073) |
| 2Θ range for data collection/° | 5.714 to 51.49 |
| Index ranges | -8 ≤ h ≤ 8, -13 ≤ k ≤ 12, -22 ≤ l ≤ 22 |
| Reflections collected | 9103 |
| Independent reflections | 2813 [$R_{int}$ = 0.0333, $R_{sigma}$ = 0.0306] |
| Data/restraints/parameters | 2813/85/214 |
| Goodness-of-fit on F$^2$ | 1.104 |
| Final R indexes [I>=2σ (I)] | $R_1$ = 0.0331, w$R_2$ = 0.0811 |
| Final R indexes [all data] | $R_1$ = 0.0386, w$R_2$ = 0.0862 |
| Largest diff. peak/hole / e Å$^{-3}$ | 0.41/-0.30 |
| Flack parameter | -0.010(8) |
